# Long Term Response to Circulating Angiogenic Cells, Unstimulated or Atherosclerotic Pre-Conditioned, in Critical Limb Ischemic Mice

**DOI:** 10.3390/biomedicines9091147

**Published:** 2021-09-03

**Authors:** Lucía Beltrán-Camacho, Margarita Jiménez-Palomares, Ismael Sanchez-Gomar, Antonio Rosal-Vela, Marta Rojas-Torres, Sara Eslava-Alcon, Jose Angel Alonso-Piñero, Almudena González-Rovira, Mª Jesús Extremera-García, Rosario Conejero, Esther Doiz, Manuel Rodriguez-Piñero, Martin R. Larsen, Mª Carmen Duran-Ruiz

**Affiliations:** 1 Biomedicine, Biotechnology and Public Health Department, Cadiz University, 11002 Cadiz, Spain; lucia.beltrancamacho@alum.uca.es (L.B.-C.); margarita.jimenezpalomares@gm.uca.es (M.J.-P.); ismael.sanchez@uca.es (I.S.-G.); antonio.rosal@uca.es (A.R.-V.); marta.rojas@uca.es (M.R.-T.); sara.eslava@gm.uca.es (S.E.-A.); joseangel.alonsopi@alum.uca.es (J.A.A.-P.); almudena.gonzalez@uca.es (A.G.-R.); maria.jesus.extremera.garcia@gmail.com (M.J.E.-G.); 2 Institute of Research and Innovation in Biomedical Sciences of Cádiz (INiBICA), 11009 Cádiz, Spain; 3 Angiology & Vascular Surgery Unit, Hospital Universitario Puerta del Mar, 11009 Cádiz, Spain; rosarioconejero@gmail.com (R.C.); edoiz@comcadiz.com (E.D.); manuel.rodriguez.pinero.sspa@juntadeandalucia.es (M.R.-P.); 4 Department of Biochemistry and Molecular Biology, University of Southern Denmark, 5230 Odense, Denmark; mrl@bmb.sdu.dk

**Keywords:** Circulating angiogenic cells, critical limb-threatening ischemia, atherosclerotic secretome, proteomics, neovascularization, immune cells

## Abstract

Critical limb ischemia (CLI), the most severe form of peripheral artery disease, results from the blockade of peripheral vessels, usually correlated to atherosclerosis. Currently, endovascular and surgical revascularization strategies cannot be applied to all patients due to related comorbidities, and even so, most patients require re-intervention or amputation within a year. Circulating angiogenic cells (CACs) constitute a good alternative as CLI cell therapy due to their vascular regenerative potential, although the mechanisms of action of these cells, as well as their response to pathological conditions, remain unclear. Previously, we have shown that CACs enhance angiogenesis/arteriogenesis from the first days of administration in CLI mice. Also, the incubation ex vivo of these cells with factors secreted by atherosclerotic plaques promotes their activation and mobilization. Herein, we have evaluated the long-term effect of CACs administration in CLI mice, whether pre-stimulated or not with atherosclerotic factors. Remarkably, mice receiving CACs and moreover, pre-stimulated CACs, presented the highest blood flow recovery, lower progression of ischemic symptoms, and decrease of immune cells recruitment. In addition, many proteins potentially involved, like CD44 or matrix metalloproteinase 9 (MMP9), up-regulated in response to ischemia and decreased after CACs administration, were identified by a quantitative proteomics approach. Overall, our data suggest that pre-stimulation of CACs with atherosclerotic factors might potentiate the regenerative properties of these cells in vivo.

## 1. Introduction

Peripheral artery disease (PAD) is an under-estimated and under-diagnosed cardiovascular disease (CVD) that is asymptomatic at initial stages [1] and affects more than 202 million people worldwide, approximately 10–15% of the adult population [2]. PAD is caused by the blockade of arteries supplying blood to the upper and lower extremities, mainly as a result of atherosclerotic events, and it correlates with risk factors such as older age, hypertension, dyslipidemia, cigarette smoking, and diabetes [3]. Critical limb ischemia (CLI), the most severe form of PAD, presents as pain while at rest, ischemic ulceration, and/or gangrene in the affected zone [1]. CLI has been associated with an increased risk of amputations (fingers, toes, or extremities) and related mortality [4,5,6]. Moreover, metabolic diseases such as diabetes mellitus (DM) significantly increase the risk of CLI, accelerating the progression and severity of this disease. Despite improvements in endovascular and surgical strategies, conventional revascularization is not possible in 50–90% of patients mainly due to associated comorbidities, and many CLI patients are considered as “no-option” for revascularization [7,8]. As an alternative for those patients that are not suitable candidates for surgery, the use of pro-angiogenic cells such as endothelial progenitor cells (EPCs) has become a promising option, due to their potential to promote neovascularization in ischemic tissues. To date, two different subpopulations of EPCs have been described [9,10,11,12,13]: early EPCs, also called circulating angiogenic cells (CACs) or myeloid angiogenic cells, and late outgrowth EPCs or endothelial colony-forming cells (ECFCs). Both sets of cells have been tested to promote revascularization in ischemic diseases such as myocardial infarction or CLI [14,15,16,17,18,19,20]. EPCs also participate in atherogenesis and arterial healing after vascular injury [21,22,23]. Different studies suggest that CACs participate in vascular repair in a paracrine fashion, while ECFCs, recruited by CACs, might replace damaged endothelium and are probably responsible for new-vessel formation [9,24,25]. For instance, CACs release factors that appear to promote ECFCs tubule formation in vitro [13,24].

Despite encouraging results in pre-clinical and clinical trials, the variability seen between these studies, together with the fact that EPCs become dysfunctional under pathological situations, has somehow slowed down their potential use in the clinic. Nobody seems to doubt their angiogenic properties [25,26]. However, whether using them alone or in combination with other cell types, it is necessary to understand their mechanisms of action, and moreover, how they behave under pathological conditions, in order to implement their use as cell therapy. Based on previous studies, we have focused on evaluating the role of CACs in CLI as well as the effect that an atherosclerotic environment exerts on them [12,13,22]. Very recently, we have shown that in the first days after CACs administration to CLI mice, CACs migrate to the vasculature of ischemic tissues, where they promote an increase in vascular density, compared to untreated mice, by enhancing angiogenesis and arteriogenesis. Furthermore, in the presence of CACs, proteomic changes indicated a decrease in cell death and controlled recruitment of immune cells in the ischemic area, compared to those mice not receiving cell treatment [12].

In addition, we have also described that the incubation of healthy CACs with the factors secreted by atherosclerotic plaques (AP) affects their protein expression pattern, reflecting an initial response to an inflammatory environment, which enhanced their activation and mobilization ex vivo [22]. Moreover, in response to the atherosclerotic secretome, CACs release several proteins that might stimulate an angiogenic switch by impairing ECFCs tubule formation ex vivo [27]. Considering all this, in the current study we have evaluated the effect of CACs administration in the long term in CLI mice. In addition, we have also tested, for the first time, whether the strategy of pre-stimulating CACs ex-vivo with AP secretomes prior to their administration to CLI mice could potentiate their regenerative effect, improving mobilization towards damaged tissue and promoting neovascularization in vivo.

## 2. Materials and Methods

**Figure 1:**
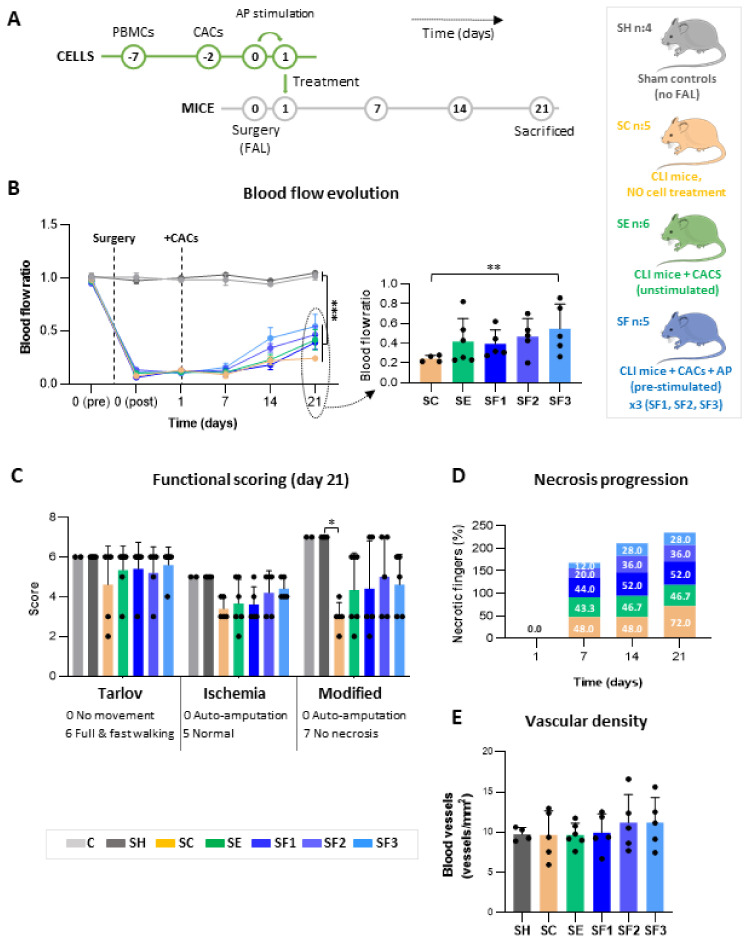
**Experimental workflow and follow-up of ischemic symptoms in CLI mice in response to CACs.** (**A**) Schematic representation of the experimental assay. CACs were isolated from PBMCs of healthy donors, cultured for a week prior to stimulation with the atherosclerotic (AP) secretome, and administered to CLI mice 24 h after femoral ligation (FAL). A follow-up of ischemic symptoms was done on days 1, 7, 14, and 21 after surgery. (**B**) Blood flow changes measured per group within time. Perfusion (PU) averaged ratios of left (injured) vs. right (non-injured) limbs are shown, together with a graph comparing the ratios on day 21 of ischemic mice. (**C**) Motility (Tarlov score) and ischemic (ischemia/modified ischemia scores) changes registered at day 21. (**D**) Necrosis progression, represented as the percentage of necrotic fingers per group, registered on days 1, 7, 14, and 21. (**E**) The number of blood vessels (vessels/mm^2^) was measured by staining left limb muscles with anti-α-smooth muscle actin (α-SMA) antibody. Groups analysed: Healthy controls (C, n:2); Sham, surgery controls (SH, n:4); ischemic mice, untreated (SC, n:5) or treated with unstimulated CACs (SE, n:6) or with CACs pre-stimulated with AP factors (SF: n:15: SF1, n:5; SF2, n:5; SF3, n:5). Data were presented as mean ± SEM (two-way ANOVA and Tukey post hoc; blood flow and functional scoring) or mean ± SD (Kruskal-Wallis and Dunn’s multiple comparisons test; vascular density) (* *p*-value < 0.05, ** *p*-value < 0.01, *** *p*-value < 0.001).

An overview of the experimental workflow and follow-up of ischemic changes is shown in Figure 1A.

### 2.1. Cell Isolation and Culture

CACs were isolated from peripheral blood of healthy donors and cultured as described [12,22]. Non-adherent cells were discarded after 4 days, and attached cells were grown until day 7 in fresh media, reaching approximately 80% confluence, when CACs were used for all experiments. Cell identity was confirmed by flow cytometry and immunohistochemistry (IHC) as previously described [12,13,22]. The full list of antibodies can be found in Supplementary Materials and methods tables.

### 2.2. AP Secretome Acquisition

Atherosclerotic femoral arteries were obtained from patients undergoing surgical endarterectomies at the Hospital Universitario Puerta del Mar, Cadiz, Spain, and cultured as described [13,22]. Final secretomes were collected by centrifugation and the protein content was measured, storing them at −80 °C until further use. In total, CACs were pre-stimulated with the AP secretome of three different donors (patient-1, patient-2, and patient-3). All donors provided informed consent prior to sample collection. The study was approved by the Andalusian Bioethics Committee for Biomedical Research, Consejeria de Salud (Acta 1/2018), and it followed the principles outlined in the Declaration of Helsinki.

### 2.3. Murine Model of CLI. CACs Administration

Balb-C Nude (CAnN.Cg-Foxn1nu/Crl) female mice (n:32), age 9 weeks, were anesthetized with ketamine (100 mg/kg) and xylazine (10 mg/kg), both subcutaneously, before surgery. Ischemia was induced in left limbs by double femoral artery ligation (FAL), occluding the distal and proximal ends with double knots (non-absorbable 6/0), as described [12,28,29].

Twenty-four hours after surgery, ischemic (undergoing FAL) mice received an intramuscular injection of either 50 μL physiological serum without cells (untreated group; SC, n:5), or 50 μL physiological serum containing 5·10^5^ human CACs (basal treated group; SE, n:6) or 5·10^5^ hCACs pre-incubated with AP secretome (pre-conditioned treated group; SF, n:15) (Figure 1A). The intramuscular injection was performed, following the same procedure as in Beltran-Camacho L et al. [12], in order to achieve a maximal concentration of cells within the ischemic area [30,31]. Pre-stimulated cells were incubated with AP secretome (100 µg proteins) 24 h at 37 °C, 5% CO_2_, as described [22], and washed twice with PBS 1X, before injection (5·10^5^ cells/mouse). Mice receiving stimulated CACs (SF; n:15) were distributed in 3 groups depending on the AP secretome source: SF1, from patient-1 (n:5); SF2, patient-2 (n:5); SF3, patient-3 (n:5). Additionally, healthy mice (C, n:2) were used as baseline controls of blood flow and ischemic symptoms. Finally, sham surgical controls (SH; n:4), were used for proteomic assays, vascular density changes, immune cell detection, and cytokine analysis.

Animal experimentation was approved by the Ethical Committee from both the University of Cadiz and the Andalusian Committed of animal experimentation (registration number ES110120000210). The current study followed the standard guidelines for animal research included in the Spanish laws (RD 53/2013) as well as the European Regulations (2012/707/UE).

### 2.4. Follow-Up of Physiological Changes after CLI and Cell Administration

Blood flow was measured at baseline (prior surgery, pre-) and after surgery (post-) for both paws, and also at days 1, 7, 14, and 21 using a Laser Doppler system (Periflux System 5000; Perimed; Järfälla, Sweden). Perfusion was expressed as the ratio of left (ischemic) vs. right (non-ischemic) limbs. In parallel, ischemic symptoms (motility changes, inflammation, ulceration, and necrosis) were registered during the entire experiment using the Tarlov score (motility changes) [28], together with ischemic and modified ischemic scores [29] used in CLI animal models, as described [31] (Supplementary Table S1).

### 2.5. Tissue Extraction

Mice (n:32) were euthanized on day 21 after surgery in a CO_2_ chamber. Left limbs muscles were harvested: low frontal muscles (tibialis anterior) were fixed in 10% formaldehyde for 15 days and then cryoprotected in 30% sucrose in PBS 1X during 24 h. Tissue was then embedded in OCT using tissue molds before congelation for appropriate preservation until sectioning and processing for IHC; low back muscles (gastrocnemius and soleus) and middle muscles (bicep femoris, adductor, and semi-membranous) were snap-frozen in liquid N_2_ and stored at −80 °C for further proteomic analysis and Alu-based quantification respectively.

### 2.6. Immunohistochemical Analysis

IHC was performed with transversal tissue sections, in order to detect cells and vessels in all groups (SH, SC, SE, SF1, SF2, and SF3), as described [12]. Primary antibodies used were anti-CD31 for human endothelial cells detection, anti-α-actin smooth muscle (α-SMA) for vessel detection, anti-MOMA-2 for macrophages/monocytes detection, and anti-Ly-6G for neutrophils detection [32]. Direct labeling with FITC-UEA1 was also used to detect the presence of human cells, as described [12]. Full information regarding antibodies can be found in Supplementary Materials. In total, 3 tissue sections (8 μm each) separated by 40 μm between them were used per mouse for cellular and blood vessel detection, analyzing the entire tissue areas by fluorescence microscopy. Images were acquired at 20×, using an MMI CellCut Plus (Olympus), and visualized with the Zen 2 (Zeiss) software. Results were expressed as the number of cells per mm^2^, the number of blood vessels per mm^2,^ or blood vessels diameter (μm).

### 2.7. Alu-Based Quantification

Quantification of human DNA (hDNA) was carried out with amplification of specific Alu sequences by qPCR, as described [12,29,33]. Briefly, genomic DNA from the middle muscles was extracted with Quick-DNA™ Midiprep Plus Kit (ZymoResearch; Irvine, CA, USA) after Proteinase K treatment. Alu sequences were amplified in 100 ng of genomic DNA by qPCR using TaqMan Universal Master Mix II (Thermo-Fisher 4440043; Waltham, MA, USA), using the primers and hydrolysis FAM label probe described by Funakoshi K et al. [34]. qPCR was done with a CFX Connect Real-Time System (Biorad; Hercules, CA, USA), and the amount of hDNA detected in 100 ng of total genomic DNA (from ischemic muscles) was measured, as previously described [12,31].

### 2.8. Proteomic Analysis

In order to identify proteins differentially expressed between ischemic vs. non-ischemic conditions, and moreover, in response to CACs treatment (with/without atherosclerotic pre-conditioning), proteins in all groups (SH, SC, SE, SF1, SF2, and SF3) were analyzed by mass spectrometry (MS), using a based label-free quantitative approach, as described [12]. Thus, low back muscles were resuspended in lysis buffer (1% NP40, 50 mM HEPES pH 7, 150 mM NaCl, and 1 mM EDTA, supplemented with protease inhibitors) and homogenized by the mechanical procedure for protein extraction. Proteins (100 μg) were precipitated with 100% acetone overnight at −20 °C. Pellets were resuspended in 8 M urea, reduced (10 mM DTT), and alkylated (50 mM IAA) before in-solution digestion with Lys-C (0.04 AU/mg) for 2 h at RT. Samples were diluted four times with 50 mM ammonium bicarbonate and trypsin digested (enzyme/substrate ratio 1:50) at RT overnight. Finally, the digestion was quenched with 0.1% TFA before peptide purification with C18 columns.

Peptide mixtures were analyzed by nano-LC-MS/MS on an Orbitrap Q-Exactive HF-X (Thermo Scientific; Waltham, MA, USA) coupled to an EASY-LC 1000 system (Thermo Scientific; Waltham, MA, USA). Peptides were loaded onto a two-column setup in A-buffer (0.1% FA) using a Luna Omega (1.6 μm) column, and eluted with a 250 nL/min flow rate with an increasing gradient of B-buffer (95% ACN and 0.1% FA), starting at 2% B, going to 45% B in 80 min and to 98% B in 90 min. The Q-Exactive was operated in data-dependent acquisition mode using the following settings: full-scan automatic gain control (AGC) target 3e6 at 120,000 FWHM resolution; scan range 350–1400 *m*/*z*; Orbitrap full-scan maximum injection time 100 ms; MS2 scan AGC target 1e5 at 15,000 FWHM resolution; maximum injection 50 ms; normalized collision energy 30; dynamic exclusion time 20 s; isolation window 1.2 *m*/*z*; 20 MS2 scans per full scan. Duplicates for each biological sample were run by MS.

Data analysis was done with Proteome Discoverer v.2.4 (Thermo Scientific; Waltham, MA, USA). The database search was performed with Mascot v.2.6 against the Swiss-Prot database (taxonomy, Mus musculus; 54,424 protein entries). The following criteria were used: fixed modification: carbamidomethylation (C); dynamic modifications: oxidation (M); precursor mass tolerance 10 ppm; fragment mass tolerance 0.06 Da; enzyme: trypsin; and 2 missed cleavages were allowed. Data filtering was performed using a percolator, resulting in a 1% false discovery rate. Additional filters were also used: search engine rank 1 peptides and Mascot ion score >20. Differentially expressed proteins were defined as follows: *p*-value < 0.05 and fold-change rates > 1.5 (up-regulated) or <0.6 (down-regulated). Data processing and graphs were done with Proteome Discoverer (hierarchical cluster, volcano plots, and principal component analysis, PCA). The Ingenuity Pathway Analysis (IPA; Qiagen; Venlo, The Netherlands) platform was used for functional classification analysis.

### 2.9. Cytokine Expression Analysis

Mouse cytokines (62) were measured in the ischemic tissues on day 21 using a Cytokine antibody array (Mouse Cytokine Antibody Array C3; RayBiotech; AAM-CYT-3; Peachtree Corners, GA, USA), following the manufacturer’s instructions. Briefly, the protein fractions from back muscle tissues (left limbs) were used for cytokine detection, pooled into five groups (SH, SC, SE, SF1, SF2) with equal amounts of protein per sample (200 µg in total). Additionally, Transforming Growth Factor β1 (TGFβ1) levels were evaluated by ELISA (Cusabio; CSB-E04726m; Wuhan, China) following the manufacturer´s guidelines. Again, pooled samples from all groups were used (SH, SC, SE, SF1, SF2, SF3), including duplicates per group. Cytokine levels were compared between all groups vs. SH, and changes were defined with fold-change rates >1.5 (up-regulated) or <0.6 (down-regulated). Hierarchical clustering was done using Perseus software (v1.6.0.2) [35].

### 2.10. Statistical Analysis

Protein-related statistics were obtained with Proteome Discoverer 2.4 and IPA software, while functional-related statistics were performed with GraphPad Prism 8 software. The normal distribution of the data was analyzed by Shapiro–Wilk test, applying then either Kruskal–Wallis test and Dunn’s test as post hoc analysis, or the two-way ANOVA test completed with Tukey’s multiple comparisons test for post hoc analyses. Data were presented as mean ± SEM (parametric) or mean ± SD (non-parametric tests), accordingly. Differences were considered statistically significant when *p*-value < 0.05.

## 3. Results

### 3.1. CACs Promote Blood Flow Recovery in CLI Mice

In response to FAL, blood flow decreased significantly (>80%) in all animals (day 0; SC, SE, and SF) compared to baseline (C, SH), and blood flow remained lower until day 7 when perfusion ratios started to increase. A slight recovery of blood flow was registered in all ischemic limbs by day 14 (Figure 1B). At this time, mice receiving pre-stimulated CACs (SF) showed higher perfusion ratios (although not significantly) than those with un-stimulated CACs (SE) and untreated mice (SC). By day 21, all sets of CACs-treated animals (SE and SF) had higher blood flow ratios than untreated SC, with the SF groups presenting the highest recovery on average. Among them, only mice receiving CACs stimulated with the AP secretome from patient-3 (SF3) showed significant perfusion recovery compared to untreated SC (*p*-value: 0.0052) (Figure 1B), while mice treated with CACs pre-incubated with the patient-1 AP secretome (SF1) had lower blood flow ratios than SF2 and SF3, but similar values to SE mice, without stimulation. Finally, SE perfusion ratios were also higher (not significantly) than SC, and they did not reach the levels seen in the SF group.

### 3.2. CACs Delays Ischemic Progression in CLI Mice

Different ischemic symptoms (i.e., reduced motility, ulceration, necrosis) were reported from day 1 after FAL compared to pre-surgery (Supplementary Figure S1), although the progression of ischemia was lower in CACs-treated mice (SE, SF) than in un-treated SC (Supplementary Figure S1B–D). After surgery (day 1), FAL-mice (SC, SE, and SF) showed inflammation along the injured limb, some black nails, and reduced motility, progressing to necrotic fingers in many cases (Figure 1C). Overall, the SC group presented the worst prognosis, with the highest percentage of necrotic fingers (Figure 1D). By day 21, SC mice presented a significant worsening (necrotic fingers, ulceration) compared to shams, non-FAL controls (SH, *p*-value: 0.013), and showed less motility (Tarlov score) and worse ischemic symptoms than CACs-treated mice (Figure 1C), with 72% of necrotic fingers vs. 46.65% in SE or 38.67% in SF groups (on average) (Figure 1D). On the contrary, motility was less affected in CACs-treated mice (SE and SF) and no progression of ischemic symptoms was seen from day 7 onwards (Figure 1C). Finally, no significant differences were found regarding body weight after surgery, apart from day 1, therefore weight changes correlated to the surgical process itself (Supplementary Figure S1E).

### 3.3. Vascular Density Stabilizes at Day 21

By day 21, the number of vessels (vessels/mm^2^) was similar in all groups (Figure 1E), with no significant differences between them apart from a slightly higher percentage of vessels in SF mice (SF2, SF3), suggesting further vasculogenesis in these animals. Regarding internal diameters, similar values were seen for all conditions too (Supplementary Figure S2A,B), with narrower diameters in all FAL-mice compared to shams.

### 3.4. Human CACs Are Not Present in Ischemic Tissues after 21 Days Post-Transplantation

No traces of hDNA were found on day 21 post-transplantation, after qPCR quantification of human-specific Alu sequences (Supplementary Figure S2C). Likewise, IHC-based cell tracking assays reported similar results, not detecting CD31+ or UEA-1+ human cells (endothelial-specific markers) within the tissues analyzed (Supplementary Figure S2D,E).

### 3.5. Proteomic Changes Related to Long-Term CLI and CACs Treatment

**Figure 2:**
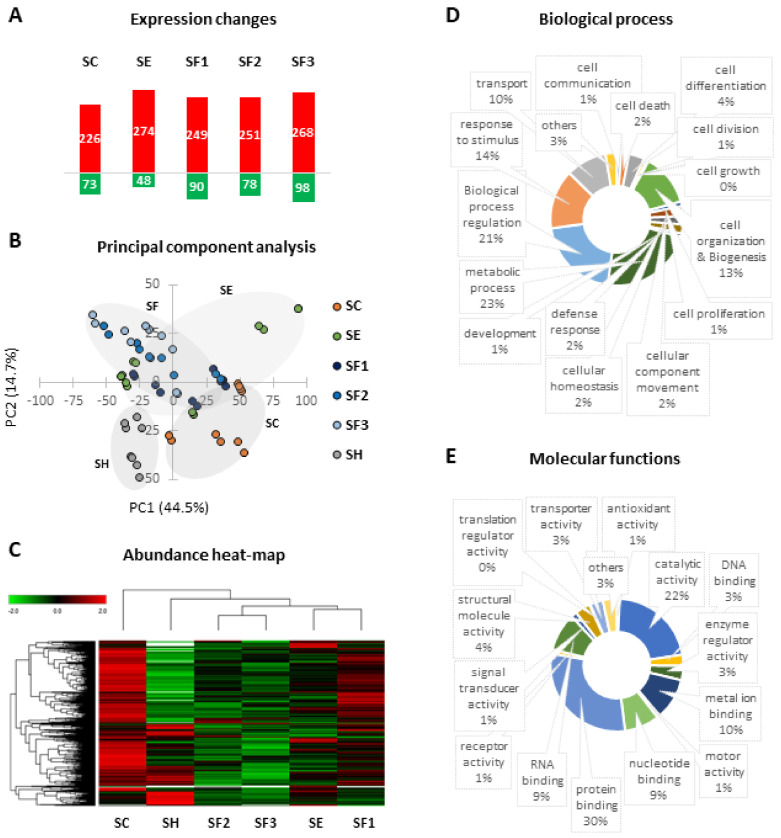
**Summary of proteomic changes.** (**A**) The number of proteins up (red) and down-regulated (green) in each group (SC, SE, SF1, SF2, and SF3) compared to shams (SH). (**B**) Based on protein profiles, PCA clearly distributed mice between the 4 main groups analyzed. (**C**) Hierarchical clustering comparing the proteins patterns of all groups. Finally, a classification of identified proteins according to the (**D**) biological process or the (**E**) molecular functions assigned to these proteins is shown, reported by the Gene Ontology Database (via Proteome Discoverer). Groups analyzed: Sham, surgery controls (SH, n:4); ischemic mice, untreated (SC, n:5): ischemic mice with unstimulated CACs (SE, n:6) or pre-stimulated with atherosclerotic factors (SF: n:15: SF1, n:5; SF2, n:5; SF3, n:5).

In total, 3290 proteins were identified by proteomic analysis, including many proteins up- or down-regulated in response to ischemia and/or CACs treatment pre-stimulated or not with AP factors, taking shams as the baseline expression group (Figure 2A). Representative volcano plots are shown in Supplementary Figure S3. Remarkably, a PCA classification based on differential protein expression (Figure 2B) clearly discriminated between SH and ischemic untreated mice (SC), and moreover, between ischemic CACs-treated groups (SE and SF) and SC, suggesting that protein changes were also affected by atherosclerotic pre-stimulation of CACs. The differential protein patterns seen between all groups were also reported in a hierarchical cluster (Figure 2C). SC and SF1 (with the lowest perfusion recovery from the SF mice) protein profiles were different than the other groups, while SF2 and SF3 had intermediate profiles between SE and SH (in agreement with the higher perfusion recovery seen for these groups). All identified proteins appeared to participate in many biological processes (Figure 2D) and molecular functions (Figure 2E), according to Gene Ontology Database. Complete information regarding quantification data is included in Supplementary Table S2.

#### 3.5.1. Long-Term Protein Changes Due to Ischemia

**Figure 3:**
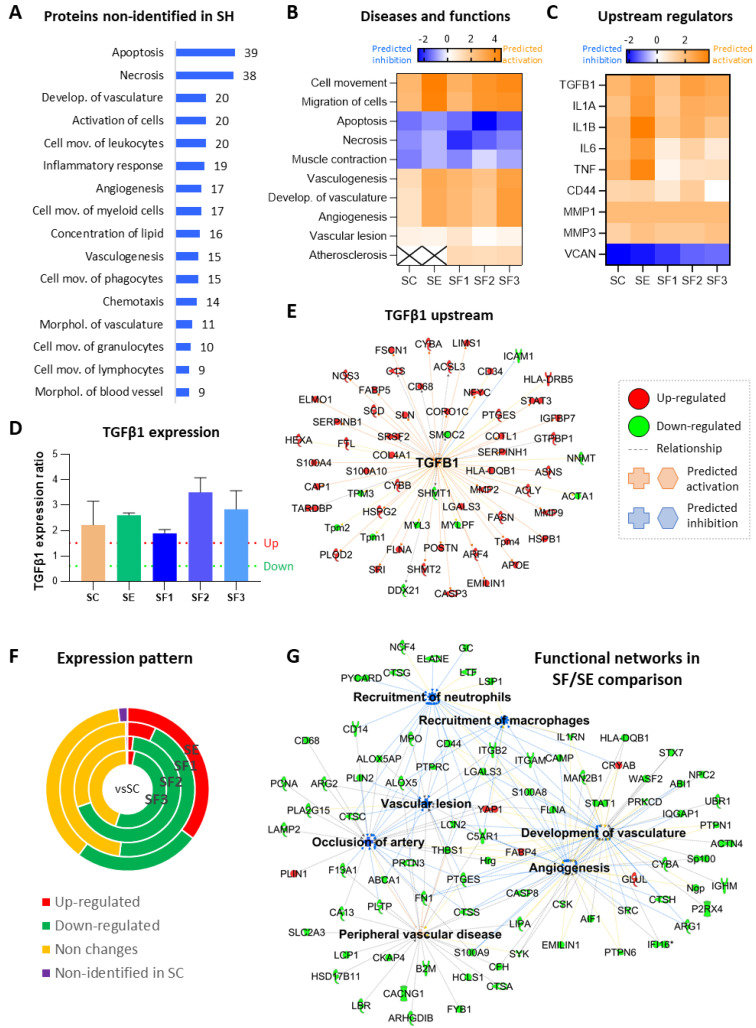
**Proteomic changes in response to ischemia and long-term treatment with CACs.** (**A**) Functional classification of proteins not identified in SH mice. The classification was made with IPA, which also provided the heat maps shown in (**B**) with the major functions and diseases and (**C**) upstream regulators related to proteins differentially expressed in SC, SE, and SF vs. SH (based on z-scores probability values). (**D**) TGFβ1 levels were analyzed by ELISA in muscle limbs on day 21. (**E**) IPA functional network built with TGFβ1-related proteins related which were altered in response to ischemia (SC, SE, and SF, vs. shams). (**F**) Circular chart representing the percentage of proteins altered in CACs-treated (SE and SF) vs. un-treated SC mice. (**G**) IPA functional network including the major functions correlated with the proteins differentially expressed in SF vs. SE. Groups analyzed: Sham, surgery controls (SH, n:4); ischemic mice, untreated (SC, n:5): ischemic mice with unstimulated CACs (SE, n:6) or pre-stimulated with atherosclerotic factors (SF: n:15: SF1, n:5; SF2, n:5; SF3, n:5).

Focusing on proteins differentially expressed, common changes were identified in all mice that underwent FAL (SC, SE, and SF) compared to shams, including 107 proteins not detected in these controls (Figure 3A), therefore representing ischemic dependent alterations (Table 1).

**Figure 4:**
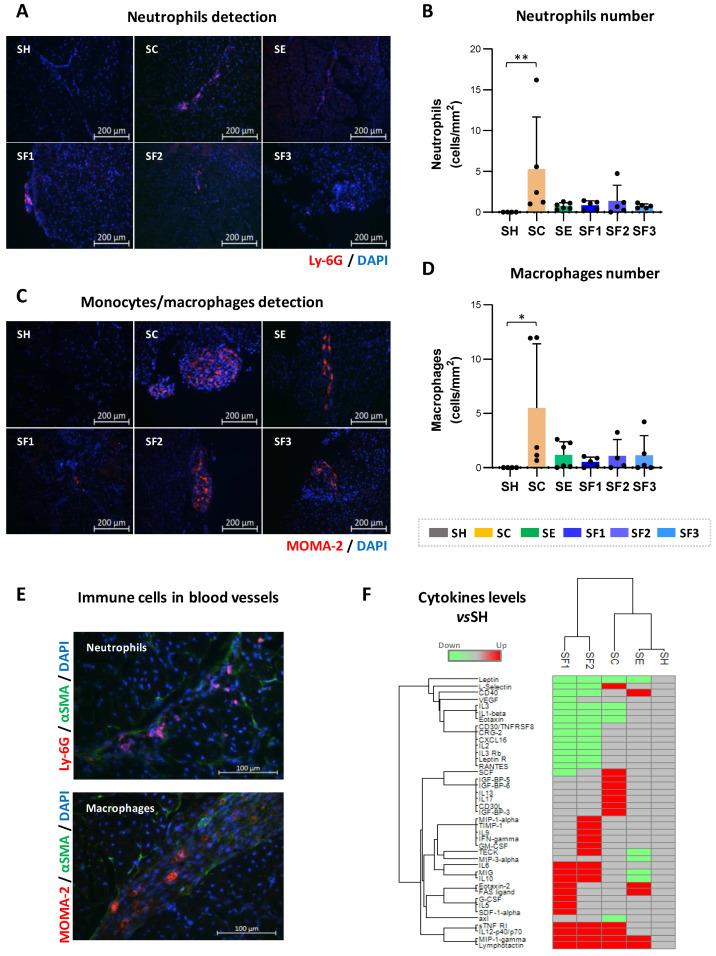
**CACs administration modulates the recruitment of neutrophils and macrophages to the ischemic tissues.** (**A**) Neutrophils (Ly-6G, red) and (**C**) macrophages (MOMA-2, red) were detected on day 21 by IHC in limb muscles of ischemic mice but not in shams. The number of (**B**) neutrophils and (**D**) macrophages (cells/mm^2^) was measured in all groups per tissue area and represented as cells/mm^2^. (**E**) Both, neutrophils Ly-6G (red) and macrophages MOMA-2 (red) were highly present in blood vessels, which were identified with anti-α-SMA antibodies (green). (**F**) Hierarchical cluster with differential cytokines levels found vs. SH group. Groups analyzed: Sham, surgery controls (SH, n:4); ischemic mice, un-treated (SC, n:5): ischemic mice with unstimulated CACs (SE, n:6) or pre-stimulated with atherosclerotic factors (SF: n:15: SF1, n:5; SF2, n:5; SF3, n:5). Data were presented as mean ± SD. Significant differences were seen by Kruskal-Wallis and Dunn’s multiple comparisons test (* *p*-value < 0.05, ** *p*-value < 0.01).

Additionally, 41 of the 62 angiogenic cytokines analyzed (Supplementary Figure S4) were altered in ischemic mice, with SF groups (receiving pre-stimulated CACs) showing the major number of cytokine alterations (Figure 4F). Remarkably, Leptin was down-regulated while Lymphotactin and Macrophage inflammatory peptide gamma (MIP-1-γ/CCL9) appeared up-regulated in all ischemic mice, even 21 days after FAL. Other cytokines were or up- (IL13, IL17, IGF-BP-5, IGF-BP6) or down-regulated (AXL) only in untreated mice (SC) but not in those receiving cell therapy.

Overall, according to IPA functional analysis, a bioinformatics tool based on specific algorithms and integrated databases mainly focused on biomedical literature, the molecular changes related to ischemia (SC, SE, SF vs. SH) represented a potential activation of vasculogenesis and angiogenesis (ANXA1, CD34, MMP9, S100A4), together with an increase of cell movement and migration, and inhibition of apoptosis/necrosis (Table 1 and Figure 3B). IPA also highlighted some potential upstream regulators in ischemic mice (Figure 3C), such as transforming growth factor β1 (TGFβ1), Interleukin 1α (IL1α), IL1β, IL6, or tumor necrosis factor (TNF). Indeed, 59 proteins up- or down-regulated after ischemia (vs. SH) were associated with TGFβ signaling (Figure 3E). An ELISA assay confirmed the increase of TGFβ1 in CACs treated mice (slightly increase in SE, and higher in SF2 and SF3 mainly, compared to SC) (Figure 3D).

#### 3.5.2. CACs Long Term Effect at the Molecular Level

Regarding cell treatment, a differential protein expression profile was seen on day 21 in CACs treated mice (SE, SF) vs. untreated SC, including many proteins overexpressed in SE/SC but not in SF/SC (with any patient donor) (Figure 3F and Table 2).

Overall, processes like vasculogenesis, angiogenesis, or cell movement, appeared more pronounced in response to CACs, according to IPA (Figure 3B), although many of the related proteins were commonly altered in SC and SE (MMP9, CASP3, PAK1, MCPT4, EMILIN1, among others), while others such as S100A8 or Cathelicidin-related antimicrobial peptide (CRAMP) appeared highly up-regulated in SE vs. SC.

Similarly, although cell death was down-regulated in response to ischemia in the long term, the apoptotic/necrotic process was slightly lower in SE, with several related proteins up-regulated only in these mice (APAF1, SLC2A3, MBOAT7, S100A8, CRAMP). Other functions significantly up-regulated in SE vs. SC were endothelial cell activation or Nitric Oxide/Reactive Oxygen Species (NO/ROS) production. Again, some of the proteins involved were Matrix metalloproteinase 9 (MMP9), CD44, or CRAMP.

#### 3.5.3. CACs Atherosclerotic Pre-Conditioning Effect

Atherosclerotic pre-conditioned CACs also promoted protein expression changes in SF vs. SE mice (Supplementary Table S3 and Figure 3G). For instance, some proteins related to atherosclerosis were down-regulated in SF, such as ARG2, CD14, CD44, engulfment, and cell motility protein 1 (ELMO1), neutrophil gelatinase-associated lipocalin (LCN2), MPO, S100A8, or S100A9 (Supplementary Table S3). Likewise, several cytokines were up- (IL6 or IL10) or down-regulated (IL2, IL3Rb, or RANTES/CCL5) only in SF mice.

Overall, according to IPA, protein changes correlated with a significant decrease of cell viability, cell movement, and inflammatory response in SF mice compared to SE (Supplementary Table S3), together with a significant decrease of neutrophils and macrophages recruitment (CAMP, CD44, ITGAM, ITGB2). These changes might be also associated with down-regulation of the atherosclerotic process and for instance, artery occlusion (FABP4, ABCA1, ARG2, LCN2).

Finally, several differences were also identified within the SF groups, including many proteins up-regulated in SF1 compared to SF2 and SF3 groups, related to lipid and carbon metabolism, diabetes or insulin resistance, among others: Acetoacetyl-CoA synthetase (AACS), Acetyl-CoA carboxylase (ACACA), Carnitine O-palmitoyl-transferase 1 (CPT1), 1-acylglycerol-3-phosphate O-acyltransferase (AGPAT2) (Supplementary Table S4).

### 3.6. CACs Treatment Decrease Recruitment of Immune Cells

A high number of neutrophils (Ly-6G+ cells, *p*: 0.019) (Figure 4B) and macrophages (MOMA-2 +, *p*-value: 0.021) (Figure 4D) were found by IHC (Figure 4) in ischemic limbs (on day 21 post-surgery) of SC untreated mice vs. shams. Indeed, these cells, which were mostly detected in blood vessels (Figure 4E), were barely present in SH, confirming the mobilization of immune cells after ischemia. On the other hand, and in agreement with the proteomic results, in response to CACs the number of neutrophils and macrophages found within the ischemic tissues was much lower than in SC, with similar cell numbers in both unstimulated (SE) or pre-conditioned CACs treated (SF) mice. Thus, in the long term, CACs appeared to modulate the presence of immune cells in the injured area.

Finally, positive correlations were reported between the number of neutrophils and macrophages found on day 21 (R:0.417; *p*-value:0.022), as well as a negative correlation between blood flow ratios and neutrophils (R:−0.563; *p*-value:0.001) and macrophages numbers (R:−0.423; *p*-value:0.020).

## 4. Discussion

Angiogenic cell therapy constitutes a potential alternative for ischemic diseases, reflected by the growing number of clinical trials testing different stem and progenitor cells in CLI patients [33,36,37]. Still, the mechanisms of action of these cells and their response under pathological environments remain unclear, representing two major questions that need to be solved prior to their application in clinical practice. Previously, we demonstrated the potential of CACs in promoting, on the first days after their administration to CLI mice, an increase in vascular density, enhancing the maturation of the newly formed vasculature [12,38]. In the long term (21 days after surgery), perfusion recovery was higher in CLI mice receiving CACs (SE and SF) than untreated mice (SC), in agreement with previous studies [26,39,40]. Also, despite a general presence of ischemic symptoms (ulceration, necrosis, motility impairment) right after FAL (days 1–7), the progression was slower after CACs treatment. Moreover, an arrest of these symptoms was seen in CACs-treated mice from day 14, while in SC the percentage of necrosis and other ischemic factors got worse. Remarkably, perfusion recovery was even more significant in mice receiving atherosclerotic pre-conditioned CACs (SF), which also had the lowest progression of ischemic symptoms.

On the other hand, despite the higher perfusion rates found, the final vascular density levels were similar for all groups on day 21 (compared to shams or SC mice). These results suggest that, after the initial vessel sprouting seen in CACs-treated mice at early stages [12], vessel pruning and vascular normalization might have occurred in response to CACs, in order to achieve an optimal vascular density and for instance, effective tissue revascularization [12,41,42].

According to our early day’s results, CACs migrate to the ischemic tissues right after administration [12], are detected nearby the vasculature of damaged tissues 2–4 days after transplantation. However, no hCACs were found by day 21, suggesting that the injected cells are either replaced by host cells or, otherwise, they die during the pruning process or as a result of their low proliferative profile [43,44]. Overall, these results reinforce the hypothesis that CACs-mediated revascularization is mainly achieved in a paracrine fashion. Thus, CAC’s role is fundamental on the first days, when they participate in recruiting autologous progenitor cells [39,40,41] and promoting a mature and functional vascular network, in agreement with in vitro results where CACs secreted factors enhance ECFCs tubule formation [13,26,45].

### 4.1. Long Term Protein Changes Related to Ischemia

**Figure 5:**
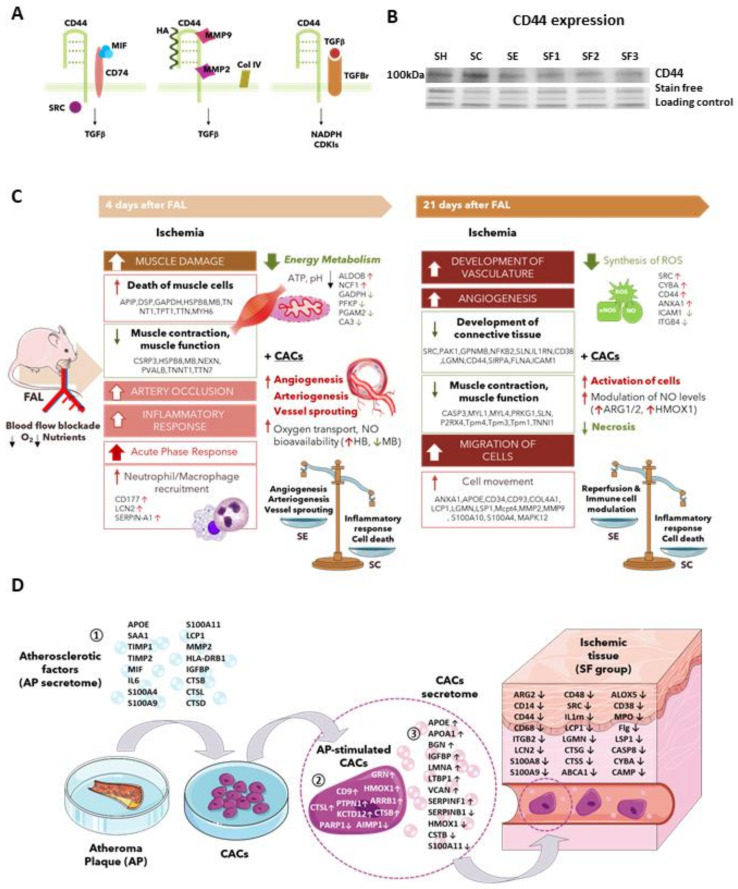
**Potential mechanisms altered after ischemia and CACs therapy.** (**A**) CD44 interactions with other proteins identified, based on literature searches. (**B**) Up-regulation of CD44 in SC mice was confirmed by WB. (**C**) Early and late molecular response of CLI mice with and without CACs treatment (**D**) Proteins involved in pre-stimulation of CACs process. Atherosclerotic plaques (AP) composition, ex vivo CACs response to an atherosclerotic environment, and in vivo modifications after pre-stimulated CACs administration over CLI mice. ①Duran et al., 2003 [63]; ②Vega et al., 2017 [22]; ③Eslava-Alcon et al., 2020 [13].

FAL-induced ischemia triggered a considerable number of molecular changes that were further evaluated with the functional bioinformatics tool IPA. Thus, considering the changes found, many proteins related to artery occlusion, vascular lesion, or muscle damage were identified in all ischemic mice at early stages, together with others related to increased angiogenesis and vasculogenesis (Figure 5). Similarly, a cascade of molecular processes related to ischemia took place right after surgery [12], including down-regulation of energy-metabolism related proteins like several glycolytic enzymes (GADPH, PFKP, PGAM2) or carbonic anhydrase (pH dysregulation) among others, but also upregulation of inflammatory response, with many proteins potentiating neutrophils/macrophage recruitment to the inflammation sites (CD44, LCN2, CD177). The down-regulation of HSPG2 and Collagen IV (principal components of the basal lamina) on days 2–4, might reflect the degradation of the vascular basement membrane (BM) required for immune cells mobilization. Interestingly, the levels of these and other related proteins (HSPG2, Laminin, Collagen IV, Nidogen 1) were restored after 21 days in all groups. For instance, up-regulation of proteins like procollagen-lysine, 2-oxoglutarate 5-dioxygenase 2 (PLOD2), required for the proper synthesis of collagen (hydroxylation step), could have contributed to restoring Col IV levels.

After 21 days of FAL, IPA software reported an increased cell mobilization, mainly of immune cells, in all ischemic mice, together with up-regulation of vasculature development, angiogenesis, and vasculogenesis. Remarkably, 34 proteins over-expressed 2–4 days after FAL (CD44, ANX1, LGALS3, Filamin-A) [12] were also found up-regulated after 21 days, as well as 107 proteins barely detected in shams, representing ischemic dependent changes. Proteins like SAA4, cystatin-C orβ2-macroglobulin, known biomarkers of CVDs [42,45] were highly overexpressed in all ischemic mice, and their levels remained even in presence of cell treatment. Other proteins like AXL appeared only down-regulated in untreated mice. They all have been associated with the atherosclerotic process (SAA [46]) or the severity of PAD (cystatin-C, β2-macroglobulin, AXL [47,48,49]). Thus, although further validations might be required, some of these proteins could be considered as potential markers of CLI.

In addition, many proteins were only found overexpressed in the long term, such as MMP9, MMP2, caspase 3 and 6 (CASP3/6), or mast cell protease 4 precursor (MCPT4), in all mice that underwent FAL. Among these, MMPs have been associated with atherogenesis and PAD [50]. Indeed, increased circulating levels of MMP2 and MMP9 are found in PAD patients, in correlation with the presence of ischemic tissue [51,52,53,54,55]. MMP2 and mainly MMP9 have been described as effectors and regulators of inflammation [56], and they can exert both angiogenic and anti-angiogenic processes [57]. MMP9 is also upregulated during wound healing, participating in tissue repair [58,59,60]. MMP9 activity depends, among others, on its binding to CD44, another protein overexpressed in response to ischemia (Figure 5A,B). CD44 facilitates MMP9 anchoring into the membrane, potentiating the degradation of the vascular BM during trans-endothelial migration of leukocytes from blood vessels to the inflammation sites [61]. CD44, for instance, participates in the inflammatory response, neutrophil mobilization, and recruitment, ECM remodeling, or angiogenesis [12]. Its upregulation in post-ischemic tissues has already been described [61]. CD44 constitutes a cell surface receptor for hyaluronan but also interacts with MMP9 or the macrophage migration inhibitory factor (MIF) via CD74 among others [62] (Figure 5A). Thus, proteins like CD44 and MMP9 should play a role in enhancing ECM degradation, immune cell recruitment and translocation, and further reorganization of BM during revascularization. Further studies might help to elucidate their involvement in CLI.

### 4.2. Molecular Changes in Response to CACs

CACs administration promoted, at the first days, protein changes related to oxidative stress and NO bioavailability (HB, MG, HPX), while in SC mice (untreated) this effect was not detected (at least not from the proteins identified) [12]. NO participates in the regulation of vascular tone, scavenging of ROS, or stimulation of endothelial cell regeneration, among others [64]. Indeed, NO seems critical for EPCs biology and function in sites of active vascularization [65]. Thus, CACs might help to modulate NO levels in an attempt to restore oxygen and remove excessive cytotoxic agents (i.e. free heme). Future research should confirm such a hypothesis. Also, CACs affected proteins involved in the modulation of macrophage/neutrophils recruitment (SERPINA1, ANXA1) [12]. This, together with the potentiation of an optimal vasculature sprouting/maturation, might explain the lower necrotic ratios seen in CACs treated mice (except SF1) compared to SC along with the assay. In this regard, it seems crucial to avoid or delay cell/tissue necrosis until blood flow is restored, otherwise, necrotic cells become unable to participate and/or enhance revascularization/recovery [66,67].

In the long term, many proteins up-regulated in response to ischemia (at early and long-term), were lower in CACs-treated mice, although still higher than in shams (ANXA1, APOE, CTS-Z, CD44, S100A4, S100A9, S100A11). On the contrary, proteins like CRAMP, APAF1, or S100A8, were up-regulated only in SE mice (not in SC). Thus, CACs somehow affected several processes within the ischemic tissues, promoting lower apoptosis/necrosis (APAF1, SLC2A3, MBOAT7, S100A8, CRAMP), up-regulated endothelial cell activation, or NO/ROS production in SE mice compared to untreated ones (i.e., MMP9, CD44 or CRAMP). Again, processes related to vasculature development were slightly more up-regulated in CACs-treated mice (SE and SF), corroborating the initial sprouting and further vessel reorganization described [12]. Altogether, these molecular alterations might explain how CACs improved blood flow recovery while limiting inflammatory cell activation [68] (Figure 5C).

Remarkably, IPA highlighted TGFβ as a major upstream regulator in ischemic mice, after identifying many proteins correlated to TGFβ1, including MMP9, MMP2, or CD44 (Figure 3E). Indeed, CD44-MMP9 interaction promotes activation of TGFβ and activation of angiogenesis, among others [69] (Figure 5A). TGFβ up-regulation was confirmed by ELISA, mainly in SE, SF2, and SF3 mice (Figure 3D). Similarly, the TGFβ1 signaling pathway was found up-regulated in CACs pre-stimulated with AP secretome ex vivo [13]. Considering all these data, and the role assigned for TGFβ1 as the main regulator of blood vessel development and maintenance [70,71], we could postulate that TGFβ1 might play a key role in response to CACs, through mechanisms involving the interaction of proteins like MMP9 and CD44. As stated before, future research should confirm such hypotheses.

Likewise, CRAMP was also upregulated in SE while down-regulated in SC mice (vs. SH). CRAMP, known as an antimicrobial peptide, has additional activities such as chemo-attraction, immune cell activation, or angiogenesis [72]. Up-regulation of CRAMP in CACs-treated mice could represent a protective effect against apoptotic-related ischemia.

Finally, up-regulation of granulocyte colony-stimulating factor (G-CSF) in CACs-treated mice (Supplementary Figure S4), supported the higher perfusion ratios seen in these mice. CACs are known to secrete G-CSF to the circulating medium [41]. Moreover, co-administration of G-CSF and cell therapy enhances, among others, the mobilization of EPCs from the bone marrow into peripheral blood, and mobilized EPCs specifically home to sites of nascent neovascularization, thereby contributing to vascular repair [73,74].

### 4.3. CACs Pre-Stimulation with Atherosclerotic Plaque Secretomes

Next, we tested how the pre-stimulation of CACs with the factors released by atherosclerotic plaques, termed here as the AP secretome, could influence their effect over CLI mice, based on previous results suggesting the activation of CACs in response to an atherosclerotic environment ex vivo [13,22]. We and other authors have evaluated the AP secretome composition [27,63], identifying several biomarkers of atherosclerosis, inflammation, or even vasculogenesis. Among them, several chemotactic proteins up-regulated in the AP secretome, such as MIF, lymphocyte cytosolic protein 1 (LCP1), or IL6, have been described [22] (Figure 5D).

Interestingly, SF mice had on average higher perfusion ratios than SE (mice with unstimulated CACs) or untreated SC. Also, many proteins down-regulated in SF vs. SE mice correlated with down-regulation of cell movement and related inflammatory response, and a decrease in cell viability and atherosclerosis-related processes. Elevated levels of FasL in SF (Figure 4F) might correlate with increased apoptosis in these tissues. Also, proteins like fatty acid-binding protein (FABP4) [75] or perilipin-1 (PLIN1) [76] appeared up-regulated, but mostly down-regulated (ALOX5, ALOX5AP, ARG2, CD44, or LCN2) in SF mice, together with several cathepsins (CTSA, CTSC, CTSD, CTSG, CTSH, and CTSS) or S100 family members (S100A8 and S100A9). The involvement of these and other proteins in atherosclerosis has been previously described [27]. Likewise, proteins also related to DM (APOE, APOD, CD44, CTSD, CTSS, MMP9, or S100A4), were significantly decreased in SF vs. SE mice.

Besides, despite the overall improvement seen in SF mice, several differences were found between the sub-groups SF1, SF2, and SF3, treated with CACs pre-stimulated with atherosclerotic factors from different donors. Indeed, SF1 mice had the lowest perfusion recovery and more necrotic fingers (compared to other SF groups) but a still higher percentage than SC, while SF2 and more significantly SF3 had the best recovery (compared to SC and even SE mice). In addition, the protein profile of SF1 was closer to SC mice than to other SF ones. As an example, TGFβ1 levels were similar in SF1, even lower, than in SC (Figure 3D).

Remarkably, we found out that patient-1 (SF1) had the worst clinical prognosis among all three donors, and moreover, it was the only diabetic patient. Interestingly, several key enzymes involved in the metabolism of lipids/fatty acids and carbohydrates, among others, were overexpressed only in SF1 mice (Supplementary Table S4). Many of these metabolic alterations have been largely associated with insulin resistance and DM [77,78,79]. It is well known that DM greatly increases the incidence, progression, and severity of CLI [80]. Hyperglycemia affects the development of collateral arteries in response to ischemia and it also promotes down-regulation of proangiogenic factors together with impaired initiation of vascular angiogenesis [80], endothelial dysfunction, or higher inflammatory progression [81,82,83,84]. Furthermore, diabetic patients show dysfunctional CACs [85]. Thus, our data indicated that the secretome of AP arteries from diabetic patients might include factors differentially influencing the regenerative properties of administered cells, explaining the SF1 group results. Future work should focus on evaluating the differential effect of atherosclerotic secretomes over CACs depending on the donor’s prognosis and/or related pathologies.

### 4.4. Modulation of Immune Cell Recruitment by CACs

Proteomic and IHC results highlighted the potential of CACs to modulate immune cell recruitment. Indeed, two days after cell administration, neutrophils were recruited in both SC and SE mice, however, from day 4 onwards, CACs started to regulate such mobilization, reducing the levels of the inflammatory cells in the ischemic limbs [12]. In the long term, untreated mice continued recruiting a high number of immune cells to the ischemic area, while neutrophils and macrophages were significantly less abundant in response to CACs, even pre-stimulated.

The capacity of CACs to modulate neutrophils and macrophages mobilization has already been described [86]. This control over recruitment seems very important since the persistence of pro-inflammatory macrophages or neutrophils correlates with a slower blood flow recovery or worst muscle injury [87,88]. These cells arrive first to the injured area, releasing cytokines, chemo-attractants, or other factors against ischemia in a coordinated manner [89]. In fact, the interaction between both cell types must be tightly regulated to avoid any exacerbated inflammatory response [88,89].

On the other hand, neutrophils and macrophages are also thought to play an important role in the angiogenic/arteriogenesis processes, promoting revascularization and tissue remodeling [90]. Both cells present different phenotypes and functions depending on the physiological or pathological environment. Macrophages can have either a pro-inflammatory (M1) or an anti-inflammatory (M2) phenotype, while neutrophils might differentiate into diverse phenotypes with differential functions as well [91]. Remarkably, the polarization towards M2 might be promoted by different progenitor cells, as suggested [92], besides, CACs could act as alternative M2 macrophages in some cases [92]. M1 subtype (IL-12^high^ and IL-10^low^) express inducible nitric oxide synthase (iNOS) and CD40, and produce TNFα, IL6, or IL1β, while M2 macrophages (IL12^low^ and IL10^high^) express arginase-1 (ARG1) and CD206 and produce, among others, TGFβ or IL10 to facilitate tissue repair [93,94,95,96]. Similarly, pro-inflammatory N1 and anti-inflammatory N2 neutrophils, have been recently associated with cerebral ischemia [97,98], so we do not rule out that neutrophil polarization may also occur in PAD, although, at least to our knowledge, this phenomenon has not been described yet.

According to our results, SE mice (CACs, unstimulated) showed high levels of CD40 and IL1β compared to untreated SC, but also ARG1 and CD68 (M2 associated markers), and therefore, we could not indicate any clear polarization tendency in SE mice. On the contrary, SF mice showed high levels of IL10 and TGFβ and low levels of IL1β, suggesting a possible polarization towards an M2 profile in this group. IL6 was also up-regulated in SF mice, although this cytokine can induce polarization of both, M1 or M2 macrophages in a context-dependent manner [86]. Thus, although CACs and moreover, pre-stimulated CACs might affect the monocyte/macrophage phenotype, further studies should be conducted to validate such a hypothesis. Nevertheless, it seems clear at least, that CACs modulated the presence of MOMA (macrophages) and Lys6G (neutrophils), significantly decreasing their levels in the ischemic tissues.

Overall, CACs promoted revascularization in CLI mice, by enhancing from early stages the maturation of a functional vascular network and, for instance, a higher blood flow recovery in the long term. Of note, CACs did not induce a significant increase of vascular density, compared to similar studies with these and other cells [31], although this could also be dependent on the severity of the CLI model or the strain used [37]. Likewise, co-administration of CACs with other cell types might help to increase perfusion even higher than the one seen by promoting higher arteriogenesis and vessel density, as suggested [24,99]. Nevertheless, our results suggest that vessel sprouting and further pruning might have occurred after CACs administration to enhance such a functional network.

## 5. Conclusions

Based on the changes seen in the long term in CACs-treated mice, their effect on the first days seems crucial, enhancing reperfusion while modulating the participation of immune cells to assist in tissue restoration without producing an exacerbated inflammatory response, reducing cell death and, for instance, the progression of the ischemic damage.

We have provided, to our knowledge, the widest overview of the protein changes taking place in a mouse model of CLI and in response to CACs treatment. While some of the proteins identified might be taken as molecular markers of CLI, our results bring the opportunity to further explore the biological meaning of the molecular changes found here. Indeed, the role of proteins such as MMP9, CD44, or TGFβ in the ischemic process and in response to CACs should be further investigated. Finally, in the current study, we have also shown, for the first time, that pre-stimulation of CACs with atherosclerotic factors not only does not seem to exert a negative impact on these cells but on the contrary, atherosclerotic pre-conditioning also appears to potentiate the regenerative properties of CACs in vivo.

## Figures and Tables

**Table 1:** **Functional classification of protein changes due to ischemia.** Protein changes in ischemic mice (SC, SE, and SF vs. shams) were classified with IPA, highlighting the most probable functions of proteins of interest. Legend: ↑ up-regulated; ↓ down-regulated; ▲ predicted activation increased; ▼ predicted activation decreased.

Categories	Functions (*p*-Value)	Activation z-Score	Molecules	Proteins
Cardiovascular System Development and Function	Vasculature development (1.29E-06)	2.856 ▲	ABI1↑, ADD1↑, ANXA1↑, APOE↑, CASP3↑, CD34↑, COL4A1↑, COMMD1↑, CYBA↑, CYBB↑, EMILIN1↑, F11R↑, FLNA↑, G6PD↑, GIT1↑, GLUL↑, HLA-DQB1↑, HSPB1↑, HSPB6↑, HSPB7↑, HSPG2↑, IGFBP7↑, LAMA4↑, LGALS3↑, LRP1↑, Mcpt4↑, MMP2↑, MMP9↑, NOS3↑, NPC2↑, P2RX4↑, PAK1↑, PLVAP↑, PLXNB2↑, POFUT1↑, PROCR↑, PTGES↑, S100A4↑, SERPINH1↑, SGPL1↑, STAT3↑, THRAP3↑, TKT↑, GTF2I↓, HRAS↓, ICAM1↓, SMOC2↓	47
	Angiogenesis (3.63E-06)	2.855 ▲	ABI1↑, ADD1↑, ANXA1↑, APOE↑, CASP3↑, CD34↑, COL4A1↑, COMMD1↑, CYBA↑, CYBB↑, EMILIN1↑, F11R↑, FLNA↑, G6PD↑, GLUL↑, HLA-DQB1↑, HSPB1↑, HSPB6↑, HSPG2↑, IGFBP7↑, LAMA4↑, LGALS3↑, LRP1↑, Mcpt4↑, MMP2↑, MMP9↑, NOS3↑, PAK1↑, PLVAP↑, POFUT1↑, PROCR↑, PTGES↑, S100A4↑, SERPINH1↑, SGPL1↑, STAT3↑, THRAP3↑, TKT↑, GTF2I↓, HRAS↓, ICAM1↓, SMOC2↓	42
	Vasculogenesis (1.21E-05)	2.575 ▲	ABI1↑, ANXA1↑, APOE↑, CASP3↑, CD34↑, COL4A1↑, COMMD1↑, CYBA↑, CYBB↑, F11R↑, FLNA↑, G6PD↑, GLUL↑, HLA-DQB1↑, HSPG2↑, IGFBP7↑, LGALS3↑, LRP1↑, Mcpt4↑, MMP2↑, MMP9↑, NOS3↑, PAK1↑, PLVAP↑, POFUT1↑, PROCR↑, S100A4↑, SERPINH1↑, SGPL1↑, STAT3↑, THRAP3↑, TKT↑, HRAS↓, ICAM1↓, SMOC2↓	35
Cell Death and Survival	Apoptosis (1.56E-06)	−2.602 ▼	ACACA↑, ACLY↑, ALDH3A1↑, ANXA1↑, APOE↑, ARF4↑, ARL6IP1↑, ASAH1↑, ASNS↑, BASP1↑, CASP3↑, CASP6↑, COL4A1↑, CTSZ↑, CYBA↑, CYBB↑, CYP2F1↑, DDX58↑, DPM3↑, DTYMK↑, ELMO1↑, EMILIN2↑, ERC1↑, Ewsr1↑, FAH↑, FASN↑, FLNA↑, G6PD↑, GMDS↑, HLA-DQB1↑, HRAS↓, HSPB1↑, HSPB6↑, HSPG2↑, IGFBP7↑, ILF3↑, IQGAP2↑, IRF2BP2↑, Irgm1↑, LAMA4↑, LGALS3↑, LGALS7/LGALS7B↑, LGMN↑, LIMS1↑, LRP1↑, LSP1↑, Mcpt4↑, MMP2↑, MMP9↑, Mt2↑, MTA2↑, NELFB↑, NFKB2↑, NOS3↑, P2RX4↑, PAK1↑, PEX11B↑, PPP1R9B↑, PRKAR2B↑, PRMT1↑, PROCR↑, PRTN3↑, PTGES↑, RAB32↑, S100A10↑, S100A4↑, SCD↑, SEL1L↑, SERPINB1↑, SERPINH1↑, SET↑, SGPL1↑, SIRPA↑, SLC25A10↑, SLC25A5↑, SNX1↑, SRI↑, SRSF2↑, STAT3↑, SWAP70↑, TARDBP↑, TRIM28↑, UBA2↑, WDR5↑, XRCC5↑, ICAM1↓, IFIT2↓, MAPK12↓, NQO1↓, PAFAH1B3↓, TIGAR↓	91
	Cell survival (7.27E-04)	3.789 ▲	ACACA↑, ACLY↑, APOE↑, ASAH1↑, ASNS↑, CASP3↑, CASP6↑, CYBA↑, CYBB↑, EMILIN2↑, FASN↑, FLNA↑, GALK2↑, GIT1↑, GPNMB↑, HLA-A↑, HNRNPU↑, HSPB1↑, HSPB6↑, IGFBP7↑, LGALS3↑, LGALS7/LGALS7B↑, LIMS1↑, LRP1↑, MCFD2↑, MGST1↑, MMP9↑, Mt2↑, NFKB2↑, NOS3↑, PAK1↑, POSTN↑, PRMT1↑, PRTN3↑, PSME3↑, S100A4↑, SCD↑, SEL1L↑, SET↑, SGPL1↑, SHMT2↑, STAT3↑, TARDBP↑, THRSP↑, TRIM28↑, XRCC5↑, HRAS↓, ICAM1↓, MAPK12↓, SMC1A↓	50
Cellular Movement	Cell movement (1.03E-09)	4.746 ▲	ABI1↑, ACACA↑, ADD1↑, ANXA1↑, APBB1IP↑, APOE↑, ARF4↑, ARFGAP3↑, ARPC1B↑, ASNS↑, BIN2↑, CAP1↑, CD34↑, CD93↑, CLIC1↑, COL4A1↑, COMMD5↑, CORO1C↑, CTSZ↑, CYBB↑, DDX58↑, ELMO1↑, EMILIN2↑, ERC1↑, EVL↑, F11R↑, FABP5↑, FASN↑, FHL3↑, FLNA↑, FLNC↑, FSCN1↑, G6PD↑, GIT1↑, GIT2↑, GLUL↑, GPNMB↑, HLA-A↑, HNRNPA2B1↑, HSPB1↑, HSPG2↑, IFITM3↑, IGFBP7↑, ILF3↑, Irgm1↑, LAMA4↑, LCP1↑, LGALS3↑, LGMN↑, LIMS1↑, LIPE↑, LRP1↑, LSP1↑, Mcpt4↑, MMP2↑, MMP9↑, MRC2↑, Mt2↑, NAAA↑, NDUFAF3↑, NFKB2↑, NOS3↑, P2RX4↑, PAK1↑, PEX11B↑, PLXNB2↑, POSTN↑, PPP1R9B↑, PRMT1↑, PROCR↑, PRTN3↑, PTGES↑, RGS10↑, S100A10↑, S100A4↑, SERPINB1↑, SERPINH1↑, SGPL1↑, SIRPA↑, STAT3↑, SWAP70↑, TARDBP↑, TKT↑, GTF2I↓, HRAS↓, ICAM1↓, IFIT2↓, MAPK12↓, NNMT↓, NQO1↓, SMOC2↓, Tpm1↓, TPM3↓	93
	Cell Migration (1.17E-07)	4.562 ▲	ACACA↑, ADD1↑, ANXA1↑, APBB1IP↑, APOE↑, ARF4↑, ARFGAP3↑, ASNS↑, CAP1↑, CD34↑, CD93↑, COL4A1↑, COMMD5↑, CORO1C↑, CTSZ↑, CYBB↑, DDX58↑, ELMO1↑, EMILIN2↑, ERC1↑, EVL↑, F11R↑, FABP5↑, FASN↑, FLNA↑, FLNC↑, FSCN1↑, G6PD↑, GIT1↑, GIT2↑, GLUL↑, GPNMB↑, HLA-A↑, HNRNPA2B1↑, HSPB1↑, IFITM3↑, IGFBP7↑, ILF3↑, Irgm1↑, LAMA4↑, LCP1↑, LGALS3↑, LGMN↑, LRP1↑, LSP1↑, Mcpt4↑, MMP2↑, MMP9↑, MRC2↑, NAAA↑, NFKB2↑, NOS3↑, P2RX4↑, PAK1↑, PEX11B↑, PLXNB2↑, POSTN↑, PPP1R9B↑, PRMT1↑, PROCR↑, PRTN3↑, PTGES↑, S100A10↑, S100A4↑, SERPINB1↑, SGPL1↑, SIRPA↑, STAT3↑, SWAP70↑, TARDBP↑, HRAS↓, ICAM1↓, IFIT2↓, MAPK12↓, NDUFAF3↓, NNMT↓, NQO1↓, SMOC2↓, Tpm1↓, TPM3↓	80
	Chemotaxis (5.15E-07)	2.773 ▲	ANXA1↑, APOE↑, BIN2↑, CYBB↑, ELMO1↑, GIT1↑, GIT2↑, LCP1↑, LGALS3↑, LGMN↑, LRP1↑, LSP1↑, MMP2↑, MMP9↑, Mt2↑, NFKB2↑, NOS3↑, P2RX4↑, PAK1↑, PRTN3↑, PTGES↑, RGS10↑, S100A4↑, SERPINB1↑, SERPINH1↑, SIRPA↑, STAT3↑, SWAP70↑ HRAS↓, ICAM1↓	30

**Table 2:** **Classification of proteins differentially expressed in CACs-treated mice (SE, SF) on day 21.** Protein classification was done with IPA, based on the effect of CACs treatment vs. SC levels. The names of protein variations in SE and SF vs. SC on day 21 are shown. Legend: ↑ up-regulated; ↓ down-regulated; ▲ predicted activation increased; ▼ predicted activation decreased; ▬ unknown predicted activation.

SE/SC	SF/SC	Proteins	Functions
↑	↑	LSMEM1	Necrosis ▲ Apoptosis ▲ Atherosclerosis ▬ Neovascularization ▬ Angiogenesis ▼ Vasculature Development ▼ Endothelial cell development ▲ Endothelial cell Proliferation▲
↓	↓	AACS, ALDH3A1, ATP2B4, BPHL, BTF3L4, CASQ2, CAV1, CAV3, CD99, CDC5L, CILP, CKAP5, COX6A1, CREG1, FAU, FHL1, FLG, GAMT, GCDH, GM8210, GPC1, GPNMB, HDGFL2, HSPB6, HSPB7, IFI30, IRF2BPL, KLHL41, LAMP2, LGALS3, MAN2B2, MAP3K20, MCAM, MRPL12, MRPS5, MRPS27, MTCH1, MYL4, MYL6B, NES, PLOD2, PRKG1, RBBP6, RPLP1, RPL13, RPL17, RPL21, RPL35, RPL37A, RPS17, S100A11, SCD1, SPARC, SEC16A, SPRR1A, SLMAP, SERPINF1, SERPINB6A, SERF2, SNRPD2, STMN1, TGTP1, TNC, TMEM167, TPP1, XIRP1	
↑	-	ABCA1, APAF1, APBB1IP, APOBR, ARG2, ARL6IP1, ASS1, BC017643, CAPZA1, CAR13, CASP8, CD14, CYBB, DCAKD, DDX39, DHFR, DR1, DTYMK, ELANE, ELMO1, EMB, EMILIN1, EMILIN2, GCN1L1, GFPT1, GIT2, H2AFY, HMOX1, HVM22, IFIH1, IFIT1, IFIT2, IGHV5-4, IGJ, IGKV12-44, IGKV12-47, IGKV14-111, IL1RN, IQGAP2, ITGB2, LGALS7, LGALS9, LOC433053, LSP1, MOGS, MPO, MSR1, NAPSA, NCF1, NCF2, NCKAP1L, PLBD1, PLIN2, PRPF40A, P2RX4, PRTN3, PSTPIP1, PTGES, PTPN1, RETNLG, ROD1, SAA4, SDF2L1, SEL1L, SKAP2, SNX18, SPCS3, SLC9A3R1, SRC, STFA1, STS, SUN2, SUPT16H, TAP1, TBL2, VAV1, WASF2	Apoptosis ▲ Atherosclerosis ▲ Angiogenesis Vasculature Development ▬ Vasculogenesis ▬ Atherogenesis ▬ Activation of cells ▲ Cell movement ▲ Immune response of cells ▲
↓	-	ABHD5, ACADS, ACAT2, ACLY, ALDH2, ALDH1A7, ALDH1L1, ALDH4A1, CAR5B, CES1D, CES1F, COMP, COX8B, 4931406C07RIK, CRABP1, CRYAB, CSAD, CSRP3, CYP2E1, DNAJA4, ECHDC1, EPHX2, FABP4, FAH, FASN, GADD45GIP1, G3BP2, GLUL, GNAI1, GSTA3, GSTZ1, HDHD3, KLHL40, KRT10, LIPE, ME1, MGLL, MRPL11, MRPL41, MRPL53, NRAP, PCBD1, PCX, PDLIM1, PTGES3L, PTMS, PLIN1, RBP7, SEPT4, SSC5D, SLC25A1, THRSP, THBS4, TST, YAP1	
-	↑	None	Necrosis ▲ Apoptosis ▲ PAD ▬ Diabetes mellitus ▬ Cell Activation ▼ Cell Attachment ▼ Cell movement ▼ Cell Migration ▼ Cell Outgrowth ▼ Lipid Accumulation ▲ Quantity of IFN in blood ▲
-	↓	ACOT9, ACOX1, ACP2, ACTB, ACTN1, ACTN2, ACTN4, ADIPOQ, ADPRHL2, AIF1, ALDH16A1, ANKFY1, ANO6, AMDHD2, AMY1, APCS, AP1M1, AP2M1, APOBEC2, APOD, APOE, ARHGDIB, ARPIN, ARPC5, ART3, BASP1, BGN, BIN2, B2M, CACNG1, CD44, CDH13, CFH, CKAP4, CLTC, CNN2, COA7, COL1A2, COL12A1, COL14A1, COLGALT1, COPA, COPB1, COPE, COPG1, COPG2, CORO1C, COTL1, CSK, CTSA, CTSC, CTSD, CTSH, CTSZ, DCLK1, DCUN1D2, DDOST, DDX5, DHX15, DNM3, DNPEP, DYNC1H1, EEF1AKMT1, EEF1B2, EFEMP1, EIF4A3, EIF3E, EIF3L, EFHD2, EHD2, EHD4, ERGIC3, ERO1LB, ERP29, ETF1, ETFDH, EVL, FKBP15, FN1, GC, GLA, GM5571, GMFB, GMPR2, GSTT1, GSTT3, GUSB, H2-AA, H2-AB1, H2AFV, HAL, H2-D1, H2-EB1, HEXB, HIST1H4A, HIST1H2BB, HNRNPC, HPRT, HRG, HSPB2, HYPK, IFI204, IFI211, IFI44L, IGHG, IGHG3, IGHV2-3, IIGP1B, IQGAP1, IRGM1, ISG15, ITGA6, ITGB4, JPT1, LAMTOR5, LCP1, LGALS3BP, LGMN, LIPA, LMNA, LRRC59, LXN, MAN2A1, MAN2B1, MARCKSL1, MBL1, ME2, MFF, MMP9, MPZ, MYH7, MYL6, MYO1C, NAA15, NAA20, NAP1L1, NCAM1, NDUFB6, NGLY1, NGP, ORM2, OST4, PACSIN3, PAPSS2, PCNA, PDXK, PEA15A, PIGS, PMP2, POSTN, PPIC, PP1R14B, PPP1R14C, PRCP, PRDX4, PRX, PSMB10, PSMC1, PTRH2, PYCARD, RAB12, RACK1, RCC2, RCN3, RENBP, RNF213, RPA3, RPL3, RPL4, RPL5, RPL6, RPL7, RPL8, RPL9, RPL10A, RPL11, RPL12, RPL13A, RPL14, RPL15, RPL7A, RPL18, RPL22, RPL23, RPL23A, RPL24, RPL26, RPL27, RPL27A, RPL28, RPL36, RPLP0, RPLP2, RPL32P, RPL10-PS1, RPL19-PS11, RPL34-PS1, RPL36A-PS3, RPS2, RPS3, RPS5, RPS7, RPS8, RPS9, RPS10, RPS11, RPS14, RPS18, RPS19, RPS20, RPS21, RPS25, RPS28, RPS29, RPS15A, RPSA, RPS3A1, RPS2-PS13, RPS13-PS1, RPS26-PS1, RPS6-PS4, RPS16-PS2, RPS4X, RTCB, S100A4, SCAMP1, SCAMP3, SEPT5, SF3B5, SF3B6, SFN, SH3BGRL3, SIRPA, SLN, SNRPF, SH3BGRL, SNRPD3, SPATA7, SRSF2, SRSF1, SRSF5, SSR4, STARD7, STEAP3, STOM, SUB1, SULT1A1, SYNC, SYNGR2, TANGO2, TAPT1, THY1, TNNC1, TNNT3, TMED9, TM9SF3, TPD52, TRIM72, TUBA1A, TUBB2A, TUBB5, TWF2, UCHL1, UFM1, UFSP2, UPP1, VAT1, VPS29, WDR82, ZBP1	
↑	↓	ADGRE1, ALDH1B1, ALOX5AP, AOAH, AP3D1, ARG1, CAD, C5AR1, CD48, CD68, CD180, CEP170, CPT1A, C1QTNF3, C1S1, CTSG, CTSS, CYBA, DDX58, DNAJB11, DNASE2A, FYB, GALK2, GSDMD, H13, HEXA, HK3, IFI44, ITGAM, KDELR2, LBR, MAGOHB, MFSD1, MPEG1, MYL12B, NCF4, OLFM4, PARVG, PHF11D, PLA2G15, PLD4, PLEK, PRPF8, PRRC2A, PTPN6, PTPRC, RAB32, S100A8, SEC11A, SEC61A1, SP100, SRGAP2, STAT1, STAT2, SYK, TAPBP, TAP2, TMED3, TOR3A, TOP2A, STFA3, UGT1A7C, U2SURP	Angiogenesis ▲ Diabetes mellitus ▬ Cell Activation ▲ Cells movement ▲ Immune response of cells ▲ Cell Degranulation ▲
↓	↑	None	

## Data Availability

All the data supporting the findings of this study have been provided within the article, together with online Supplementary files. Also, proteomic results have been deposited to the ProteomeXchange Consortium via the PRIDE partner repository (PXD024132).

## Supplementary Materials

The following materials are available online at https://www.mdpi.com/article/10.3390/biomedicines9091147/s1: Supplementary Materials and Methods; Figure S1: Evaluation of ischemic symptoms within time; Figure S2: Human CACs detection and vascular density; Figure S3: Volcano plot, Figure S4: Cytokine expression arrays, Supplementary Materials and Methods, Table S1: Functional scoring related to ischemia, Table S2: Quantitative analysis of proteins differentially expressed (vs. SH), Table S3: Functional classification of proteins differentially expressed due to administration of AP-prestimulated CACs, Table S4: Proteins differentially expressed in SF vs. SE related to DM.
