# Supplementary material for: Long Term Response to Circulating Angiogenic Cells, Unstimulated or Atherosclerotic Pre-Conditioned, in Critical Limb Ischemic Mice
Source: Biomedicines. 2021 Sep 3;9(9):1147. doi: 10.3390/biomedicines9091147 (PMC8469527; doi:10.3390/biomedicines9091147)
Supplement: Supplementary file 1 [file biomedicines-09-01147-s001.zip › biomedicines-1350445-supplementary.pdf]

biomedicines

MDPI

SUPPLEMENTAL MATERIALS

Long Term Response to Circulating Angiogenic Cells, Unstimulated or Atherosclerotic Pre-conditioned, in Critical Limb Ischemic Mice

Lucía Beltrán-Camacho [1,2], Margarita Jiménez-Palomares [1,2], Ismael Sanchez-Gomar [1,2], Antonio Rosal-Vela [1,2], Marta Rojas-Torres [1,2], Sara Eslava-Alcon [1,2], Jose Angel Alonso-Piñero [1,2], Almudena González-Rovira [1,2], Mª Jesús Extremera-García [1,2], Rosario Conejero [3], Esther Doiz [3], Manuel Rodriguez-Piñero [3], Martin R. Larsen [4] and Mª Carmen Duran-Ruiz [1,2]

1. Biomedicine, Biotechnology, and Public Health Department, Cadiz University, 11002 Cadiz, Spain, lucia.beltrancamacho@alum.uca.es (L.B-C.); margarita.jimenezpalomares@gm.uca.es (M.J-P.); ismael.sanchez@uca.es (I.S-G.); antonio.rosal@uca.es (A.R-V.); marta.rojas@uca.es (M.R-T.); sara.eslava@gm.uca.es (S.E-A.); joseangel.alonsopi@alum.uca.es (J.A.A-P.); almudena.gonzalez@uca.es (A.G-R.); maria.jesus.extremera.garcia@gmail.com (M.J.E-G.).
2. Institute of Research and Innovation in Biomedical Sciences of Cádiz (INiBICA), 11009 Cádiz, Spain
3. Angiology & Vascular Surgery Unit, Hospital Universitario Puerta del Mar, 11009 Cádiz, Spain, rosarioconejero@gmail.com (R.C.); edoiz@comcadiz.com (E.D.); manuel.rodriguez.pinero.sspa@juntadeandalucia.es (M.R-P.).
4. Department of Biochemistry and Molecular Biology, University of Southern Denmark, 5230 Odense, Denmark, mrl@bmb.sdu.dk (M.R.L).

* Correspondence: maricarmen.duran@gm.uca.es (M.C.D-R.)

Supplementary Materials and Methods:

Animals.

Species	Vendor or Source	Background Strain	Sex	Persistent ID / URL
Mus musculus	Charles River Laboratories	Balb-C Nude CAnN.Cg-Foxn1nu/Crl	Female	https://www.criver.com/

Antibodies.

Target antigen	Vendor or Source	Catalog #	Working concentration (µg/ml)	Lot #
CD31-FITC	Biolegend	303103	8	B224876
CD34-APC	Biolegend	343607	2	B223919
CD45-PBlue	Biolegend	368539	4	B264395
CD90-PE	Biolegend	328109	2	B236755
CD73-FITC	Biolegend	344015	16	B224217
CD105-FITC	Biolegend	323203	16	B225651
CD309-PE	Biolegend	359903	8	B245460
CD133-PE	Miltenyi Biotec	130-098-826	1:25 concentration not available	5140404153
CD146-PE	Biolegend	361005	4	B264161
CD14-PB	Biolegend	367121	8	B271628
UEA-1-FITC	Sigma-Aldrich	L9006	3	072M4026V
DiI-ac-LDL	Biomedal	BT-902	0.6	
α-Actin smooth muscle	Sigma	A5228	4	029M4807V
CD31	Abcam	AB32457	2	GR3200348-2
MOMA-2	Sigma-Aldrich	MAB1852	1	3026751
Ly-6G	Biolegend	127601	1	B265458

Alexa Fluor 488 anti-mouse IgG	Thermo Fisher	A10667	4	1962792
Alexa Fluor 555 anti-mouse IgG	Thermo Fisher	A21422	4	1837985
Alexa Fluor 488 anti-rabbit IgG	Thermo Fisher	A11008	4	1832425
Alexa Fluor 555 anti-rat IgG	Thermo Fisher	A21434	4	1907302

Cultured Cells.

Name	Vendor or Source	Sex (F, M, or unknown)
Endothelial progenitor cells (EPCs)	Peripheral blood of healthy donors	Unknown

Atherosclerotic samples.

Donor	Age	Sex (F, M, or unknown)	Smoker	DM2	Dislipidemia	Hypertension
Patient-1	70	M	Yes	Yes	Yes	Yes
Patient-2	64	M	Yes	No	Yes	Yes
Patient-3	Unknown	Unknown	Yes	No	Yes	Yes

Primers.

Description	Sequence	Vendor or Source
Primers forward	5′-GGTGAAACCCCGTCTCTACT-3′	Metabion
Primers reverse	5′-GGTTCAAGCGATTCTCCTGC-3′	Metabion
Hydrolysis FAM label probe	5′-(6-FAM)-CGCCCGGCTAATTTTTGTAT-(BHQ-1)-3′	Metabion

Data & Code Availability.

Description	Source / Repository	Persistent ID / URL
MS data with identifier number PXD024132	ProteomeXchange	http://www.proteomexchange.org/

Other.

Description	Source / Repository
Quick-DNA™ Midiprep Plus Kit (D4075)	Zymo Research
Proteinase K (D3001-2-B)	Zymo Research
TaqMan Universal Master Mix II (4440043)	Thermo-Fisher
Mouse Cytokine Antibody Array C3 (AAM-CYT-3)	RayBiotech
TGFβ1 ELISA (CSB-E04726m)	Cusabio

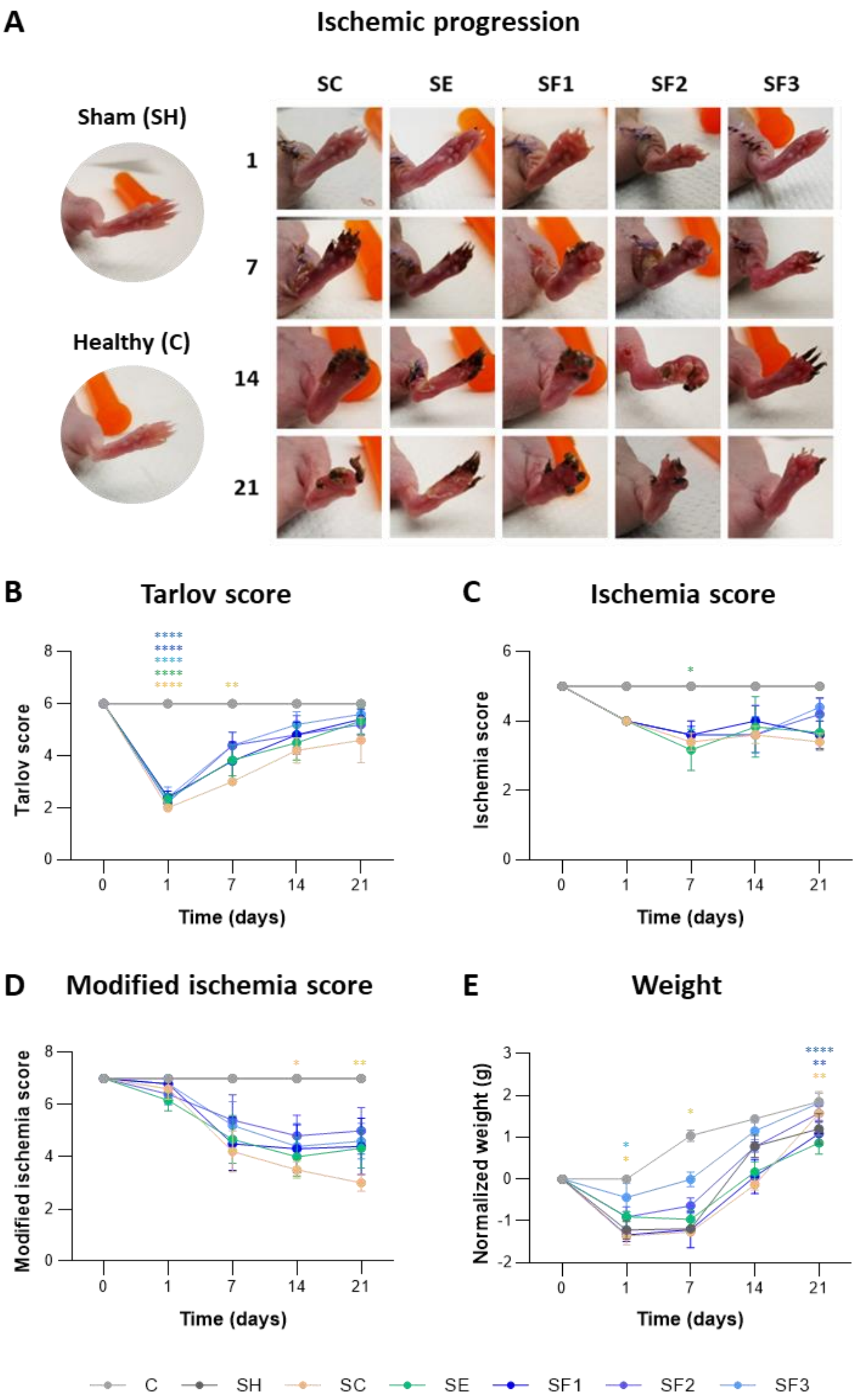

Figure S1. Evaluation of ischemic symptoms within time. (**A**) Representative images of the evolution of ischemic symptoms in mice. (**B**) Motility changes (Tarlov score) and Ischemic changes according to (**C**) Ischemia scores and (**D**) Modified Ischemia scores, all of them described in supplementary table S2. (**E**) Weight changes. Groups analyzed: Healthy controls (C, n:2); Sham, surgery controls (SH, n:4); ischemic mice, untreated (SC, n:5): ischemic mice with unstimulated CACs (SE, n:6) or pre-stimulated with atherosclerotic factors (SF: n:15: SF1, n:5; SF2, n:5; SF3, n:5). Data were presented as mean ± SEM and significant differences were seen by two-way ANOVA and Tukey post-hoc. Statistical differences shown were calculated using the SH group as baseline per day.

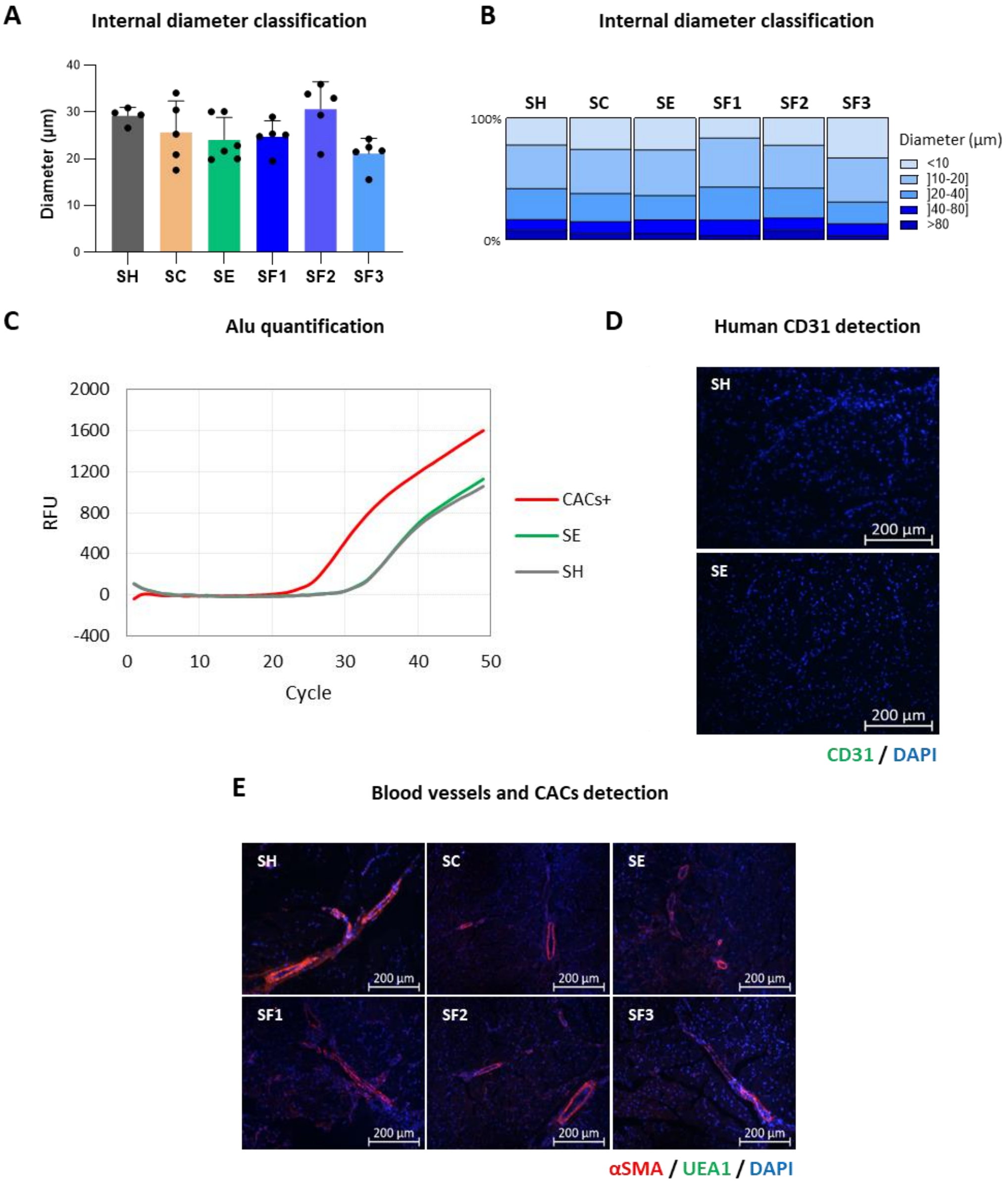

Figure S2. Human CACs detection and vascular density. A) Diameter size (µm). **B)** Vessel classification based on abundance (percentage) of different ranges of internal lumen diameter (µm). **C)** Representative results of the amplification of human-specific Alu sequences by qPCR, with the SH group as negative control. A positive result (CACs + , red) obtained from mice sacrificed at early days[1] has also been included. **D)** Representative IHC images using anti-hCD31 (green) and DAPI (blue). **E)** Representative IHC images taken to measure vascular density and diameter size with anti-mouse smooth muscle α-actin (red), and human cells using UEA1 (green) and DAPI (blue). Groups analyzed: SH: Sham, surgery controls (n:4), SC: Ischemic mice, no cell treatment (n:5), ischemic mice treated with unstimulated CACs (SE, n:6) or with pre-stimulated CACs (SF, n:15: SF1, n:5; SF2, n:5; SF3, n:5). Data were presented as mean ± SD and significant differences were seen by Kruskal-Wallis and Dunn's multiple comparisons test.

Supplementary figure S3

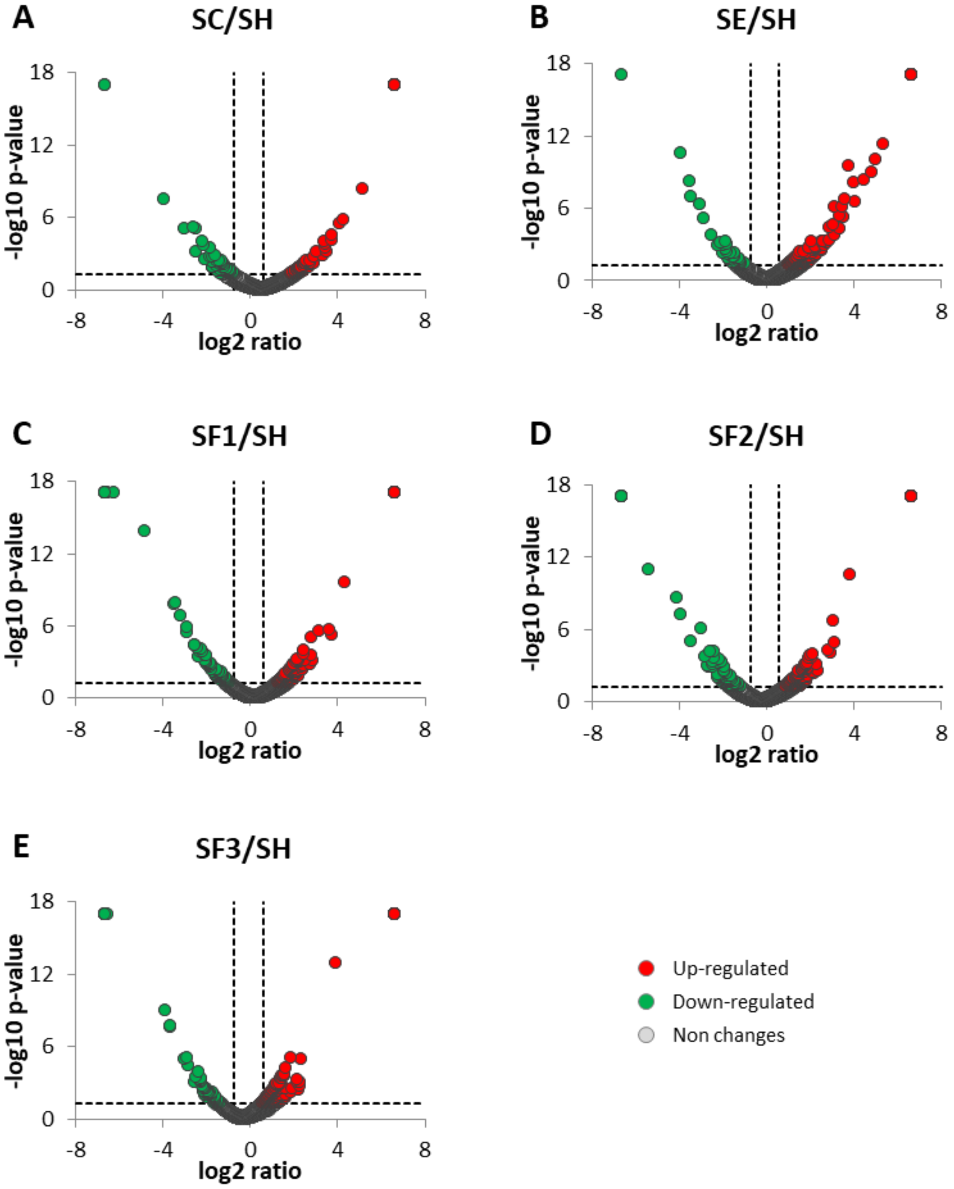

Figure S3. Volcano plots. Volcano plots obtained after label free quantification, comparing protein levels in **A)** SE, **B)** SC, **C)** SF1, **D)** SF2 and **E)** SF3 vs SH. Cut-off limits: p-value < 0.05, ratios for up-regulated (> 1.5) and down-regulated (< 0.6) proteins. Groups analyzed: SH: Sham, surgery controls (n:4), SC: Ischemic mice, no cell treatment (n:5), ischemic mice treated with unstimulated CACs (SE, n:6) or with pre-stimulated CACs (SF, n:15: SF1, n:5; SF2, n:5; SF3, n:5).

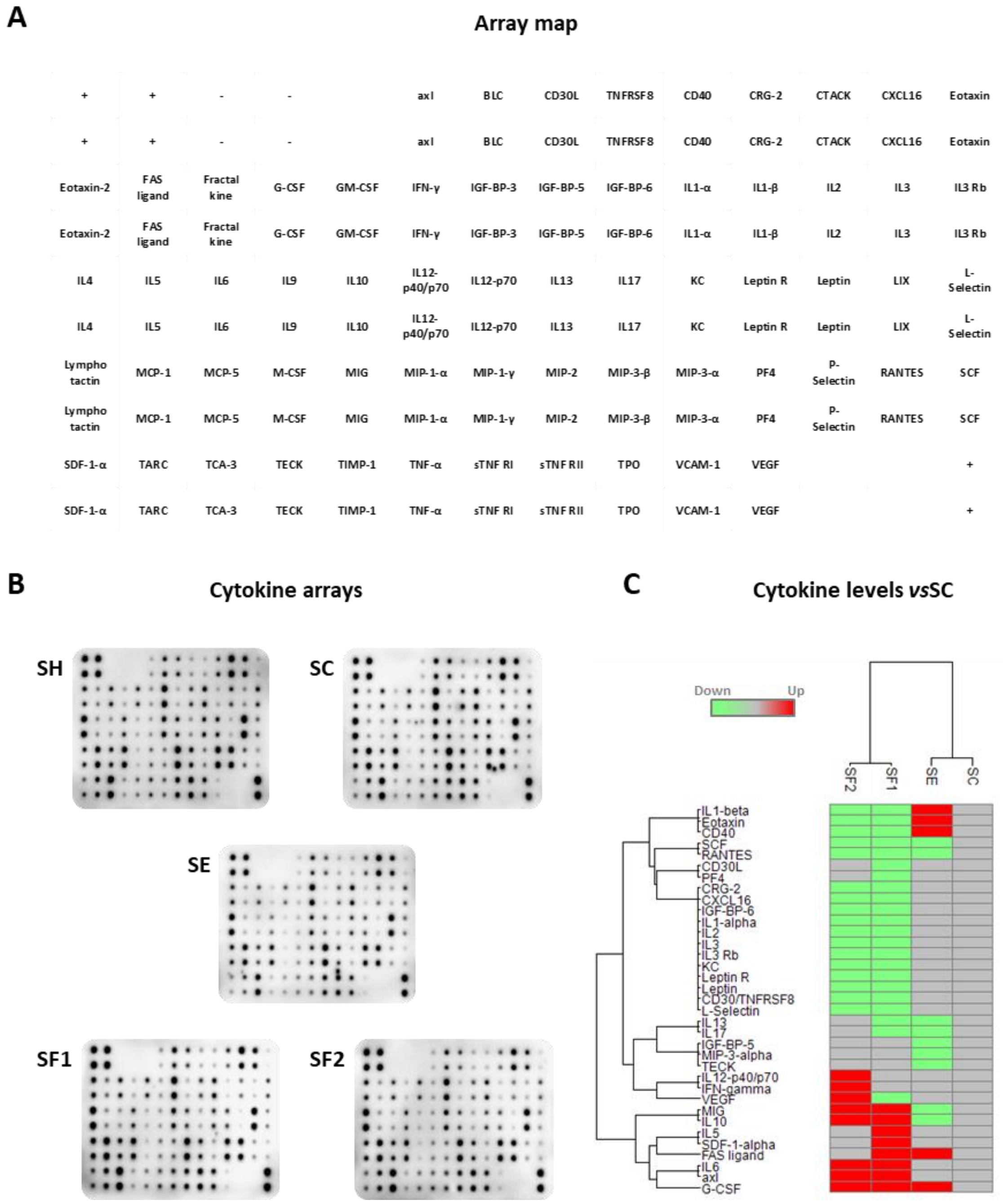

Figure S4 Cytokine expression arrays. (**A**) Map of Mouse Cytokine Antibody Array C3 (RayBiotech; AAM-CYT-3) (**B**) Cytokine arrays images acquired with ChemiDoc Touch System (Biorad) (**C**) Hierarchical cluster with differential levels patterns detected in cytokines arrays SE, SF1 and SF2 *vs* SC group. Groups analyzed: SH: Sham, surgery controls (n:4), SC: Ischemic mice, no cell treatment (n:5), ischemic mice treated with unstimulated CACs (SE, n:6) or with pre-stimulated CACs (SF, n:15: SF1, n:5; SF2, n:5).

Table S1. Functional scoring related to ischemia. The table includes (from left to right): Tarlov score (evaluating motility, leg movement and difficulty walking), ischemia score (ischemic symptoms, advance of the characteristics along the leg) and modified ischemia score (scores adapted to mice studies, evaluating the advance of the characteristics along the leg) [2, 3].

Score	Tarlov	Ischemia	Modified ischemia
**0**	No movement	Auto-amputation > half lower limb	Auto-amputation of leg

**1**	Barely perceptible movement, non-weight bearing	Gangrenous tissue > half foot	Leg necrosis
**2**	Frequent movement, non-weight bearing	Gangrenous tissue < half foot, with lower limb muscle necrosis	Foot necrosis
**3**	Support weight, partial weight beaing	Gangrenous tissue < half foot, without lower limb muscle necrosis	Discoloration of > two toes
**4**	Walks with mild déficit	Pale foot or gait abnormalities	Discoloration of one toe
**5**	Normal but slow waking	Normal	Discoloration of > two nails
**6**	Full and fast walking		Discoloration of one nail
**7**			No necrosis

Table S2. Quantitative analysis of proteins differentially expressed (vs SH). The table includes (from left to right): Uniprot accession number, protein description, abundance ratio (considering up-regulated ratio > 1.5 and down-regulated ratio < 0.6), p-value (considering P-value < 0.05 as differentially significant), number of amino acids (AAs), number of peptide sequences (PSMs), number of unique peptides and coverage percentage.

**Accession**	**Description**	**Abundance ratio**					**P-value**					**#AAs**	**#PSMs**	**#Unique Peptides**	**Coverage [%]**
		**SC/SH**	**SE/SH**	**SF1/SH**	**SF2/SH**	**SF3/SH**	**SC/SH**	**SE/SH**	**SF1/SH**	**SF2/SH**	**SF3/SH**				
Q9CQI6	Coactosin-like protein	4.8530	2.7550	2.8920	2.6420	2.0080	0.0151	0.0149	0.0262	0.0090	0.0077	142	445	9	61
P70333	Heterogeneous nuclear ribonucleoprotein H2	4.6310	2.5320	2.9420	2.3840	2.0090	0.0231	0.0313	0.0159	0.0110	0.0048	449	730	7	47
P40124	Adenylyl cyclase-associated protein 1	4.8640	2.4780	2.7790	2.5480	1.9340	0.0135	0.0168	0.0205	0.0136	0.0066	474	1637	27	64
Q6R891	Neurabin-2	10.9290	6.1790	7.2810	7.1010	4.8940	0.0004	0.0012	0.0008	0.0001	0.0019	817	60	2	2
P40142	Transketolase	4.0750	2.3190	3.7140	3.0910	2.3270	0.0313	0.0203	0.0028	0.0023	0.0012	623	3318	41	79
P97352	Protein S100-A13	5.2420	3.4960	3.5990	3.4500	2.4470	0.0166	0.0051	0.0111	0.0009	0.0020	98	231	5	46
P14602	Heat shock protein beta-1	4.3970	2.8980	3.9530	3.0990	2.6890	0.0221	0.0081	0.0017	0.0023	0.0003	209	776	11	55
Q8VHX6	Filamin-C	4.6460	2.7710	2.9340	2.3920	2.0410	0.0170	0.0063	0.0145	0.0120	0.0041	2726	6468	126	66
Q6IRU2	Tropomyosin alpha-4 chain	5.2290	3.0340	3.3770	3.1020	1.9490	0.0137	0.0098	0.0105	0.0025	0.0140	248	811	13	55
O89017	Legumain	10.6690	4.8140	5.1510	3.6250	2.2230	0.0002	0.0021	0.0022	0.0008	0.0292	435	226	7	21
P13516	Acyl-CoA desaturase 1	7.4910	3.1950	8.9460	3.6250	5.0430	0.0021	0.0331	0.0000	0.0020	0.0000	355	183	8	30
P15105	Glutamine synthetase	4.7380	2.2060	4.5070	3.5840	2.6030	0.0154	0.0275	0.0006	0.0008	0.0004	373	2095	25	68
Q61553	Fascin	4.6560	2.2960	2.9670	2.7640	2.0160	0.0168	0.0273	0.0135	0.0084	0.0046	493	798	21	52
Q9D154	Leukocyte elastase inhibitor A	4.4800	2.1650	2.4570	2.1230	1.5830	0.0202	0.0308	0.0421	0.0239	0.0301	379	1610	29	63
P19324	Serpin H1	8.2440	2.9650	4.7580	4.3050	3.0030	0.0008	0.0096	0.0008	0.0001	0.0002	417	760	16	53
Q91V92	ATP-citrate synthase	5.5960	2.1500	5.4820	4.1150	3.0850	0.0065	0.0321	0.0001	0.0003	0.0001	1091	5085	69	74
P16110	Galectin-3	10.6440	4.2240	4.7360	3.8400	2.4950	0.0001	0.0006	0.0009	0.0002	0.0011	264	497	12	38
P07091	Protein S100-A4	5.8090	3.0240	3.4300	3.3700	2.8400	0.0073	0.0096	0.0120	0.0013	0.0003	101	179	5	43

P47739	Aldehyde dehydrogenase, dimeric NADP-preferring	6.6250	2.4390	3.7580	8.1650	3.0190	0.0039	0.0387	0.0059	0.0000	0.0002	453	473	21	64
Q9WV32	Actin-related protein 2/3 complex subunit 1B	5.1030	2.8600	3.2270	3.0670	2.1840	0.0125	0.0181	0.0116	0.0022	0.0022	372	757	19	60
Q9ERN0	Secretory carrier-associated membrane protein 2	5.3590	3.6910	3.8570	3.0300	2.2380	0.0144	0.0196	0.0189	0.0188	0.0499	329	12	2	4
P61965	WD repeat-containing protein 5	19.1370	15.9410	20.2310	14.3320	15.1070	0.0000	0.0000	0.0000	0.0000	0.0000	334	4	2	10
O88569	Heterogeneous nuclear ribonucleoproteins A2/B1	3.7550	2.2720	2.5580	2.3260	1.7560	0.0448	0.0231	0.0336	0.0142	0.0142	353	1562	19	50
Q9WV54	Acid ceramidase	4.6840	2.4190	2.6720	2.5120	2.0680	0.0213	0.0371	0.0444	0.0105	0.0082	394	430	15	46
P29391	Ferritin light chain 1	5.4960	2.6890	3.4820	2.8780	1.8220	0.0072	0.0103	0.0045	0.0057	0.0106	183	773	18	90
P19096	Fatty acid synthase	6.0070	2.3900	6.9580	3.5000	3.6340	0.0044	0.0168	0.0000	0.0009	0.0000	2504	15771	150	78
P10107	Annexin A1	3.8420	2.4300	2.5220	2.2790	1.6350	0.0406	0.0152	0.0364	0.0160	0.0240	346	1800	32	76
Q9ES30	Complement C1q tumor necrosis factor-related protein 3	35.4010	21.6570	13.5680	7.7800	2.7030	0.0000	0.0000	0.0000	0.0001	0.0263	246	26	3	13
P49312	Heterogeneous nuclear ribonucleoprotein A1	3.8450	2.1080	2.3930	2.1680	1.7220	0.0405	0.0423	0.0486	0.0168	0.0164	320	1305	18	55
Q61233	Plastin-2	4.9710	2.7370	2.5980	2.4170	1.5500	0.0121	0.0066	0.0307	0.0114	0.0348	627	1840	42	88
Q05816	Fatty acid-binding protein 5	5.8390	2.9350	3.9250	3.2640	2.2820	0.0052	0.0041	0.0018	0.0015	0.0015	135	1111	11	73
Q9R1C7	Pre-mRNA-processing factor 40 homolog A	4.3660	10.1900	3.8770	2.8650	2.4830	0.0598	0.0000	0.0287	0.0335	0.0495	953	8	3	3
Q00612	Glucose-6-phosphate 1-dehydrogenase X	2.9900	2.0400	2.7430	2.1800	1.7720	0.1097	0.0463	0.0222	0.0273	0.0132	515	1202	33	64
Q9D9V3	Ethylmalonyl-CoA decarboxylase	4.3640	2.1420	5.5560	2.6710	2.6260	0.0364	0.1050	0.0002	0.0071	0.0012	322	273	13	50
Q62264	Thyroid hormone-inducible hepatic protein	4.8280	1.8240	4.6420	4.0150	2.7160	0.0140	0.1773	0.0007	0.0001	0.0004	150	339	4	27
Q9CZW4	Long-chain-fatty-acid--CoA ligase 3	5.5340	3.1750	4.2170	3.0870	3.5440	0.0216	0.0625	0.0186	0.0210	0.0071	720	11	2	6
P30115	Glutathione S-transferase A3	4.3710	1.5760	3.6090	2.5700	1.9050	0.0302	0.3667	0.0072	0.0098	0.0168	221	494	9	66
Q8BMA6	Signal recognition particle subunit SRP68	6.6970	2.4720	5.1770	5.0800	3.8200	0.0079	0.1839	0.0043	0.0024	0.0028	625	78	6	14
Q91WU0	Carboxylesterase 1F	4.1300	1.5210	3.9940	3.1410	2.2630	0.0295	0.1801	0.0016	0.0023	0.0016	561	485	21	55
Q9CQE1	Protein NipSnap homolog 3B	6.2150	2.4780	4.6480	2.7480	2.3820	0.0089	0.1063	0.0051	0.0120	0.0200	247	141	6	22
Q8VEK3	Heterogeneous nuclear ribonucleoprotein U	3.3090	1.9760	2.5600	2.1710	1.6010	0.0848	0.1113	0.0334	0.0222	0.0279	800	565	23	36
Q8CAY6	Acetyl-CoA acetyltransferase, cytosolic	3.3110	1.6730	3.1930	2.2290	1.8640	0.1158	0.2800	0.0153	0.0193	0.0179	397	518	13	53
Q3UPL0	Protein transport protein Sec31A	3.3070	1.9540	2.5960	2.0580	1.6860	0.0837	0.1231	0.0463	0.0415	0.0276	1230	594	35	39
P35505	Fumarylacetoacetase	3.0240	1.7190	2.9250	2.0960	1.5630	0.1548	0.2217	0.0248	0.0378	0.0478	419	572	14	51
P35385	Heat shock protein beta-7	4.9200	2.3800	2.5470	2.1900	2.1820	0.0164	0.0392	0.0554	0.0292	0.0032	169	538	9	76
Q62009	Periostin	5.6720	3.3440	2.2260	2.7320	2.0690	0.0061	0.0064	0.1038	0.0064	0.0037	838	1131	37	64
P08226	Apolipoprotein E	4.0670	3.2780	2.2580	2.0970	1.6100	0.0316	0.0037	0.0716	0.0322	0.0267	311	975	20	64

Q3UZ39	Leucine-rich repeat flightless-interacting protein 1	5.4330	4.4310	2.6730	3.1470	2.7820	0.0127	0.0032	0.1152	0.0111	0.0115	729	10	5	9
P31996	Macrosialin	5.1130	8.7360	2.2350	3.0030	4.4020	0.0206	0.0000	0.2532	0.0175	0.0005	326	48	3	14
Q9Z2X1	Heterogeneous nuclear ribonucleoprotein F	4.5390	2.8440	2.6530	2.5790	1.8710	0.0353	0.0222	0.0612	0.0104	0.0223	415	398	9	40
Q8BXA1	Golgi integral membrane protein 4	5.7380	4.1820	3.0360	4.4300	3.0910	0.0174	0.0149	0.1060	0.0042	0.0106	655	103	2	4
Q6WVG3	BTB/POZ domain-containing protein KCTD12	4.3690	2.5780	2.6160	2.1940	1.6900	0.0333	0.0405	0.0698	0.0299	0.0460	327	350	11	41
Q9CQE5	Regulator of G-protein signaling 10	6.5900	4.3170	2.9630	4.3960	2.9830	0.0087	0.0122	0.1043	0.0037	0.0151	181	60	3	25
Q9WUM4	Coronin-1C	4.2700	2.4100	2.3920	2.5710	2.0150	0.0345	0.0410	0.0821	0.0111	0.0092	474	342	15	35
Q9WUU7	Cathepsin Z	4.6680	3.1050	2.4410	2.8220	1.8530	0.0187	0.0070	0.0604	0.0034	0.0090	306	450	9	47
P97797	Tyrosine-protein phosphatase non-receptor type substrate 1	4.0870	2.4270	2.3690	2.8970	2.5150	0.0480	0.0380	0.1126	0.0030	0.0014	513	33	5	10
Q569Z6	Thyroid hormone receptor-associated protein 3	3.9170	3.5110	2.9340	3.7180	2.5960	0.0756	0.0183	0.0636	0.0016	0.0114	951	71	9	11
Q5F2E7	Nuclear fragile X mental retardation-interacting protein 2	4.3440	3.4610	3.0840	8.6870	4.8100	0.0706	0.0404	0.0946	0.0000	0.0008	692	41	2	3
Q9Z0J0	NPC intracellular cholesterol transporter 2	3.5950	2.6740	2.5450	1.9940	1.6450	0.0794	0.0275	0.0678	0.0476	0.0486	149	205	8	54
Q99PL5	Ribosome-binding protein 1	3.8300	1.8520	2.3040	2.2900	1.6190	0.0412	0.0722	0.0596	0.0155	0.0257	1605	1686	48	34
Q62313	Trans-Golgi network integral membrane protein 1	4.8740	2.7100	3.5080	3.4800	2.5620	0.0399	0.1384	0.0556	0.0186	0.0359	353	27	2	6
Q9EQU5	Protein SET	4.1810	2.1660	2.6030	2.1940	1.7600	0.0407	0.0701	0.0512	0.0205	0.0323	289	396	10	43
Q64449	C-type mannose receptor 2	4.0930	1.7660	2.2430	2.7990	1.8040	0.0361	0.1238	0.0686	0.0050	0.0115	1479	463	23	21
Q921F2	TAR DNA-binding protein 43	2.9350	1.9670	2.1090	2.0660	1.5880	0.1538	0.1265	0.1346	0.0265	0.0302	414	615	14	50
Q91ZX7	Prolow-density lipoprotein receptor-related protein 1	3.1390	1.8520	2.2900	2.0640	1.6300	0.0917	0.0970	0.0616	0.0408	0.0245	4545	1237	76	20
Q9CYZ2	Tumor protein D54	2.7480	1.7950	2.0890	2.2150	1.6080	0.2282	0.1854	0.1526	0.0295	0.0493	220	429	12	51
Q9D0J8	Parathymosin	3.1890	1.7590	2.2100	2.6650	1.6040	0.1171	0.1973	0.1072	0.0066	0.0271	101	161	5	24
Q05793	Basement membrane-specific heparan sulfate proteoglycan core protein	3.6470	1.9830	2.3690	2.0230	1.7470	0.0506	0.0504	0.0514	0.0310	0.0147	3707	3595	94	38
Q64314	Hematopoietic progenitor cell antigen CD34	3.1510	1.9620	2.1350	2.4540	1.7380	0.1615	0.1651	0.2057	0.0133	0.0401	382	226	7	17
Q9QYC0	Alpha-adducin	3.1440	1.8490	2.7240	2.1830	1.9420	0.1783	0.2388	0.0797	0.0257	0.0289	735	217	13	27
Q8BJ71	Nuclear pore complex protein Nup93	3.1760	1.6770	2.3720	2.7160	1.9720	0.1695	0.3851	0.1385	0.0072	0.0328	819	22	5	7
B2RXS4	Plexin-B2	3.5670	2.9190	3.1670	2.7640	2.3290	0.1057	0.0628	0.0594	0.0309	0.0414	1842	19	6	5
Q99ME6	Protein ATP1B4	4.0600	2.4050	2.0130	2.4800	2.0630	0.0618	0.1131	0.3320	0.0471	0.0477	356	78	4	13
Q8C650	Septin-10	2.9970	1.8530	2.5190	3.5900	2.7540	0.2497	0.4392	0.1743	0.0153	0.0168	452	60	2	6
Q9R059	Four and a half LIM domains protein 3	2.4750	2.2760	2.6460	2.5830	2.2330	0.3527	0.0718	0.0654	0.0088	0.0043	289	305	12	46

Q61093	Cytochrome b-245 heavy chain	3.0440	2.3970	2.1150	3.4290	2.2550	0.1977	0.1149	0.2802	0.0031	0.0389	570	45	14	44
Q8CI95	Oxysterol-binding protein-related protein 11	3.1970	2.6030	3.3630	3.0550	2.5270	0.1884	0.1524	0.0670	0.0403	0.0336	751	24	5	10
Q9CQW9	Interferon-induced transmembrane protein 3	4.6770	3.1600	3.7060	2.1520	2.7540	0.0368	0.0321	0.0137	0.0521	0.0034	137	105	2	20
A6H5X4	PHD finger protein 11	9.9150	31.4940	6.7800	2.2150	4.5560	0.0015	0.0000	0.0016	0.0992	0.0039	337	13	2	12
P08207	Protein S100-A10	4.0830	2.6840	3.2880	1.6200	1.6450	0.0565	0.0257	0.0144	0.1542	0.0437	97	250	5	71
Q9Z1F9	SUMO-activating enzyme subunit 2	3.2850	2.5360	3.0530	1.7910	1.5570	0.0872	0.0312	0.0160	0.0832	0.0337	638	162	12	29
Q8R0X7	Sphingosine-1-phosphate lyase 1	5.2310	2.7180	3.3450	1.8940	2.2120	0.0246	0.0662	0.0392	0.1625	0.0442	568	41	3	6
Q8K4G1	Latent-transforming growth factor beta-binding protein 4	4.7020	2.7570	3.4900	2.4220	2.0470	0.0397	0.0752	0.0306	0.0613	0.0491	1666	73	7	5
Q9CZN7	Serine hydroxymethyltransferase, mitochondrial	4.3190	2.7530	4.0570	2.2280	3.1480	0.0535	0.0777	0.0101	0.0882	0.0029	504	70	7	23
P97927	Laminin subunit alpha-4	2.9810	1.7430	2.5050	1.8770	1.6270	0.1109	0.0975	0.0378	0.0620	0.0248	1816	631	32	23
Q8JZU2	Tricarboxylate transport protein, mitochondrial	3.1830	1.6970	3.7540	1.8430	2.1270	0.1293	0.2365	0.0050	0.0688	0.0029	311	345	15	55
Q9D2R0	Acetoacetyl-CoA synthetase	2.6730	1.5480	3.1950	1.5530	1.8270	0.2863	0.4415	0.0172	0.1838	0.0263	672	247	20	43
Q62093	Serine/arginine-rich splicing factor 2	3.6470	2.1010	3.9810	1.6250	2.1920	0.0829	0.1776	0.0061	0.2077	0.0144	221	152	6	22
P54310	Hormone-sensitive lipase	3.6790	1.9030	4.4220	1.9270	2.1910	0.0685	0.1208	0.0007	0.0583	0.0022	759	695	30	63
P33267	Cytochrome P450 2F2	3.5440	1.6910	3.0300	1.8990	1.8080	0.0827	0.3047	0.0320	0.0695	0.0270	491	396	22	53
O70503	Very-long-chain 3-oxoacyl-CoA reductase	4.4180	2.6740	3.6480	2.3020	2.4790	0.0527	0.0700	0.0205	0.0619	0.0167	312	95	7	29
Q6P069	Sorcin	3.0580	2.2000	2.6780	1.9400	1.6930	0.1487	0.0646	0.0438	0.0562	0.0304	198	379	10	46
Q99JR1	Sideroflexin-1	3.3510	2.3980	3.5590	1.5020	2.2220	0.1291	0.1748	0.0234	0.3726	0.0413	322	73	7	41
Q5SWU9	Acetyl-CoA carboxylase 1	3.1480	1.8020	3.6800	1.6270	1.8460	0.0907	0.0829	0.0030	0.0898	0.0096	2345	2783	101	66
Q5EBG6	Heat shock protein beta-6	3.0260	1.7610	2.7880	1.8640	1.9020	0.1135	0.1922	0.0201	0.0623	0.0075	162	597	8	71
Q9ET01	Glycogen phosphorylase, liver form	2.7640	1.7940	2.8110	1.6310	1.7050	0.1446	0.0848	0.0191	0.0889	0.0177	850	3782	32	61
P51881	ADP/ATP translocase 2	3.1280	1.7040	2.7210	1.6350	1.5840	0.0929	0.1087	0.0233	0.0878	0.0300	298	4970	13	74
Q8BTM8	Filamin-A	3.6810	2.0590	2.1870	1.7110	1.8430	0.0487	0.0410	0.0778	0.0715	0.0097	2647	3788	119	65
P16675	Lysosomal protective protein	4.0230	2.7710	2.4200	1.8210	1.7930	0.0530	0.0175	0.0816	0.0864	0.0269	474	503	10	25
Q9Z1X4	Interleukin enhancer-binding factor 3	2.8990	2.5590	2.5540	1.9110	1.7130	0.1729	0.0249	0.0561	0.0718	0.0241	898	291	18	28
P42227	Signal transducer and activator of transcription 3	3.7130	3.0590	2.6100	1.6670	2.4570	0.0944	0.0463	0.1274	0.2682	0.0247	770	112	8	19
O89103	Complement component C1q receptor	3.4140	2.5370	2.5850	1.9000	1.9200	0.1187	0.0415	0.0820	0.0724	0.0251	644	264	9	19
Q6Q899	Probable ATP-dependent RNA helicase DDX58	4.9540	2.4250	3.2810	2.5210	2.4940	0.0252	0.1873	0.0634	0.0604	0.0279	926	114	10	13
Q6NVF9	Cleavage and polyadenylation specificity factor subunit 6	4.2440	2.6570	2.8000	2.3060	2.2270	0.0457	0.1071	0.0956	0.0706	0.0420	551	124	5	14

P01900	H-2 class I histocompatibility antigen, D-D alpha chain	3.9820	1.5950	1.8570	1.6680	1.7640	0.0474	0.2974	0.2289	0.1099	0.0132	365	401	12	55
P01902	H-2 class I histocompatibility antigen, K-D alpha chain	3.5290	2.3320	2.6810	1.7310	1.7390	0.0891	0.0583	0.0563	0.1114	0.0328	368	345	16	48
P16546	Spectrin alpha chain, non-erythrocytic 1	2.9350	1.7310	2.0200	1.7780	1.5340	0.1173	0.1008	0.1144	0.0596	0.0373	2472	4234	141	64
P61750	ADP-ribosylation factor 4	4.0550	1.5260	2.4650	1.8320	1.8220	0.0590	0.5009	0.0991	0.0812	0.0428	180	498	5	83
Q9JIF0	Protein arginine N-methyltransferase 1	2.6650	1.8970	2.1530	1.7370	1.6700	0.2642	0.1511	0.1425	0.0843	0.0361	371	500	14	41
Q640N1	Adipocyte enhancer-binding protein 1	4.6470	7.5350	3.3940	2.6010	1.8610	0.0410	0.0000	0.0432	0.0393	0.1033	1128	63	8	9
Q80VQ0	Aldehyde dehydrogenase family 3 member B1	4.7930	3.1980	4.1180	2.4150	1.5960	0.0248	0.0086	0.0044	0.0147	0.0941	468	104	4	35
P31324	cAMP-dependent protein kinase type II-beta regulatory subunit	2.8910	2.2810	2.9920	2.2380	1.5830	0.2540	0.1578	0.0466	0.0443	0.1451	416	314	7	26
Q922Q8	Leucine-rich repeat-containing protein 59	4.7910	3.5320	2.4700	2.5910	1.8810	0.0218	0.0144	0.1334	0.0099	0.0723	307	183	9	32
P52840	Sulfotransferase 1A1	5.7020	2.9880	2.6830	2.4580	1.5780	0.0069	0.0142	0.0603	0.0139	0.0742	291	8	2	12
Q91XV3	Brain acid soluble protein 1	5.4940	3.0730	2.6680	2.9400	1.6980	0.0094	0.0235	0.0805	0.0055	0.0666	226	230	6	39
P21995	Embigin	4.0600	4.5000	2.7740	3.3410	1.9560	0.0703	0.0088	0.1151	0.0102	0.1073	330	17	3	9
Q8BPU7	Engulfment and cell motility protein 1	3.4140	6.8820	2.6920	3.7130	2.2130	0.1476	0.0007	0.1354	0.0070	0.0762	727	5	2	3
Q8BHC4	Dephospho-CoA kinase domain-containing protein	2.3530	3.7440	2.3660	3.1870	1.7330	0.4223	0.0253	0.2174	0.0235	0.1671	231	12	3	25
Q62318	Transcription intermediary factor 1-beta	3.6160	2.9730	2.0570	2.7510	1.7870	0.1142	0.0440	0.2911	0.0243	0.1047	834	86	7	16
Q9JJU8	SH3 domain-binding glutamic acid-rich-like protein	3.6170	2.5390	1.9060	2.2920	1.6280	0.0984	0.0427	0.2800	0.0242	0.0641	114	150	7	79
P70429	Ena/VASP-like protein	6.3110	3.0020	2.7360	3.7520	2.2070	0.0128	0.0806	0.1425	0.0102	0.0713	414	49	6	16
Q9D8S3	ADP-ribosylation factor GTPase-activating protein 3	3.5660	2.4720	2.5020	2.9850	2.1240	0.1469	0.1803	0.2039	0.0304	0.0751	523	17	2	7
Q8R464	Cell adhesion molecule 4	3.3650	1.8710	2.2840	2.7110	1.5280	0.1693	0.3770	0.2422	0.0347	0.2547	388	7	2	6
P70313	Nitric oxide synthase, endothelial	3.1630	1.8920	2.7180	2.6620	1.6590	0.1628	0.1828	0.0593	0.0077	0.0653	1202	9	2	1
Q9DBE0	Cysteine sulfinic acid decarboxylase	3.1430	1.6590	2.6370	2.7100	1.6990	0.1781	0.3808	0.0749	0.0061	0.0507	493	289	15	48
P33434	72 kDa type IV collagenase	4.2310	2.8120	2.7050	2.8600	1.7430	0.0587	0.1156	0.1271	0.0266	0.1483	662	52	7	15
O08738	Caspase-6	3.5880	2.5690	2.5230	2.9380	2.2260	0.1315	0.1558	0.1996	0.0453	0.0696	276	27	5	15
P02798	Metallothionein-2	3.5110	2.2510	2.7430	3.0780	2.2320	0.1431	0.2517	0.1218	0.0187	0.0502	61	56	2	33
Q9QZD8	Mitochondrial dicarboxylate carrier	3.0540	2.6070	2.0560	2.1730	1.5510	0.2112	0.0673	0.2137	0.0450	0.1055	287	162	9	47
Q6ZPZ3	Zinc finger CCCH domain-containing protein 4	2.5470	2.6940	2.0370	2.5470	1.5360	0.3510	0.1258	0.3383	0.0461	0.2845	1304	21	2	3
Q9Z1Q5	Chloride intracellular channel protein 1	3.5140	2.1080	2.3480	2.0140	1.5760	0.0803	0.0843	0.0872	0.0495	0.0504	241	446	16	80
Q61545	RNA-binding protein EWS	3.3360	2.3720	2.7400	2.2060	1.7120	0.1396	0.0739	0.0701	0.0296	0.0822	655	143	4	8

O88792	Junctional adhesion molecule A	3.3950	3.0790	2.7610	2.8650	2.2140	0.1666	0.0744	0.1382	0.0461	0.0666	300	30	2	3
Q61581	Insulin-like growth factor-binding protein 7	5.6820	4.6970	5.2100	2.4320	1.9820	0.0177	0.0079	0.0045	0.0887	0.1038	281	46	3	19
P29416	Beta-hexosaminidase subunit alpha	4.5380	2.8760	5.5290	2.0490	1.8030	0.0376	0.0799	0.0020	0.1254	0.1505	528	113	10	27
Q9WV80	Sorting nexin-1	3.1590	2.6360	3.2700	1.6090	1.9350	0.1944	0.0812	0.0296	0.2691	0.0610	522	99	7	16
Q91VS7	Microsomal glutathione S-transferase 1	3.2690	1.9180	2.9860	1.5420	1.6120	0.1302	0.1669	0.0351	0.1827	0.0620	155	141	8	66
Q8R5A0	N-lysine methyltransferase SMYD2	1.9770	1.8550	3.2750	2.0870	1.5800	0.6699	0.3141	0.0353	0.1010	0.2009	433	77	6	14
Q9CXR1	Dehydrogenase/reductase SDR family member 7	3.1340	2.2180	3.8460	1.6630	1.5590	0.1744	0.1739	0.0106	0.2283	0.1272	338	119	10	39
Q9R0Q9	Mannose-P-dolichol utilization defect 1 protein	3.6780	2.2210	3.3580	1.9320	1.6750	0.0943	0.2551	0.0449	0.1753	0.1839	247	38	3	14
Q9DBL7	Bifunctional coenzyme A synthase	2.6670	2.0550	2.9820	2.0310	1.6940	0.3071	0.1893	0.0477	0.0715	0.1119	563	48	6	19
D3Z6Q9	Bridging integrator 2	11.1610	3.9410	2.6530		1.7540	0.0007	0.0213	0.1572		0.2210	489	155	2	7
P01921	H-2 class II histocompatibility antigen, A-D beta chain	4.9990	3.6960	2.2300	2.3410	1.8470	0.0237	0.0125	0.2439	0.0599	0.0864	265	154	9	41
Q60766	Immunity-related GTPase family M protein 1	4.5440	3.6320	3.0690	1.8890	1.6910	0.0415	0.0162	0.0507	0.1234	0.1311	409	69	10	25
Q9CXY6	Interleukin enhancer-binding factor 2	3.8510	2.9920	2.5280	1.8650	1.6710	0.0770	0.0303	0.1071	0.0944	0.1075	390	146	10	33
P61290	Proteasome activator complex subunit 3	4.1650	2.9170	2.3160	2.2070	1.7890	0.0504	0.0499	0.2274	0.0864	0.1329	254	75	5	23
P01915	H-2 class II histocompatibility antigen, E-D beta chain	2.4110	4.6250	2.5900	1.6640	1.5420	0.4430	0.0092	0.1566	0.3156	0.2488	264	19	6	27
P47964	60S ribosomal protein L36	3.3860	3.9610	2.4300	1.8450	1.6720	0.1308	0.0109	0.1698	0.2207	0.1725	105	24	3	21
P50543	Protein S100-A11	3.7720	1.7650	2.9460	2.0700	1.4710	0.0619	0.1924	0.0216	0.0367	0.0486	98	417	6	72
Q8BG05	Heterogeneous nuclear ribonucleoprotein A3	3.3450	2.2220	1.9960	1.8820	1.4940	0.0718	0.0264	0.1209	0.0450	0.0445	379	1463	19	48
Q9WUM3	Coronin-1B	3.5150	1.9110	2.0420	2.0870	1.4940	0.0589	0.0976	0.1086	0.0414	0.0444	484	388	15	41
Q9WVA4	Transgelin-2	3.4010	1.8540	2.1010	2.1100	1.4780	0.0673	0.0718	0.0948	0.0247	0.0477	199	1225	17	81
P32020	Non-specific lipid-transfer protein	2.5820	1.7060	2.2910	1.8650	1.4720	0.1812	0.1639	0.0615	0.0550	0.0490	547	748	13	19
Q9DCD0	6-phosphogluconate dehydrogenase, decarboxylating	3.4870	2.1430	2.7600	2.0490	1.4360	0.0609	0.0326	0.0213	0.0289	0.0572	483	1826	29	73
Q9WTP6	Adenylate kinase 2, mitochondrial	2.7030	1.6760	2.5960	1.8670	1.4050	0.1559	0.1668	0.0308	0.0465	0.0657	239	1432	19	69
Q9CPW4	Actin-related protein 2/3 complex subunit 5	6.2280	3.9310	2.9010	2.1520	1.2560	0.0049	0.0019	0.0527	0.0431	0.3906	151	153	7	60
P15379	CD44 antigen	5.5090	2.7170	2.8950	3.2910	1.0890	0.0168	0.0722	0.0765	0.0082	0.6294	778	77	4	5
Q6GU68	Immunoglobulin superfamily containing leucine-rich repeat protein	4.6870			2.9440	1.0760	0.0351			0.0345	0.6884	428	55	6	23
P30681	High mobility group protein B2	3.2710	2.4540	2.2600	2.1600	1.4350	0.1508	0.0895	0.1828	0.0329	0.2015	210	82	5	17
P28063	Proteasome subunit beta type-8	3.3640	1.6920	2.6000	2.2570	1.4060	0.1278	0.3077	0.0727	0.0238	0.1319	276	197	11	46

P61979	Heterogeneous nuclear ribonucleoprotein K	2.9250	1.9680	2.1380	1.8450	1.4510	0.1187	0.0526	0.0872	0.0497	0.0537	463	1705	24	57
Q6Y7W8	GRB10-interacting GYF protein 2	3.8870	1.7460	2.6870	3.1150	1.4780	0.1021	0.3834	0.1119	0.0159	0.2385	1291	30	2	2
P11688	Integrin alpha-5	2.6070	2.2380	1.6630	2.5330	1.4980	0.3627	0.2139	0.6113	0.0464	0.2736	1053	70	4	4
Q9WV91	Prostaglandin F2 receptor negative regulator	2.4290	1.9820	1.6550	2.2940	1.1580	0.3764	0.1394	0.5021	0.0195	0.3385	879	131	11	18
P26645	Myristoylated alanine-rich C-kinase substrate	3.4080	1.7250	2.0350	2.0270	1.4260	0.0667	0.1536	0.1105	0.0380	0.0598	309	719	12	64
P70245	3-beta-hydroxysteroid-Delta(8),Delta(7)-isomerase	2.0310	2.5540	2.1500	4.8860	1.4650	0.6251	0.1616	0.3237	0.0008	0.3160	230	17	3	25
P09055	Integrin beta-1	2.5640	2.0860	1.8990	1.9040	1.4380	0.2511	0.0788	0.2156	0.0494	0.0567	798	897	26	39
E9Q7G0	Nuclear mitotic apparatus protein 1	2.2710	1.6420	1.6870	2.0750	1.4980	0.4095	0.3348	0.4080	0.0425	0.0802	2094	315	30	20
Q9EST5	Acidic leucine-rich nuclear phosphoprotein 32 family member B	3.9010	2.9750	3.1140	1.9210	1.4250	0.0596	0.0171	0.0211	0.0591	0.1372	272	229	5	33
O54931	A-kinase anchor protein 2	3.9970	1.8440	4.4890	2.1480	1.2380	0.0840	0.3477	0.0060	0.0819	0.4814	893	44	5	9
Q8R422	CD109 antigen	3.1940	1.7760	7.0800	1.9660	1.1890	0.1881	0.4803	0.0003	0.1508	0.5682	1442	15	4	4
Q9D0B5	Thiosulfate sulfurtransferase/rhodanese-like domain-containing protein 3	5.5420	3.7630	3.4940		0.6120	0.0255	0.0248	0.0534		0.4582	157	31	3	25
P18242	Cathepsin D	4.0480	2.4120	2.1640	1.8270	1.2540	0.0323	0.0159	0.0821	0.0522	0.1261	410	1082	12	42
P62962	Profilin-1	3.0630	1.9900	1.9910	1.7650	1.4010	0.1004	0.0495	0.1223	0.0617	0.0667	140	1512	11	77
P62889	60S ribosomal protein L30	2.6790	5.1140	2.2940	1.9630	1.1580	0.2977	0.0017	0.1911	0.1179	0.4210	115	110	6	54
P18760	Cofilin-1	3.5570	2.4370	2.3510	1.6350	1.4190	0.0562	0.0166	0.0536	0.1287	0.0617	166	1516	12	70
Q60972	Histone-binding protein RBBP4	3.1610	2.3540	1.9590	1.6120	1.2530	0.1312	0.0485	0.2549	0.1369	0.2328	425	440	9	67
P27773	Protein disulfide-isomerase A3	2.9480	2.0750	1.8690	1.7660	1.2020	0.1154	0.0392	0.1618	0.0615	0.1576	505	2006	33	71
Q61543	Golgi apparatus protein 1	2.8000	2.1440	1.7730	1.5300	1.1210	0.1384	0.0325	0.2015	0.1174	0.2223	1175	533	22	19
P09541	Myosin light chain 4	6.7090	2.8270	3.0820	2.1110	1.2720	0.0059	0.0618	0.0597	0.0971	0.4310	193	232	5	47
Q9D8Y0	EF-hand domain-containing protein D2	4.5340	2.1280	2.2430	1.8080	1.4320	0.0380	0.1716	0.2141	0.1279	0.2562	240	167	9	32
P63276	40S ribosomal protein S17	4.7140	2.6100	3.3610	1.4700	2.1780	0.0239	0.0301	0.0127	0.2457	0.0066	135	251	6	56
P62702	40S ribosomal protein S4, X isoform	2.8010	2.2970	3.0200	0.8270	1.5090	0.1381	0.0472	0.0121	0.9265	0.0417	263	575	23	68
P62245	40S ribosomal protein S15a	3.1460	2.5980	3.2890	0.9590	1.6520	0.1509	0.0362	0.0123	0.8913	0.0467	130	261	9	69
P14131	40S ribosomal protein S16	3.6060	3.9040	3.3870	1.0490	1.8130	0.0934	0.0018	0.0132	0.7065	0.0220	146	266	6	40
Q9D1R9	60S ribosomal protein L34	3.8770	1.8400	3.2710	0.8930	2.1410	0.0846	0.3160	0.0376	0.8845	0.0357	117	200	6	38
P62900	60S ribosomal protein L31	3.0890	1.5640	3.0150	0.8020	1.6970	0.1622	0.3937	0.0328	0.7289	0.0467	125	94	3	20
P62270	40S ribosomal protein S18	3.3050	1.9980	3.1420	1.0770	1.6910	0.0826	0.0808	0.0093	0.5197	0.0187	152	385	10	45

P01791	Ig heavy chain V region HPCM6	5.7710	10.0630	2.1530	1.1660	2.6920	0.0181	0.0000	0.2877	0.7234	0.0149	123	36	3	49
Q91VC3	Eukaryotic initiation factor 4A-III	3.7150	1.8850	2.1730	1.3920	2.0740	0.1055	0.2992	0.2307	0.4432	0.0494	411	246	7	26
P62908	40S ribosomal protein S3	2.8900	1.8890	2.3810	0.9130	1.5330	0.1239	0.0653	0.0501	0.6358	0.0375	243	873	21	80
Q99N92	39S ribosomal protein L27, mitochondrial	2.1030	1.7060	2.4350	0.7720	2.7400	0.5914	0.4407	0.1720	0.6249	0.0133	148	21	4	38
Q5FWK3	Rho GTPase-activating protein 1	3.9220	2.1770	2.5430	1.3480	1.8010	0.0588	0.1468	0.1025	0.3690	0.0470	439	229	12	42
Q6ZWV3	60S ribosomal protein L10	2.9060	1.5680	2.6240	0.8320	1.6290	0.1967	0.3930	0.0689	0.8203	0.0468	214	254	9	46
P70261	Paladin	2.6600	2.0540	2.9770	1.2300	3.0450	0.3052	0.3246	0.0922	0.5968	0.0176	859	6	2	4
Q9CPR4	60S ribosomal protein L17	3.4170	1.7740	2.4700	0.9200	1.7190	0.0987	0.2544	0.0929	0.9671	0.0359	184	159	7	42
P31725	Protein S100-A9	16.8290	8.3610	4.9260	1.2210	1.9980	0.0000	0.0000	0.0028	0.6515	0.0544	113	212	9	74
Q9JIK9	28S ribosomal protein S34, mitochondrial	2.9510	2.4650	3.1960	1.4900	1.6650	0.2241	0.1071	0.0420	0.3734	0.1465	218	10	2	10
P12970	60S ribosomal protein L7a	3.3780	2.1190	3.4240	0.6410	1.5440	0.1189	0.0843	0.0112	0.4030	0.0719	266	251	13	42
Q64471	Glutathione S-transferase theta-1	3.4850	2.2150	4.0890	1.3580	1.9820	0.1229	0.2217	0.0142	0.5320	0.0788	240	118	2	19
P61255	60S ribosomal protein L26	3.7880	3.0190	2.5800	0.7230	1.5700	0.0852	0.0315	0.0962	0.4664	0.1050	145	138	7	43
Q9EPB4	Apoptosis-associated speck-like protein containing a CARD	3.4860	4.7940	2.7150	0.8100	1.6720	0.1187	0.0016	0.0771	0.6025	0.1277	193	121	10	66
Q9D0T1	NHP2-like protein 1	4.2150	4.7400	2.9270	1.4290	1.5510	0.0656	0.0021	0.0689	0.4433	0.2185	128	107	5	62
Q91WV0	Protein Dr1	3.0680	5.6590	2.3610	1.3630	1.5550	0.2068	0.0027	0.2250	0.4759	0.2788	176	5	2	18
Q61599	Rho GDP-dissociation inhibitor 2	4.5140	3.1140	2.7340	1.3850	1.3870	0.0283	0.0067	0.0448	0.2937	0.1424	200	311	12	66
P0DOV1	Interferon-activable protein 205-B	7.5130	5.6720	4.6810	0.9380	1.1380	0.0062	0.0029	0.0132	0.9774	0.6322	425	32	2	18
P35979	60S ribosomal protein L12	4.1020	2.7270	2.8560	0.7290	1.4900	0.0425	0.0249	0.0438	0.5214	0.0999	165	192	7	59
Q9CZX8	40S ribosomal protein S19	3.2980	2.3490	2.6970	1.2110	1.4440	0.0807	0.0417	0.0246	0.3551	0.0552	145	521	9	54
P47911	60S ribosomal protein L6	3.0120	2.4280	2.6720	0.7370	1.3670	0.1461	0.0343	0.0382	0.6593	0.0787	296	440	19	47
P63325	40S ribosomal protein S10	2.9070	2.8430	3.0100	0.8840	1.3980	0.2187	0.0222	0.0324	0.9447	0.1395	165	222	7	43
P97351	40S ribosomal protein S3a	2.7140	1.8890	2.5010	0.8990	1.3600	0.1692	0.1456	0.0381	0.8383	0.0797	264	668	18	59
P25444	40S ribosomal protein S2	2.8780	1.5110	2.6290	0.8690	1.1040	0.1629	0.3375	0.0327	0.9917	0.2389	293	535	16	53
P33622	Apolipoprotein C-III	2.1670	1.9240	3.1840	1.3080	1.2990	0.5507	0.2482	0.0417	0.4672	0.2795	99	139	2	27
P68254	14-3-3 protein theta	2.8750	1.7510	2.5320	1.3720	1.3670	0.1694	0.2035	0.0497	0.2694	0.0773	245	1548	11	70
Q8K2H2	Deubiquitinase OTUD6B	2.0680	1.6420	4.2000	1.3460	1.2610	0.6244	0.4677	0.0120	0.4813	0.4652	294	24	3	17
O55142	60S ribosomal protein L35a	1.9080	1.7830	2.7460	1.1710	1.4180	0.6063	0.1993	0.0357	0.4719	0.0621	110	189	6	45

P62242	40S ribosomal protein S8	2.9660	1.9590	2.8000	0.8480	1.4710	0.1531	0.1158	0.0342	0.8811	0.0768	208	381	13	59
Q3V1T4	Prolyl 3-hydroxylase 1	7.0240	4.1190	2.3240	0.7530	1.2040	0.0075	0.0153	0.2539	0.6706	0.5327	739	60	5	9
P61514	60S ribosomal protein L37a	5.8430	3.1870	2.5870	0.6880	1.4660	0.0136	0.0277	0.1199	0.5496	0.2711	92	66	6	47
P97742	Carnitine O-palmitoyltransferase 1, liver isoform	2.0260	3.5410	1.9150	0.7240	1.0670	0.6545	0.0369	0.4466	0.6316	0.7163	773	82	5	13
Q9CR57	60S ribosomal protein L14	3.3510	2.8750	2.4910	0.6670	1.4490	0.1208	0.0206	0.0877	0.3661	0.1183	217	170	7	32
Q8CCF0	U4/U6 small nuclear ribonucleoprotein Prp31	2.3230	4.9180	2.2850	0.8310	0.8500	0.4699	0.0063	0.2783	0.8311	0.9186	499	11	3	11
P21958	Antigen peptide transporter 1	1.9800	4.3040	1.6330	1.0460	1.3400	0.6761	0.0054	0.6510	0.8348	0.4218	724	17	5	10
Q99MD9	Nuclear autoantigenic sperm protein	3.1210	3.9110	2.6080	1.4970	1.2170	0.2050	0.0257	0.1755	0.4024	0.5261	773	10	2	5
P53026	60S ribosomal protein L10a	4.1180	2.0190	2.0760	0.8150	1.2810	0.0445	0.1048	0.1511	0.8886	0.1303	217	210	8	38
P56528	ADP-ribosyl cyclase/cyclic ADP-ribose hydrolase 1	13.1920	6.0100	2.2890	0.6410	0.5740	0.0000	0.0005	0.2605	0.4593	0.4002	304	11	2	8
P27005	Protein S100-A8	3.4140	40.1770	12.3490	0.4680	4.4190	0.1549	0.0000	0.0000	0.2096	0.0010	89	201	7	82
Q6ZWV7	60S ribosomal protein L35	3.4880	1.7520	3.1450	0.5350	1.6700	0.1126	0.2674	0.0259	0.1606	0.0454	123	120	4	28
Q9CZM2	60S ribosomal protein L15	3.0940	3.0650	2.7540	0.5690	1.8120	0.1655	0.0192	0.0542	0.1796	0.0323	204	244	11	43
P47963	60S ribosomal protein L13	3.3650	2.5160	3.6440	0.5640	1.5500	0.1152	0.0393	0.0105	0.1844	0.0786	211	146	7	35
Q8R3F5	Malonyl-CoA-acyl carrier protein transacylase, mitochondrial	3.3540	2.0140	2.7330	0.0910	0.1730	0.1562	0.3670	0.1472	0.0000	0.0009	381	63	2	6
Q9R0P6	Signal peptidase complex catalytic subunit SEC11A	1.5990	2.4370		0.4060	0.2570	0.9680	0.1553		0.0940	0.0153	179	10	3	26
Q6WKZ8	E3 ubiquitin-protein ligase UBR2	4.9260	2.8870		0.4980	0.3430	0.0330	0.0951		0.2050	0.0685	1755	9	2	1
P24668	Cation-dependent mannose-6-phosphate receptor	2.5930	2.2830	0.8060	3.6290	2.2810	0.3077	0.0826	0.3234	0.0006	0.0053	278	53	3	11
P01831	Thy-1 membrane glycoprotein	3.7860	1.8490	1.3280	2.1000	1.6070	0.0769	0.1956	0.8144	0.0409	0.0627	162	148	4	31
Q9DBR3	Armadillo repeat-containing protein 8		3.6250	0.9560		1.8940		0.0332	0.5877		0.1889	673	3	2	4
Q99LX8	Dolichyl-diphosphooligosaccharide--protein glycosyltransferase subunit 4	2.7810	3.4380	0.9400	1.6380	2.1300	0.2982	0.0447	0.5710	0.2869	0.0830	37	28	2	100
P21614	Vitamin D-binding protein	2.8290	2.5220	1.4630	1.7870	1.2520	0.1335	0.0119	0.4055	0.0654	0.1276	476	896	24	61
P47856	Glutamine--fructose-6-phosphate aminotransferase [isomerizing] 1	2.4500	7.4320	1.3310	1.8370	0.6190	0.3759	0.0004	0.9703	0.2026	0.4209	697	30	10	20
Q91VH2	Sorting nexin-9	1.9630	3.0200	1.3480	1.8030	0.0100	0.6726	0.0764	0.8631	0.2534	0.0000	595	3	3	7
P97369	Neutrophil cytosol factor 4		3.0000	0.8370		0.5320		0.0324	0.4461		0.2966	339	5	3	14
Q9JI91	Alpha-actinin-2	2.5150	1.6130	1.3320	1.2090	1.4840	0.1969	0.1398	0.5386	0.2853	0.0464	894	4531	47	79
Q8VDW0	ATP-dependent RNA helicase DDX39A	1.5840	3.6790	0.6890	1.2740	0.8050	0.9691	0.0118	0.2578	0.5586	0.8323	427	592	3	37

O70439	Syntaxin-7	2.0130	2.7760	1.3820	1.2200	0.9540	0.4086	0.0211	0.7063	0.6096	0.8572	261	74	7	30
P06800	Receptor-type tyrosine-protein phosphatase C	1.8230	6.3650	1.3160	0.8910	0.6940	0.8142	0.0006	0.9468	0.7835	0.5777	1293	125	24	28
Q3UND0	Src kinase-associated phosphoprotein 2	1.8190	3.3000	1.1430	1.0580	0.5970	0.7714	0.0494	0.8720	0.8329	0.4199	358	20	6	19
Q571E4	N-acetylgalactosamine-6-sulfatase	4.0590	3.4730	1.2570	0.4940		0.0937	0.0408	0.9781	0.2353		520	25	4	10
E9QAT4	Protein transport protein Sec16A	4.0220	2.0510	1.3610	0.0100	1.2410	0.0833	0.3292	0.8813	0.0000	0.5223	2357	23	2	1
Q6NV83	U2 snRNP-associated SURP motif-containing protein	1.7460	4.3350	1.1940	0.5550	0.7570	0.7840	0.0127	0.9100	0.2832	0.7272	1029	6	2	2
O35892	Nuclear autoantigen Sp-100	1.8600	2.6500	1.2140	0.3940	0.0100	0.7852	0.0702	0.9217	0.0953	0.0000	482	4	2	3
Q62293	T-cell-specific guanine nucleotide triphosphate-binding protein 1	13.5040	3.0690	0.8400	0.5950	0.4700	0.0001	0.0724	0.3961	0.4030	0.2043	415	29	9	36
P56542	Deoxyribonuclease-2-alpha	1.9500	4.9450	0.4600	0.7760	1.0510	0.6983	0.0017	0.0726	0.7197	0.7535	353	8	2	4
Q3URD3	Sarcolemmal membrane-associated protein	4.2720	1.4620	3.7190	2.7330	2.1770	0.0441	0.5129	0.0085	0.0062	0.0075	845	32	6	10
Q8CGN5	Perilipin-1	3.4190	1.3800	4.2250	2.7650	2.2740	0.0659	0.4487	0.0010	0.0029	0.0016	517	1444	30	71
Q8R0Y6	Cytosolic 10-formyltetrahydrofolate dehydrogenase	3.4020	1.4700	3.4590	2.4820	1.9210	0.0672	0.3511	0.0047	0.0135	0.0069	902	1412	44	68
P04117	Fatty acid-binding protein, adipocyte	2.9490	1.4530	2.5980	2.2080	1.7600	0.1153	0.2171	0.0306	0.0192	0.0139	132	7545	8	65
Q6NZB0	DnaJ homolog subfamily C member 8	2.8700	1.3930	2.3180	1.8980	1.6060	0.1811	0.4731	0.0817	0.0442	0.0271	253	119	7	23
Q9WVL0	Maleylacetoacetate isomerase	2.8520	1.2520	2.9320	1.8980	1.6200	0.1298	0.5902	0.0146	0.0646	0.0255	216	956	14	82
Q05421	Cytochrome P450 2E1	2.8290	1.3930	3.1390	1.6290	1.9530	0.2892	0.7046	0.0297	0.2395	0.0335	493	100	15	44
P52196	Thiosulfate sulfurtransferase	2.3490	1.2480	2.2100	1.9310	1.5390	0.2681	0.5942	0.0739	0.0573	0.0365	297	640	11	44
P50462	Cysteine and glycine-rich protein 3	2.3260	0.7900	1.7060	1.6690	1.7870	0.3362	0.4424	0.3397	0.1176	0.0117	194	395	9	62
O55028	[3-methyl-2-oxobutanoate dehydrogenase [lipoamide]] kinase, mitochondrial		1.0170	1.5520	2.5300	2.9570		0.6311	0.7338	0.0689	0.0184	412	28	4	17
Q8R059	UDP-glucose 4-epimerase	2.3810	1.4200	2.1020	2.7020	1.5970	0.4181	0.6803	0.2700	0.0196	0.1622	347	79	6	27
P50285	Dimethylaniline monooxygenase [N-oxide-forming] 1	2.5650	1.3810	2.7640	1.6560	1.5800	0.2800	0.5990	0.0399	0.1193	0.0620	532	413	18	41
Q9Z1T2	Thrombospondin-4	2.8100	1.4090	2.1240	1.7390	1.4900	0.1366	0.2530	0.0900	0.0662	0.0452	963	1230	23	37
Q66K08	Cartilage intermediate layer protein 1	4.0240	1.2820	1.7160	2.4270	1.1610	0.0505	0.6144	0.3816	0.0147	0.2678	1184	300	15	15
Q91V76	Ester hydrolase C11orf54 homolog	2.8340	1.3810	2.1110	1.9760	1.3890	0.1327	0.3535	0.0928	0.0295	0.0703	315	417	14	70
P99024	Tubulin beta-5 chain	3.2920	1.3660	2.5300	1.2990	1.6660	0.0765	0.2753	0.0357	0.2224	0.0209	444	1511	4	73
O54724	Caveolae-associated protein 1	2.7890	1.4400	2.6710	1.3420	1.5030	0.1403	0.2335	0.0260	0.2414	0.0427	392	1107	15	42
Q05920	Pyruvate carboxylase, mitochondrial	2.3190	1.2110	2.5440	1.1560	1.5280	0.2652	0.5676	0.0346	0.4319	0.0384	1178	1055	46	54
Q63918	Caveolae-associated protein 2	2.2180	1.2230	2.4650	1.4240	1.5610	0.3088	0.6302	0.0413	0.2020	0.0332	418	542	11	33

P17225	Polypyrimidine tract-binding protein 1	3.6340	1.3990	2.2000	1.4740	1.5640	0.0685	0.4585	0.1135	0.2046	0.0324	527	365	9	39
O35678	Monoglyceride lipase	2.2240	1.1680	2.5380	1.1650	1.3540	0.3698	0.6976	0.0351	0.4116	0.0819	303	708	15	67
Q60714	Long-chain fatty acid transport protein 1	1.8580	1.3290	2.6000	1.1530	1.3390	0.6776	0.5605	0.0496	0.5108	0.1159	646	319	21	45
Q9CQE7	Endoplasmic reticulum-Golgi intermediate compartment protein 3	2.0250	1.4740	5.7200	1.1230	1.0320	0.6565	0.6217	0.0007	0.7639	0.7188	383	5	2	4
P68369	Tubulin alpha-1A chain	2.8130	1.4250	2.3830	1.1020	1.4160	0.1361	0.3208	0.0497	0.4829	0.0625	451	1279	6	69
P62862	40S ribosomal protein S30	2.3680	0.8050	1.1540	2.7210	2.1390	0.4495	0.3360	0.8424	0.0428	0.0643	59	9	2	19
Q9D0L8	mRNA cap guanine-N7 methyltransferase	1.9150	1.1300	0.6580	3.0560	0.7910	0.7099	0.9577	0.1423	0.0143	0.7715	465	19	3	10
P02535	Keratin, type I cytoskeletal 10	5.1140	1.1510	0.7790	1.6760	0.7450	0.0248	0.9550	0.2955	0.1970	0.6752	570	29	3	8
P70444	BH3-interacting domain death agonist	2.2440	0.8960	1.0420	1.1450	0.3230	0.5052	0.5779	0.6645	0.7702	0.0198	195	5	3	19
P41241	Tyrosine-protein kinase CSK	1.8350	1.3350	1.2980	0.4040	0.2580	0.7920	0.8276	0.9962	0.1359	0.0108	450	12	7	24
O54782	Epididymis-specific alpha-mannosidase	1.7580	0.7790	1.3960	0.3980	0.0100	0.8596	0.3578	0.8646	0.1063	0.0000	1018	4	2	3
Q9Z2V4	Phosphoenolpyruvate carboxykinase, cytosolic [GTP]		1.4930	0.8880	0.5200	0.3410		0.7093	0.4566	0.2092	0.0411	622	12	3	8
Q8BQ30	Phostensin	2.8750	1.1950	0.3570	0.5090	0.6240	0.2535	0.9310	0.0167	0.2543	0.4968	594	9	2	4
P16460	Argininosuccinate synthase		0.0670	0.0130	0.0100	0.0110		0.0000	0.0000	0.0000	0.0000	412	18	7	18
Q61686	Chromobox protein homolog 5	0.8540		1.5650	3.0640	1.1860	0.2399		0.6737	0.0182	0.5632	191	19	3	27
P97494	Glutamate--cysteine ligase catalytic subunit	1.3300	3.5140	2.5390	2.0120	1.4690	0.7222	0.0146	0.1453	0.1199	0.2715	637	24	4	8
O88879	Apoptotic protease-activating factor 1	1.4650	11.7060	1.6810	0.0100	0.9670	0.9317	0.0000	0.6059	0.0000	0.9211	1249	4	2	2
Q01320	DNA topoisomerase 2-alpha	1.2710	3.6580	1.7620	0.0100	0.0100	0.7716	0.0324	0.5075	0.0000	0.0000	1528	4	2	1
O35640	Annexin A8	0.6480			0.5490	0.2370	0.1042			0.2939	0.0072	327	3	3	10
O70145	Neutrophil cytosol factor 2	1.1690	10.9640	0.8260	1.2510		0.6567	0.0000	0.4139	0.5724		525	21	5	13
P35175	Stefin-1	1.1770	27.6570	1.2850	1.4970	1.3640	0.6330	0.0000	0.9904	0.3964	0.3771	97	31	3	40
Q8K426	Resistin-like gamma	1.2150	13.8600	1.1910	0.8100	1.3570	0.7100	0.0000	0.9590	0.7987	0.4031	117	10	2	23
Q99KV1	DnaJ homolog subfamily B member 11	1.2490	2.6600	1.0810	0.6910	0.0100	0.7495	0.1145	0.7720	0.5408	0.0000	358	3	2	11
Q3THE2	Myosin regulatory light chain 12B	1.1630	4.7560	1.1140	0.6820	0.5280	0.6136	0.0025	0.7549	0.5619	0.2935	172	7	2	12
P48025	Tyrosine-protein kinase SYK	0.7680	2.3050	0.6630	0.4350	0.2710	0.2193	0.1873	0.2006	0.1506	0.0103	629	13	5	12
O09043	Napsin-A	0.6410	5.7550	1.1410	0.4890	0.4530	0.1299	0.0006	0.8927	0.2099	0.1852	419	18	5	28
Q61549	Adhesion G protein-coupled receptor E1	1.2150	12.2490	0.0100	1.7540	0.0100	0.6633	0.0000	0.0000	0.2588	0.0000	931	4	2	4
P10810	Monocyte differentiation antigen CD14	1.3400	8.5740	0.5320	0.7420	0.7560	0.7940	0.0002	0.1056	0.6514	0.7260	366	37	9	38

Q8BTV2	Cleavage and polyadenylation specificity factor subunit 7	0.9220	1.6470	0.0100	0.5190	0.7260	0.3589	0.6197	0.0000	0.2806	0.6931	471	22	2	7
P28293	Cathepsin G	0.6430	2.3410	0.3480	0.3210	0.3150	0.1265	0.1243	0.0132	0.0544	0.0368	261	22	7	30
O70456	14-3-3 protein sigma	0.8920	1.0840	1.8180	0.1890	0.0670	0.2676	0.9553	0.3322	0.0001	0.0000	248	524	3	32
Q6P9Q6	FK506-binding protein 15	0.7070	0.8560	1.5710	0.3050	0.5360	0.1049	0.4883	0.7065	0.0076	0.2540	1216	16	3	3
Q9CQN6	Transmembrane protein 14C	0.8260	0.8610	0.9810	0.3580	0.8510	0.1744	0.5147	0.5585	0.0434	0.9127	114	95	5	75
Q9CQG1	Putative glutathione-specific gamma-glutamylcyclotransferase 2	1.2910	0.7360	0.8790	0.5840	0.3180	0.7241	0.2833	0.4697	0.3677	0.0260	178	5	2	11
Q9WVG6	Histone-arginine methyltransferase CARM1	0.7310	0.8460	0.6600	0.4500	0.2160	0.1431	0.4927	0.1549	0.1199	0.0019	608	11	2	4
P01820	Ig heavy chain V region PJ14	0.7160	0.7440	0.9040	0.3660	0.5480	0.1438	0.3370	0.4384	0.0482	0.2733	115	26	2	28
Q8JZS9	39S ribosomal protein L48, mitochondrial	0.9060	0.7010	0.8350	0.3270	0.3890	0.2701	0.2720	0.3533	0.0113	0.0535	211	17	3	23
P97825	Jupiter microtubule associated homolog 1	1.3740	0.9690	0.9010	0.2830	0.4480	0.7719	0.6839	0.4500	0.0042	0.1159	154	42	3	38
Q8BFQ4	WD repeat-containing protein 82	0.7890	0.6050	0.9970	0.3920	0.4210	0.1658	0.1619	0.6079	0.0319	0.0840	313	26	3	9
Q3UIU2	NADH dehydrogenase [ubiquinone] 1 beta subcomplex subunit 6	1.0440	0.6450	0.9150	0.4190	0.5970	0.4032	0.2148	0.4873	0.0454	0.3807	128	136	3	25
Q921L3	Calcium load-activated calcium channel	0.7070	0.7480	0.6940	0.2610	0.3750	0.1560	0.3320	0.2009	0.0165	0.0646	188	8	2	15
Q6P9J9	Anoctamin-6	1.2620	1.0020	0.0100	2.0800	0.7200	0.7171	0.6355	0.0000	0.1546	0.6586	911	3	2	6
Q9JK38	Glucosamine 6-phosphate N-acetyltransferase	0.8390	0.7220	0.3150	0.8690	0.8040	0.2789	0.2899	0.0072	0.8849	0.8275	184	20	3	41
P50428	Arylsulfatase A	0.9670	0.6430	0.4650	0.7310	0.6670	0.3209	0.2013	0.0280	0.5160	0.5032	506	18	4	14
Q9QXK3	Coatomer subunit gamma-2	0.6930	0.7110	0.0100	0.7050	0.0100	0.3107	0.6702	0.0000	0.9421	0.0000	871	46	2	6
Q3TTY5	Keratin, type II cytoskeletal 2 epidermal	0.9860	0.6170	0.3330	1.2160	0.2300	0.4104	0.1608	0.0106	0.6534	0.0050	707	17	4	6
P00687	Alpha-amylase 1	1.2870	1.1870	0.4210	0.6570	0.3920	0.7422	0.9680	0.0365	0.5031	0.0968	511	3	2	5
Q8K1X4	Nck-associated protein 1-like	1.0600	1.3220	0.0110	0.0100	0.7170	0.4503	0.8291	0.0000	0.0000	0.6079	1134	13	3	5
P48999	Arachidonate 5-lipoxygenase	0.6470	1.0420	0.4000	0.3370	0.8460	0.1271	0.7207	0.0308	0.0739	0.9073	674	7	4	6
P18528	Ig heavy chain V region 6.96	0.6660	0.7400	0.3380	0.5070	0.8190	0.0693	0.3138	0.0047	0.1300	0.8527	98	38	2	42
Q91Z83	Myosin-7	0.8030	0.6320	0.4780	0.1880	0.2770	0.1816	0.1941	0.0366	0.0002	0.0065	1935	516	3	15
Q60648	Ganglioside GM2 activator	0.7300	0.7890	0.5510	0.3760	0.2670	0.1156	0.4114	0.0658	0.0304	0.0073	193	11	2	16
Q9JHK5	Pleckstrin	1.0140	1.1970	0.2930	0.4270	0.3310	0.3743	0.9828	0.0014	0.0544	0.0217	350	29	5	26
O08692	Neutrophilic granule protein	0.8530	1.3910	0.5920	0.4100	0.3150	0.2797	0.4756	0.1225	0.0786	0.0164	167	96	11	60
Q9CY45	EEF1A lysine methyltransferase 1	0.8490	0.6550	0.5520	0.4950	0.2550	0.2711	0.2244	0.0909	0.2309	0.0068	214	6	2	15
Q9WV60	Glycogen synthase kinase-3 beta	0.7990	0.6070	0.5770	0.4440	0.3610	0.1762	0.1771	0.0852	0.0525	0.0367	420	135	4	24

Q9D7I5	Phospholysine phosphohistidine inorganic pyrophosphate phosphatase	0.6100	0.6130	0.5920	0.4030	0.3630	0.0879	0.1808	0.1199	0.0789	0.0430	270	369	10	62
Q60716	Prolyl 4-hydroxylase subunit alpha-2	0.8490	0.8900	0.5710	0.5300	0.3500	0.1847	0.5309	0.0975	0.2063	0.0386	537	9	5	12
Q69Z23	Dynein heavy chain 17, axonemal	1.0340	0.6740	0.5940	0.4050	0.4030	0.4029	0.2584	0.1063	0.0304	0.0624	4481	107	2	0
P63300	Selenoprotein W	0.7140	0.7780	0.5080	0.4730	0.4060	0.1135	0.3658	0.0488	0.0843	0.0702	88	115	3	50
P13542	Myosin-8	0.7040	0.6400	0.4710	0.4590	0.3870	0.1301	0.2061	0.0319	0.1040	0.0548	1937	1448	3	31
Q08093	Calponin-2	0.9170	0.9400	0.4880	0.4920	0.4460	0.2607	0.7017	0.0353	0.1371	0.1094	305	32	4	22
P70158	Acid sphingomyelinase-like phosphodiesterase 3a	0.9040	0.8130	0.4620	0.5390	0.4980	0.3012	0.4925	0.0292	0.2168	0.2225	445	56	5	22
Q91ZR1	Ras-related protein Rab-4B	1.2980	0.7300	0.3510	0.5680	0.5740	0.6961	0.3347	0.0084	0.2781	0.3194	213	3	2	11
P01723	Ig lambda-1 chain V region	0.7400	0.8380	0.4880	0.5810	0.5720	0.1423	0.5314	0.0399	0.2983	0.4073	117	26	3	42
P48772	Cytochrome c oxidase subunit 8B, mitochondrial	0.7530	0.2670	0.7180	0.2990	0.4800	0.2249	0.0006	0.3246	0.0110	0.2479	70	366	3	56
O08600	Endonuclease G, mitochondrial	0.7820	0.5500	0.8040	0.3600	0.5270	0.1920	0.1074	0.3645	0.0278	0.2949	294	261	10	36
Q8C0L9	Glycerophosphocholine phosphodiesterase GPCPD1	0.6480	0.4640	1.0670	0.5030	0.4210	0.0772	0.0409	0.7157	0.1048	0.0947	675	12	4	8
Q9R0G6	Cartilage oligomeric matrix protein	0.6990	0.4280	0.6330	0.4250	0.4520	0.1695	0.0492	0.2000	0.1143	0.2024	755	449	17	35
P51912	Neutral amino acid transporter B(0)	0.8620	0.4430	1.0650	0.3550	0.3880	0.2620	0.0363	0.7032	0.0683	0.0841	553	16	4	16
Q8BLY2	Threonine--tRNA ligase 2, cytoplasmic	0.9100	0.4130	0.9760	0.5160	0.5800	0.2650	0.0248	0.5736	0.1183	0.3347	790	101	6	11
Q9EPU0	Regulator of nonsense transcripts 1	1.0040	0.4350	0.4750	0.7950	0.6600	0.3747	0.0352	0.0341	0.6600	0.5338	1124	67	8	10
P0C0A3	Charged multivesicular body protein 6	0.7890	0.5220	0.4050	0.7590	0.6200	0.1588	0.0685	0.0134	0.5321	0.4112	200	7	3	18
Q69ZS7	HBS1-like protein	0.8260	0.5010	0.0360	0.7380	0.5980	0.2047	0.0732	0.0000	0.5284	0.3825	682	11	2	4
P01654	Ig kappa chain V-III region PC 2880/PC 1229	0.7100	0.5140	0.3940	0.2260	0.3050	0.0986	0.0529	0.0075	0.0006	0.0071	111	87	4	63
Q3V0K9	Plastin-1	0.7660	0.4170	0.5260	0.4410	0.2510	0.1544	0.0240	0.0564	0.0497	0.0033	630	159	2	16
Q8R0W0	Epiplakin	0.6930	0.4310	0.4210	0.4810	0.3870	0.0988	0.0183	0.0138	0.1189	0.0441	6548	215	2	4
Q8BMJ3	Eukaryotic translation initiation factor 1A, X-chromosomal	0.7560	0.3560	0.5130	0.5900	0.3240	0.1380	0.0058	0.0442	0.2960	0.0131	144	76	5	36
E9Q555	E3 ubiquitin-protein ligase RNF213	0.7730	0.5880	0.4190	0.4690	0.1750	0.1824	0.1377	0.0151	0.1288	0.0003	5152	29	3	1
Q9D0M5	Dynein light chain 2, cytoplasmic	0.7110	0.5550	0.4920	0.4340	0.3250	0.1055	0.1035	0.0371	0.0704	0.0151	89	605	2	79
Q9QXZ0	Microtubule-actin cross-linking factor 1	0.6130	0.5040	0.4760	0.3680	0.2250	0.0644	0.0907	0.0358	0.0612	0.0025	7354	37	8	2
Q91VW3	SH3 domain-binding glutamic acid-rich-like protein 3	0.6430	0.5010	0.2500	0.4940	0.3340	0.0756	0.0516	0.0002	0.1469	0.0197	93	81	4	68
P52760	2-iminobutanoate/2-iminopropanoate deaminase	0.6020	0.5450	0.5660	0.4230	0.3830	0.0434	0.0873	0.0836	0.0615	0.0451	135	67	6	54
Q80VJ3	2'-deoxynucleoside 5'-phosphate N-hydrolase 1	0.6160	0.5450	0.5250	0.3910	0.3620	0.0568	0.1100	0.0558	0.0593	0.0421	173	47	3	25

Q5SX39	Myosin-4	0.6340	0.5960	0.5500	0.3610	0.3370	0.2404	0.4036	0.1518	0.1359	0.0375	1939	2260	24	54
Q9JM99	Proteoglycan 4	0.6340	0.3880	0.5990	0.2440	0.5120	0.0784	0.0141	0.0995	0.0005	0.2008	1054	70	7	22
Q925I1	ATPase family AAA domain-containing protein 3	0.6070	0.5510	0.5200	0.2880	0.4300	0.1082	0.0932	0.1098	0.0207	0.1578	591	10	2	4
P21956	Lactadherin	1.0260	0.5330	0.5360	0.3300	0.4840	0.3857	0.1129	0.0761	0.0497	0.1709	463	5	3	6
Q8R550	SH3 domain-containing kinase-binding protein 1	0.7450	0.5990	0.4600	0.4550	0.3990	0.1217	0.1390	0.0199	0.0930	0.0514	709	81	6	11
Q91WG0	Acylcarnitine hydrolase	0.6420	0.5060	0.3490	0.4490	0.4410	0.2496	0.2245	0.0130	0.3043	0.1805	561	36	9	26
Q9CQ86	Migration and invasion enhancer 1	0.8690	0.5090	0.4710	0.5060	0.4530	0.2268	0.0675	0.0326	0.1037	0.1351	115	21	4	38
Q9D2M8	Ubiquitin-conjugating enzyme E2 variant 2	0.7820	0.5860	0.5150	0.4820	0.5510	0.1544	0.1252	0.0418	0.1207	0.3206	145	424	3	61
Q91W59	RNA-binding motif, single-stranded-interacting protein 1	0.7020	0.4590	0.5730	0.4300	0.4890	0.0883	0.0472	0.0820	0.0816	0.1796	403	5	3	8
P30355	Arachidonate 5-lipoxygenase-activating protein	0.4890	2.8320	1.9670	2.2310	1.0610	0.0285	0.0980	0.4113	0.1141	0.7395	161	26	5	58
P83870	PHD finger-like domain-containing protein 5A	0.4890	1.7630	1.7130	1.0220	0.5980	0.0305	0.4767	0.5836	0.8784	0.4113	110	11	3	23
P51437	Cathelicidin antimicrobial peptide	0.5410	16.3250	1.8370	0.5350	0.5910	0.0464	0.0000	0.4617	0.2465	0.3513	172	14	3	25
Q8VCI0	Phospholipase B-like 1	0.5830	2.1910	0.5930	0.5810	0.3870	0.0347	0.1794	0.0884	0.2589	0.0615	550	79	18	43
Q921E2	Ras-related protein Rab-31	0.5620	0.6370		0.2810		0.0635	0.2029		0.0231		194	36	2	24
Q3UQS2	Leucine-rich single-pass membrane protein 1	0.1670	0.8990	0.6300	0.6810	0.4790	0.0000	0.5794	0.1378	0.3763	0.1555	128	13	2	17
Q9Z1T1	AP-3 complex subunit beta-1	0.4330	0.9490	0.6740	0.5960	0.6420	0.0076	0.7660	0.1873	0.3097	0.5594	1105	40	6	8
Q99L20	Glutathione S-transferase theta-3	0.5090	0.7510	0.7080	0.2440	0.2480	0.0216	0.3516	0.1993	0.0094	0.0052	241	18	2	24
Q7TSC1	Protein PRRC2A	0.4390	0.6270	0.6530	0.1790	0.2330	0.0307	0.1503	0.2037	0.0012	0.0111	2158	9	2	2
Q7TNV0	Protein DEK	0.5000	0.7150	0.6890	0.3850	0.3600	0.0306	0.3362	0.2351	0.0565	0.0420	380	39	2	7
Q5I2A0	Serine protease inhibitor A3G	0.5910	1.4310	0.6360	0.3140	0.4330	0.0867	0.7766	0.1816	0.0418	0.1410	440	363	2	12
Q8R4E4	Myozenin-3	0.5630	0.7130	0.6260	0.4380	0.4600	0.0497	0.3060	0.1369	0.0758	0.1609	245	163	8	40
Q80W21	Glutathione S-transferase Mu 7	0.4860	0.6000	0.7720	0.4610	0.4880	0.0371	0.1771	0.3080	0.1512	0.1766	218	1194	2	51
Q9QZQ8	Core histone macro-H2A.1	0.3390	0.6430	0.9540	0.4120	0.4640	0.0036	0.1785	0.5194	0.0708	0.1488	372	10	2	5
Q9R099	Transducin beta-like protein 2	0.3450	1.0940	0.1930			0.0094	0.7653	0.0004			442	4	2	4
Q8R5F7	Interferon-induced helicase C domain-containing protein 1	0.4760	0.6890	0.2000	0.1400	0.2320	0.0263	0.2820	0.0001	0.0002	0.0033	1025	12	2	2
Q8BFY6	Peflin	0.5300	0.7370	0.4920	0.3660	0.2120	0.0308	0.3312	0.0389	0.0190	0.0009	275	25	3	12
P18181	CD48 antigen	0.4490	1.1380	0.4110	0.2130	0.2400	0.0210	0.8821	0.0316	0.0056	0.0068	240	5	2	7
O70200	Allograft inflammatory factor 1	0.5040	0.6460	0.5300	0.2960	0.2010	0.0243	0.2159	0.0619	0.0070	0.0005	147	8	2	14
P00375	Dihydrofolate reductase	0.5770	0.9380	0.4940	0.4680	0.3620	0.0406	0.6481	0.0393	0.0841	0.0400	187	7	2	14

Q8BHJ5	F-box-like/WD repeat-containing protein TBL1XR1	0.5940	0.8030	0.3370	0.3600	0.3290	0.0571	0.4221	0.0042	0.0555	0.0270	514	9	3	5
A2VDH3	Leucine-rich repeat-containing protein 38	0.0100	0.9380	0.5940	0.5290	0.0100	0.0000	0.5368	0.1620	0.3104	0.0000	298	4	2	9
Q64282	Interferon-induced protein with tetratricopeptide repeats 1	0.5740	0.9930	0.5240	0.3230	0.5300	0.0797	0.7116	0.0769	0.0453	0.2469	463	7	5	12
A2A863	Integrin beta-4	0.0650	0.0920		0.0240		0.0000	0.0000		0.0000		1818	6	3	2
Q80XN0	D-beta-hydroxybutyrate dehydrogenase, mitochondrial	0.4940	0.4490	0.6260	0.4090	0.2930	0.0240	0.0378	0.1377	0.0328	0.0074	343	22	5	15
P01636	Ig kappa chain V-V region MOPC 149	0.2830	0.5320	0.7240	0.2490	0.2920	0.0020	0.1093	0.2191	0.0140	0.0143	108	10	2	26
Q8CHS7	Dehydrogenase/reductase SDR family member 7C	0.4880	0.5230	0.6940	0.3680	0.3450	0.0408	0.1365	0.2918	0.0513	0.0435	311	361	16	61
P10922	Histone H1.0	0.4170	0.2900	0.6430	0.2640	0.4410	0.0071	0.0009	0.1557	0.0029	0.1186	194	181	5	28
Q8R1Q3	Angiopoietin-related protein 7	0.5980	0.4510	0.7930	0.0100	0.5720	0.1054	0.0376	0.3581	0.0000	0.3660	337	3	2	5
Q9R020	Zinc finger Ran-binding domain-containing protein 2	0.4320	0.3660	0.6860	0.5730	0.4580	0.0298	0.0107	0.2389	0.3412	0.2052	330	2	2	6
Q09014	Neutrophil cytosol factor 1	0.4080	0.5000	0.6110	0.4100	0.5380	0.0058	0.0662	0.1230	0.0558	0.2998	390	26	9	19
Q8VEA4	Mitochondrial intermembrane space import and assembly protein 40	0.5410	0.5180	0.4580	0.6780	0.5150	0.0408	0.0619	0.0289	0.4725	0.2086	139	70	4	42
P68134	Actin, alpha skeletal muscle	0.3640	0.3390	0.2450	0.2190	0.2660	0.0304	0.0318	0.0010	0.0107	0.0063	377	12040	8	89
Q5ND34	WD repeat-containing protein 81	0.4340	0.4690	0.3680	0.3780	0.2100	0.0124	0.0410	0.0060	0.0393	0.0004	1934	19	2	1
P09542	Myosin light chain 3	0.1810	0.1710	0.1730	0.1530	0.3260	0.0006	0.0002	0.0000	0.0009	0.0296	204	1136	10	70
P61458	Pterin-4-alpha-carbinolamine dehydratase	0.0100	0.0100	0.0100	0.0100	0.0100	0.0000	0.0000	0.0000	0.0000	0.0000	104	32	3	32
Q99KW9	T-cell immunomodulatory protein	0.2730	0.2370	0.2120	0.1830	0.1430	0.0004	0.0007	0.0001	0.0004	0.0000	610	3	2	8
P01639	Ig kappa chain V-V region MOPC 41	0.4240	0.4210	0.2790	0.1670	0.1330	0.0091	0.0306	0.0013	0.0001	0.0000	130	5	2	28
P58771	Tropomyosin alpha-1 chain	0.3060	0.2530	0.1840	0.2130	0.2560	0.0132	0.0048	0.0001	0.0091	0.0045	284	4093	13	68
Q64112	Interferon-induced protein with tetratricopeptide repeats 2	0.1770	0.4330	0.1080	0.0590	0.1250	0.0000	0.0349	0.0000	0.0000	0.0000	472	13	6	17
P21107	Tropomyosin alpha-3 chain	0.3900	0.3120	0.2980	0.2810	0.3140	0.0412	0.0191	0.0043	0.0436	0.0230	285	1688	15	69
Q05186	Reticulocalbin-1	0.2280	0.2780	0.2150	0.2250	0.1900	0.0002	0.0023	0.0001	0.0003	0.0001	325	13	3	10
P19123	Troponin C, slow skeletal and cardiac muscles	0.3030	0.2500	0.1320	0.1820	0.3010	0.0056	0.0012	0.0000	0.0009	0.0169	161	220	8	55
Q6W8Q3	Purkinje cell protein 4-like protein 1	0.3280	0.3230	0.2380	0.2640	0.2880	0.0016	0.0054	0.0003	0.0033	0.0092	68	24	2	32
Q9WUZ5	Troponin I, slow skeletal muscle	0.1220	0.0880	0.0880	0.0660	0.2120	0.0000	0.0000	0.0000	0.0000	0.0008	187	147	7	41
Q99J47	Dehydrogenase/reductase SDR family member 7B	0.2180	0.1380	0.2100	0.1270	0.0790	0.0001	0.0000	0.0001	0.0000	0.0000	323	8	2	7
Q8R2E9	ERO1-like protein beta	0.4060	0.1220	0.0920	0.3910	0.0800	0.0043	0.0000	0.0000	0.0364	0.0000	467	4	2	7
Q9JLH8	Tropomodulin-4	0.4690	0.3890	0.3580	0.3230	0.2950	0.0350	0.0331	0.0113	0.0368	0.0146	345	511	8	44

Q8K2Q5	Coiled-coil-helix-coiled-coil-helix domain-containing protein 7	0.4820	0.3830	0.4240	0.2640	0.2890	0.0287	0.0276	0.0209	0.0112	0.0130	85	3	2	19
Q9JL62	Glycolipid transfer protein	0.2340	0.2110	0.1370	0.1610	0.2100	0.0031	0.0011	0.0000	0.0014	0.0007	209	35	2	19
O88346	Troponin T, slow skeletal muscle	0.4340	0.3770	0.4060	0.2320	0.2030	0.0643	0.0382	0.0327	0.0060	0.0005	262	453	10	44
Q9QZ47	Troponin T, fast skeletal muscle	0.4740	0.3480	0.2560	0.2650	0.2900	0.0907	0.0371	0.0014	0.0321	0.0125	272	4577	13	36
P58774	Tropomyosin beta chain	0.4160	0.2960	0.2350	0.2460	0.2940	0.0540	0.0138	0.0007	0.0214	0.0141	284	3802	18	66
Q8BZA9	Fructose-2,6-bisphosphatase TIGAR	0.3800	0.4950	0.4790	0.3120	0.3320	0.0092	0.0755	0.0495	0.0188	0.0280	269	322	10	59
P05977	Myosin light chain 1/3, skeletal muscle isoform	0.3960	0.3750	0.2510	0.2580	0.2940	0.0438	0.0559	0.0012	0.0277	0.0142	188	4378	15	74
P70695	Fructose-1,6-bisphosphatase isozyme 2	0.3070	0.4310	0.3410	0.2380	0.2820	0.0135	0.1116	0.0112	0.0176	0.0100	339	974	14	63
P35487	Pyruvate dehydrogenase E1 component subunit alpha, testis-specific form, mitochondrial	0.5290	0.5470	0.3900	0.3990	0.3510	0.0217	0.0884	0.0092	0.0490	0.0343	391	99	2	12
Q8CC35	Synaptopodin	0.5360	0.5170	0.4150	0.3490	0.3560	0.0242	0.0692	0.0134	0.0192	0.0300	929	151	13	16
Q9CU62	Structural maintenance of chromosomes protein 1A	0.4710	0.5420	0.4080	0.2990	0.2300	0.0374	0.1208	0.0296	0.0457	0.0054	1233	4	3	4
Q9JIK5	Nucleolar RNA helicase 2	0.4890	0.4950	0.3930	0.3180	0.2360	0.0302	0.0785	0.0149	0.0246	0.0033	851	4	2	5
Q8C494	Proline-rich protein 33	0.4260	0.4690	0.4070	0.2970	0.3370	0.0250	0.0961	0.0229	0.0158	0.0374	260	432	16	63
Q8VCR8	Myosin light chain kinase 2, skeletal/cardiac muscle	0.3040	0.4160	0.4660	0.2670	0.2980	0.0128	0.0948	0.0696	0.0331	0.0155	613	967	22	53
Q99J08	SEC14-like protein 2	0.5430	0.5550	0.5900	0.3160	0.3160	0.0293	0.1141	0.0980	0.0064	0.0175	403	21	2	4
O08911	Mitogen-activated protein kinase 12	0.5300	0.5140	0.5920	0.3530	0.3490	0.0628	0.1185	0.1542	0.0400	0.0475	367	441	13	53
Q8CD91	SPARC-related modular calcium-binding protein 2	0.5730	0.5430	0.5680	0.3320	0.3150	0.0807	0.0783	0.1389	0.0420	0.0405	447	7	2	4
Q8C5H8	NAD kinase 2, mitochondrial	0.4060	0.2930	0.2460	0.3820	0.3040	0.0146	0.0063	0.0009	0.0703	0.0171	452	4	2	6
O55239	Nicotinamide N-methyltransferase	0.4820	0.3450	0.4480	0.5110	0.3690	0.0187	0.0100	0.0256	0.1370	0.0358	264	20	2	8
Q9WUR9	Adenylate kinase 4, mitochondrial	0.3000	0.3310	0.5160	0.4210	0.3020	0.0018	0.0104	0.0463	0.0836	0.0156	223	22	3	13
Q8R1G2	Carboxymethylenebutenolidase homolog	0.3040	0.3980	0.3910	0.3320	0.3420	0.0129	0.0760	0.0263	0.0954	0.0413	245	787	13	66
P13412	Troponin I, fast skeletal muscle	0.4090	0.3770	0.2650	0.2970	0.3110	0.0503	0.0572	0.0018	0.0568	0.0213	182	4779	12	49
Q8CI70	Leucine-rich repeat-containing protein 20	0.4740	0.5290	0.4220	0.3230	0.3380	0.0904	0.2665	0.0409	0.0840	0.0380	184	443	10	73
P97457	Myosin regulatory light chain 2, skeletal muscle isoform	0.4160	0.4480	0.2950	0.3170	0.3180	0.0539	0.1342	0.0041	0.0778	0.0248	169	3239	20	91
Q9WUZ7	SH3 domain-binding glutamic acid-rich protein	0.4750	0.4100	0.3620	0.3360	0.3380	0.0911	0.0769	0.0163	0.0655	0.0384	214	511	9	64
Q9D7X8	Gamma-glutamylcyclotransferase	0.5620	0.5050	0.5430	0.5140	0.3930	0.0402	0.0401	0.0583	0.1659	0.0466	188	78	5	34
Q08091	Calponin-1	0.5320	0.5250	0.5970	0.4320	0.3900	0.0318	0.0524	0.0933	0.0665	0.0462	297	26	4	24
Q9ERT9	Protein phosphatase 1 regulatory subunit 1A	0.4760	0.5340	0.5380	0.4210	0.3220	0.0190	0.0759	0.0638	0.0768	0.0191	171	79	4	27

P50431	Serine hydroxymethyltransferase, cytosolic	0.5560	0.5230	0.5920	0.4170	0.3470	0.0769	0.1339	0.1534	0.1056	0.0455	478	136	11	35
Q3TMH2	Secernin-3	0.4970	0.5210	0.4810	0.3670	0.3250	0.1074	0.2514	0.0810	0.1448	0.0289	418	993	20	53
Q64669	NAD(P)H dehydrogenase [quinone] 1	0.3930	0.4130	0.4190	0.3320	0.3880	0.0047	0.0126	0.0168	0.0157	0.0541	274	139	10	42
P51667	Myosin regulatory light chain 2, ventricular/cardiac muscle isoform	0.3030	0.2880	0.2390	0.2330	0.3860	0.0056	0.0045	0.0008	0.0079	0.0881	166	294	9	56
Q61411	GTPase HRas	0.4180	0.4350	0.3540	0.3430	0.3650	0.0239	0.0567	0.0089	0.0384	0.0623	189	213	2	51
P13597	Intercellular adhesion molecule 1	0.5040	0.4840	0.4090	0.2600	0.4090	0.0228	0.0566	0.0136	0.0014	0.0782	537	10	5	11
P40936	Indolethylamine N-methyltransferase	0.3770	0.5480	0.3990	0.3440	0.4120	0.0035	0.0784	0.0114	0.0191	0.0872	264	108	8	33
A3KG59	Peptidase M20 domain-containing protein 2	0.5380	0.5050	0.5880	0.3840	0.4020	0.0266	0.0606	0.0945	0.0361	0.0641	431	191	12	41
Q9JKL4	NADH dehydrogenase [ubiquinone] 1 alpha subcomplex assembly factor 3	0.5160	0.3430	0.1690	0.4350	0.3980	0.0446	0.0118	0.0000	0.0911	0.0735	185	7	2	12
P16125	L-lactate dehydrogenase B chain	0.3660	0.3120	0.3730	0.2910	0.3860	0.0310	0.0193	0.0199	0.0517	0.0877	334	3262	21	74
Q91ZV0	Melanoma inhibitory activity protein 2	0.4760	0.4320	0.4070	0.4930	0.4280	0.0217	0.0420	0.0130	0.1559	0.1011	1396	11	3	2
P56375	Acylphosphatase-2	0.4230	0.3850	0.3370	0.3620	0.3660	0.0578	0.0645	0.0102	0.1381	0.0639	106	774	7	62
P20801	Troponin C, skeletal muscle	0.4840	0.3700	0.2810	0.3070	0.3620	0.0981	0.0520	0.0028	0.0672	0.0593	160	6398	12	84
Q9ESZ8	General transcription factor II-I	0.5960	0.4710	0.5590	0.3960	0.3400	0.0987	0.0469	0.1158	0.1145	0.0553	998	9	3	3
P70266	6-phosphofructo-2-kinase/fructose-2,6-bisphosphatase 1	0.5240	0.5160	0.5860	0.4680	0.4530	0.0366	0.0719	0.1011	0.1029	0.1517	471	166	12	23
Q61205	Platelet-activating factor acetylhydrolase IB subunit gamma	0.3820	0.5360	0.5660	0.3900	0.3800	0.0213	0.1809	0.1722	0.1113	0.0797	232	77	9	63

Table S3. Functional classification of proteins differentially expressed due to administration of AP-prestimulated CACs. Proteins changes between SF and SE were classified with IPA. The most probable functions of proteins of interest are shown. Legend: ↑ up-regulated; ↓ down-regulated; ▲ predicted activation increased; ▼ predicted activation decreased.

Categories.	Functions	p-value	Activation z-score	Molecules	Proteins
Cardiovascular Disease	Artery Occlusion	6,89E-07	-2,328 ▼	FABP4↑, PLIN1↑, YAP1↑, ABCA1↓, ALOX5↓, ALOX5AP↓, ARG2↓, CA13↓, CD14↓, CD44↓, CD68↓, CTSS↓, F13A1↓, FN1↓, Hrg↓, ITGAM↓, ITGB2↓, LAMP2↓, LCN2↓, LGALS3↓, MPO↓, PCNA↓, PLA2G15↓, PLIN2↓, PLTP↓, PTGES↓, S100A8↓, S100A9↓, THBS1↓	29
	Atherosclerosis	1,34E-07	-2,328 ▼	FABP4↑, PLIN1↑, YAP1↑, ABCA1↓, ALOX5↓, ALOX5AP↓, ARG2↓, CA13↓, CD14↓, CD44↓, CD68↓, CTSS↓, F13A1↓, FN1↓, ITGAM↓, ITGB2↓, LAMP2↓, LCN2↓, LGALS3↓, MPO↓, PCNA↓, PLA2G15↓, PLIN2↓, PLTP↓, PTGES↓, S100A8↓, S100A9↓, THBS1↓	28
Cell Death and Survival	Cell viability	9,78E-08	-3,278 ▼	ACLY↑, BCKDK↑, CRYAB↑, THRSP↑, YAP1↑, ABCA1↓, ACTN4↓, APAF1↓, ASS1↓, B2M↓, CAMP↓, CASP8↓, CD38↓, CD44↓, CD48↓, CYBA↓, EIF3E↓, EMILIN2↓, FLNA↓, FN1↓, HK3↓, IFIH1↓, IGHM↓, IL1RN↓, IQGAP1↓, ITGAM↓, ITGB2↓, LAMP2↓, LCN2↓, LGALS3↓, LGALS3BP↓, LGALS7/LGALS7B↓, LMNA↓, LTF↓, NCF2↓, PCNA↓, PNKP↓, POSTN↓, PRKCD↓, PRPF8↓, PRTN3↓, PTGR1↓, PTPN1↓, PTPN6↓, PTPRC↓, PYCARD↓, RBM39↓, RPL27↓, S100A8↓, S100A9↓, Saa3↓, SEL1L↓, SLC2A3↓, SMARCC2↓, SND1↓, SRC↓, STAT1↓, STAT2↓, SYK↓, THBS1↓, TRIM28↓, UBE2I↓	62
	Cell cytotoxicity	3,13E-07	-2,382 ▼	B2M↓, CD38↓, CD44↓, CD48↓, FN1↓, ITGAM↓, ITGB2↓, LGALS3↓, PLTP↓, PRKCD↓, PTPN6↓, PTPRC↓, SRC↓, STAT1↓, STX7↓, SYK↓, TAP2↓	17
Cellular Movement	Cell movement	4,63E-14	-4,512 ▼	CRYAB↑, FABP4↑, GLUL↑, YAP1↑, ABCA1↓, ABI1↓, ACTB↓, ACTN1↓, ACTN4↓, AGK↓, AIF1↓, ALOX5↓, ALOX5AP↓, AP2M1↓, APAF1↓, APBB1IP↓, ARG1↓, ARHGDIB↓, BGN↓, BIN2↓, C5AR1↓, CAMP↓, CASP8↓, CD14↓, CD38↓, CD44↓, CD48↓, CFH↓, CFL1↓, CLIC1↓, CSK↓, CTSC↓, CTSG↓, CTSH↓, CTSS↓, CTSZ↓, DDX58↓, EIF3E↓, ELANE↓, ELMO1↓, EMILIN2↓, F13A1↓, FLNA↓, FN1↓, FYB1↓, GBP2↓, GC↓, GIT2↓, GNAI2↓, HCLS1↓, Hrg↓, IFI16↓, IFIT2↓, IGHM↓, IL1RN↓, IQGAP1↓, Irgm1↓, ITGAM↓, ITGB2↓, JCHAIN↓, LCN2↓, LCP1↓, LGALS3↓, LGALS3BP↓, LGMN↓, LIPA↓, LMNA↓, LMNB2↓, LSP1↓, LTF↓, MARCKSL1↓, MBOAT7↓, MPO↓, MYL12A↓, NCF2↓, NCF4↓, NCKAP1L↓, NSF↓, OLFM4↓, Orm1↓, P2RX4↓, PARVG↓, PLTP↓, POSTN↓, PRKCD↓, PRTN3↓, PTGES↓, PTPN1↓, PTPN6↓, PTPRC↓, PYCARD↓, RCC2↓, Retnlg↓, RPL13A↓, S100A8↓, S100A9↓, Saa3↓, SKAP2↓, SLC9A3R1↓, SND1↓, SRC↓, STAT1↓, STAT2↓, SYK↓, THBS1↓, UBE2I↓, WASF2↓	107
Inflammatory Response	Inflammatory response	1,81E-16	-3,232 ▼	CSRP3↑, FABP4↑, YAP1↑, AIF1↓, ALOX5↓, ALOX5AP↓, AOAH↓, C1QTNF3↓, C5AR1↓, CAMP↓, CASP8↓, CD14↓, CD38↓, CD44↓, CFH↓, CTSG↓, CTSS↓, CYBA↓, ELANE↓, ELMO1↓, Flg↓, FN1↓, GC↓, GIT2↓, GNAI2↓, IGHM↓, IL1RN↓, ITGAM↓, ITGB2↓, LCN2↓, LCP1↓, LGALS3↓, LGALS3BP↓, LGALS9B↓, LGMN↓, LIPA↓, LSP1↓, LTF↓, Mbl1↓, MPO↓, NCKAP1L↓, Orm1↓, PLIN2↓, PRDX5↓, PRKCD↓, PRTN3↓, PTGES↓, PTPN6↓, PYCARD↓, RPL13A↓, S100A8↓, S100A9↓, SRC↓, STAT1↓, SYK↓, THBS1↓, UBE2I↓, ZBP1↓	58
	Cellular immune response	2,12E-17	-4,607 ▼	YAP1↑, ABCA1↓, C5AR1↓, CAMP↓, CASP8↓, CD14↓, CD180↓, CD38↓, CD44↓, CD48↓, CD68↓, CFH↓, CSK↓, CTSG↓, CTSS↓, CYBA↓, DDX58↓, ELANE↓, ELMO1↓, FLNA↓, FN1↓, HLA-DQB1↓, IFIH1↓, Ighg2a↓, IGHM↓, ITGAM↓, ITGB2↓, LCN2↓, LGALS3↓, LTF↓, Mbl1↓, MPO↓, PLD4↓,	49

				POSTN↓, PRKCD↓, PRTN3↓, PTPN6↓, PTPRC↓, PYCARD↓, RAB31↓, S100A8↓, S100A9↓, SRC↓, STAT1↓, SYK↓, TAP1↓, TAPBP↓, THBS1↓, TOP2A↓	
	Neutrophils immune response	6,52E-06	-2,756 ▼	CAMP↓, CD44↓, CFH↓, FN1↓, ITGAM↓, ITGB2↓, LCN2↓, SYK↓, THBS1↓	9
	Macrophages immune response	1,40E-06	-2,272 ▼	ABCA1↓, CAMP↓, CD14↓, CD38↓, CD44↓, DDX58↓, ELMO1↓, ITGAM↓, ITGB2↓, LGALS3↓, Mbl1↓, PTPRC↓, S100A9↓, THBS1↓	14
Free Radical Scavenging	Synthesis of reactive oxygen species	2,11E-15	-3,391 ▼	CRYAB↑, ABCA1↓, ACTB↓, ALOX5↓, ARG1↓, ARHGDIB↓, C5AR1↓, CAMP↓, CASP8↓, CD14↓, CD44↓, CFH↓, CLIC1↓, CSTB↓, CTSG↓, CYBA↓, ELANE↓, FLNA↓, FN1↓, HK3↓, IQGAP1↓, ITGAM↓, ITGB2↓, ITIH4↓, LGALS3↓, LIPA↓, LTF↓, MPO↓, NCF2↓, NCF4↓, PNKP↓, PRKCD↓, PRTN3↓, PTGES↓, PTPN1↓, PTPN6↓, PYCARD↓, RPL26↓, S100A8↓, SLC9A3R1↓, SRC↓, SYK↓	42

Table 4. Classification of protein changes seen between SF (mice treated with pre-stimulated CACs, SF1, SF2 and SF3) and SE (mice treated with unstimulated CACs) was made with IPA, a software based on biomedical literature and integrated databases. The table shows proteins differentially expressed related to DM; including main categories and related functions, p-value, activation z-score, molecules (protein names) and the number of proteins. Legend: ↑ up-regulated; ↓ down-regulated.

	Functions	p-value	Activation z-score	Molecules	Proteins
SF1/SE	Diabetes mellitus	1,74E-10	1,166	AACS↑, ABCA1↓, ACACA↑, AEBP1↓, AGPAT2↑, ALDH2↑, ALOX5AP↓, APOBR↓, ARG1↓, C1QTNF3↓, CA13↓, CA5B↑, CAPZA1↓, CASP8↓, CAT↑, CAVIN1↑, CD180↓, CD38↓, CD44↓, COL12A1↓, CYBA↓, CYBB↓, CYP2E1↑, ELMO1↓, EPHX2↑, FABP4↑, FAH↑, FN1↓, HCLS1↓, HLA-DQB1↓, IFI44↓, IFIH1↓, IFIT2↓, IL1RN↓, ITGAM↓, ITGB2↓, LGALS9B↓, ME1↑, MGST1↑, MOGS↓, MPEG1↓, MSR1↑, NCF2↓, PLBD1↓, PLD4↓, PLEK↓, PLIN1↑, PTPN1↓, PTPN6↓, PTPRC↓, SCD↑, Slc25a1↑, SMYD1↑, STAT1↓, STAT2↓, TAP1↓, TAP2↓, ZBP1↓	58
	Insulin-dependent diabetes mellitus	3,17E-07		ABCA1↓, ALDH2↑, ALOX5AP↓, APOBR↓, CD180↓, CD38↓, CYBB↓, HCLS1↓, HLA-DQB1↓, IFI44↓, IFIH1↓, IFIT2↓, ITGB2↓, MOGS↓, MPEG1↓, PLBD1↓, PLD4↓, PLEK↓, PTPRC↓, SCD↑, STAT1↓, STAT2↓, TAP1↓, TAP2↓, ZBP1↓	25
	Lipid conversion	6,86E-08	1,425	ABHD5↑, ACACA↑, ACLY↑, ACOX1↑, ALOX5AP↓, CAT↑, CPT1B↑, CYP2E1↑, FABP4↑, FGFR2↑, LIPE↑, PTGES↓, SCD↑, TECR↑	14
	Fatty acid conversion	1,89E-11	1,394	ACACA↑, ACLY↑, ACOX1↑, ALOX5AP↓, CPT1B↑, CYP2E1↑, FABP4↑, FGFR2↑, LIPE↑, PTGES↓, TECR↑	11
	Lipid Oxidation	6,64E-10	0,910	ABCA1↓, ABHD5↑, ACACA↑, ACADS↑, ACOX1↑, Aldh1a7↑, CAT↑, CPT1B↑, CYP2E1↑, ECI1↑, FABP4↑, HADHB↑, LIPE↑, LRPPRC↑, NUCB2↓, PLIN1↑, PNPLA2↑, SCD↑, SLC27A1↑, SLN↓	20
	Fatty acid Oxidation	7,53E-10	0,891	ABCA1↓, ABHD5↑, ACACA↑, ACADS↑, ACOX1↑, CPT1B↑, CYP2E1↑, ECI1↑, FABP4↑, HADHB↑, LIPE↑, LRPPRC↑, NUCB2↓, PLIN1↑, PNPLA2↑, SCD↑, SLC27A1↑, SLN↓	18
	Carbohydrate Metabolism	4,66E-05	1,364	ABHD5↑, AGPAT2↑, ALDH2↑, AOAH↓, CD44↓, COQ3↑, CYP2E1↑, FABP4↑, GALNS↓, GBE1↑, GFPT1↓, GNAI1↑, IL1RN↓, ITGB2↓, LIPE↑, MBOAT7↓, PHOSPHO1↑, PLA2G15↓, PLEK↓, PLIN1↑, POSTN↓, PTPN1↓, PTPRC↓, SCD↑, SFN↑, SLC27A1↑, SLC2A3↓, SLC9A3R1↓, SYK↓	29
SF2/SE	Diabetes mellitus	5,67E-10	-0,687	ABCA1↓, AIF1↓, APOBR↓, ARG1↓, ATXN2↑, B2M↓, C1QTNF3↓, C1S↓, CACNG1↓, CAPZA1↓, CASP8↓, CD180↓, CD38↓, CFH↓, COL12A1↓, COL14A1↓, CP↓, CTSH↓, CTSS↓, CYBA↓, ELMO1↓, FABP4↑, FN1↓, GBP2↓, HCLS1↓, Hrg↓, IFI16↓, IFI44↓, IFIH1↓, IFIT2↓, IGHM↓, IL1RN↓, ITGAM↓, ITGB2↓, ITIH4↓, LCP1↓, LGALS3↓, LMNA↓, ME1↑, MOGS↓, MPEG1↓, NCF2↓, OAS1↓, PDXK↓, PLBD1↓, PLD4↓, PLEK↓, PLIN1↑, PRKCD↓, PRRC2A↓, PTPN1↓, PTPN6↓, PTPRC↓, SERPINC1↓, STAT1↓, STAT2↓, TAP1↓, TAP2↓, TTR↓	59
	Insulin-dependent diabetes mellitus	1,23E-11	-1,090	ABCA1↓, AIF1↓, APOBR↓, ATXN2↑, B2M↓, CACNG1↓, CD180↓, CD38↓, CFH↓, CTSH↓, CTSS↓, GBP2↓, HCLS1↓, IFI16↓, IFI44↓, IFIH1↓, IFIT2↓, IGHM↓, ITGB2↓, ITIH4↓, LCP1↓, MOGS↓, MPEG1↓, OAS1↓, PLBD1↓, PLD4↓, PLEK↓, PRRC2A↓, PTPRC↓, STAT1↓, STAT2↓, TAP1↓, TAP2↓	33
SF3/SE	Diabetes mellitus	1,50E-13	-0,193	ABCA1↓, AEBP1↓, AIF1↓, APOBR↓, APOE↓, ARG1↓, B2M↓, C1QTNF3↓, C1S↓, CA13↓, CACNG1↓, CAPZA1↓, CASP8↓, CD180↓, CD38↓, CD44↓, CFH↓, COL12A1↓, COL14A1↓, CP↓, CTSD↓, CTSH↓, CTSS↓, CYBA↓, ELMO1↓, FN1↓, GBP2↓, GPNMB↓, HLA-DQA1↓, IFI16↓, IFI44↓, IFIT2↓, IGHM↓, IL1RN↓, Irgm1↓, ITGAM↓, ITGB2↓, LCP1↓, LGALS3↓, LGALS9B↓, LMNA↓, MOGS↓, MPEG1↓, NCF2↓, PDXK↓, PEA15↓, PLBD1↓, PLD4↓, PLEK↓, PRRC2A↓, PTPN1↓, PTPN6↓, PTPRC↓, SRC↓, STAT1↓, STAT2↓, TAP1↓, TAP2↓, ZBP1↓	59
	Insulin-dependent diabetes mellitus	3,40E-13	-1,090	ABCA1↓, AIF1↓, APOBR↓, B2M↓, CACNG1↓, CD180↓, CD38↓, CFH↓, CTSH↓, CTSS↓, GBP2↓, GPNMB↓, HLA-DQA1↓, IFI16↓, IFI44↓, IFIT2↓,	32

	IGHM↓, Irgm1↓, ITGB2↓, LCP1↓, MOGS↓, MPEG1↓, PLBD1↓, PLD4↓, PLEK↓, PRRC2A↓, PTPRC↓, STAT1↓, STAT2↓, TAP1↓, TAP2↓, ZBP1↓

Supplementary references:

1. Beltran-Camacho, L.; Jimenez-Palomares, M.; Rojas-Torres, M.; Sanchez-Gomar, I.; Rosal-Vela, A.; Eslava-Alcon, S.; Perez-Segura, M. C.; Serrano, A.; Antequera-Gonzalez, B.; Alonso-Pinero, J. A.; Gonzalez-Rovira, A.; Extremera-Garcia, M. J.; Rodriguez-Pinero, M.; Moreno-Luna, R.; Larsen, M. R.; Duran-Ruiz, M. C., Identification of the initial molecular changes in response to circulating angiogenic cells-mediated therapy in critical limb ischemia. *Stem Cell Res Ther* **2020,** 11, (1), 106.
2. Garcia, S.; Marston, N.; Sandoval, Y.; Pierpont, G.; Adabag, S.; Brenes, J.; Santilli, S.; McFalls, E. O., Prognostic value of 12-lead electrocardiogram and peak troponin I level after vascular surgery. *J Vasc Surg* **2013,** 57, (1), 166-72.
3. Tarlov, I. M., Spinal cord compression studies. III. Time limits for recovery after gradual compression in dogs. *AMA Arch Neurol Psychiatry* **1954,** 71, (5), 588-97.
